# Molecular signature of stem-like glioma cells (SLGCs) from human glioblastoma and gliosarcoma

**DOI:** 10.1371/journal.pone.0291368

**Published:** 2024-02-02

**Authors:** Christina Zechel, Mira Loy, Christiane Wegner, Eileen Dahlke, Birga Soetje, Laura Baehr, Jan Leppert, Johannes J. Ostermaier, Thorben Lueg, Jana Nielsen, Julia Elßner, Viktoria Willeke, Svenja Marzahl, Volker Tronnier, Amir Madany Mamlouk

**Affiliations:** 1 Laboratory of Experimental Neuro-Oncology, Center of Brain, Behavior and Metabolism, University Lübeck, Lübeck, Germany; 2 Department of Neurosurgery, University Clinic Schleswig-Holstein, Campus Lübeck, Lübeck, Germany; 3 Institute for Neuro- and Bioinformatics (INB), University Lübeck, Lübeck, Germany; New York University Langone Health, UNITED STATES

## Abstract

Glioblastoma multiforme (GBM) and the GBM variant gliosarcoma (GS) are among the tumors with the highest morbidity and mortality, providing only palliation. Stem-like glioma cells (SLGCs) are involved in tumor initiation, progression, therapy resistance, and relapse. The identification of general features of SLGCs could contribute to the development of more efficient therapies. Commercially available protein arrays were used to determine the cell surface signature of eight SLGC lines from GBMs, one SLGC line obtained from a xenotransplanted GBM-derived SLGC line, and three SLGC lines from GSs. By means of non-negative matrix factorization expression metaprofiles were calculated. Using the cophenetic correlation coefficient (CCC) five metaprofiles (MPs) were identified, which are characterized by specific combinations of 7–12 factors. Furthermore, the expression of several factors, that are associated with GBM prognosis, GBM subtypes, SLGC differentiation stages, or neural identity was evaluated. The investigation encompassed 24 distinct SLGC lines, four of which were derived from xenotransplanted SLGCs, and included the SLGC lines characterized by the metaprofiles. It turned out that all SLGC lines expressed the epidermal growth factor EGFR and EGFR ligands, often in the presence of additional receptor tyrosine kinases. Moreover, all SLGC lines displayed a neural signature and the IDH1 wildtype, but differed in their p53 and PTEN status. Pearson Correlation analysis identified a positive association between the pluripotency factor Sox2 and the expression of FABP7, Musashi, CD133, GFAP, but not with MGMT or Hif1α. Spherical growth, however, was positively correlated with high levels of Hif1α, CDK4, PTEN, and PDGFRβ, whereas correlations with stemness factors or MGMT (MGMT expression and promoter methylation) were low or missing. Factors highly expressed by all SLGC lines, irrespective of their degree of stemness and growth behavior, are Cathepsin-D, CD99, EMMPRIN/CD147, Intβ1, the Galectins 3 and 3b, and N-Cadherin.

## Introduction

The glioblastoma multiforme (GBM) and its variants represent the most abundant malignant brain tumors [1]. The advanced classification of tumors of the central nervous system combines clinical and histopathological criteria with the presence of genetic and molecular markers [2–5]. The standard therapy, including surgical resection followed by fractionated radiation and adjuvant chemotherapy with Temozolomide® (TMZ) remains largely unchanged since 2005 and provides only palliation [6, 7]. The gliosarcoma (GS), which is either considered a rare GBM variant or a separate tumor identity, is treated according to the guidelines for GBMs, though many histopathological and molecular features are distinct [8–11].

Several parameters have been identified that contribute to the therapy resistance of GBMs and presumably also GSs. In addition to the TMZ concentrations in brain parenchyma, TMZ dosing schemes and the blood brain barrier, detoxifying proteins such as the O^6^-methylguanine-DNA-merthyltransferase (MGMT) or ABC (ATP-binding cassette) transporters, compromise therapy efficacy [12–14]. Moreover, molecular characterization of GBM specimens from several hundred patients led to the definition of GBM subtypes with distinct combinations of founder mutations and epigenetic changes, providing evidence for an intertumor and intratumor heterogeneity [15–19]. Phillips et al. [20] defined three GBM subtypes, referred to as proliferative, mesenchymal, and proneural, whereas Verhaak et al. [21] further subdivided the proliferative subtype into a classical and neural subtype and integrated the so-called GCimp GBM into the proneural group. Biomarkers used in the discrimination between subtypes included PTEN loss, IDH1 mutation, PDGFRα and EGFR dysregulation, p53 abnormalities, and MGMT promoter methylation. Moreover, dysfunction of the cyclin-dependent kinases 4 and 6 appeared common in high-grade gliomas [5]. Additional factors used for GBM classification were CD133, GFAP, DLX2, MAP2, MELK, Nestin, PE-CAM, VEGFR-1/-2, and Vimentin among others [20, 21].

Apart from being used in the classification of GBM subtypes, CD133, DLX2, and Nestin have additionally been applied in the characterization of glioma cells with similarities to neural stem cells, which are referred to as, e.g., BCPCs, CSCs, GSCs, or SLGCs [12, 16, 22–25]. Many lines of evidence support the crucial role of SLGCs in glioma initiation, progression, therapy resistance, and relapse [15–17, 24, 25]. The first reports characterizing SLGCs used fluorescence-activated cell sorting and antibodies directed against the glycoprotein CD133. Later on, it became evident that CD133-positive and certain CD133-negative cells possess the capacity to self-renew (overview in [24, 25]). SLGCs, which constitute only a small subpopulation of the GBM cells, express the pluripotency factor Sox2 and the intermediate filament Nestin [12, 22–26]. A model explaining the limitations of CD133 in SLGC research [27] was proposed by Chen et al. in 2010 [28]. This paper provided evidence for the presence of three types of self-renewing GBM cells, in which the Sox2+/CD133- type I cell represents the glioma stem cell, which may further differentiate to generate Sox2+/CD133+ type II and Sox2(+)/CD133- type III cells, which may be considered early and late progenitors, respectively. Unlike the bulk of tumor cells, all three types of self-renewing cells (type I, II and III) possess the capacities to differentiate, sustain tumor growth, and generate orthotopic tumors in mice [28]. A genetically modified mouse model proved that undifferentiated Nestin-positive GBM cells propagated tumor growth after TMZ treatment [29]. Additional research indicated that SLGCs are more resistant to radio- and chemo-resistance than the bulk of tumor cells [30–32].

In spite of the increasing knowledge about the significance of SLGCs for GBM initiation, progression, therapy resistance, and relapse, a strategy to specifically eliminate type I-III cells does not exist yet. Since this is related to the lack of a common SLGC marker and the intertumor and intratumor heterogeneity of malignant gliomas, we compared the molecular signatures of SLGCs from 17 human GBMs, 4 orthotopic GBMs, and 3 GSs.

## Materials and methods

### Tumor specimen and mouse model

Experiments with tumor specimens were performed according to the Helsinki guidelines, in compliance with national regulations for the experimental use of human material (vote 08–070 of the Ethics Commission at the University of Lübeck). Glioblastoma multiforme (GBM) and gliosarcoma (GS) specimens were obtained from patients who underwent routine tumor resection and expressed their agreement to participate in the study. The classification of tumor type and grade was according to WHO criteria and carried out by a neuropathologist. Tumor specimens were made anonymous, and the cell lines derived thereof were designated “T” followed by the code number of the tumor. Only late-onset tumors were included in the study.

### Cell culture and limited dilution assay

Stem-like glioma cells (SLGCs) were cultured in N-medium (DMEM/Ham’s F12 (Biochrom, Berlin, Germany) containing 20% BIT 9500 serum-free supplement (PELOBiotech, Planegg, Germany), 2% of a 200 mM Glutamine solution, 1% Amphotericin, 1% standard Penicillin/Streptomycin mix, and 20 ng/ml of recombinant human EGF and bFGF (Promo Cell, Heidelberg, Germany)). In order to induce differentiation, SLGC lines were transferred into DMEM/Ham’s F12 containing 10% FCS but lacking EGF and bFGF (referred to as F-medium). For limited dilution assays, 500–800 cells were plated in 96 well plates, as only 5–10% of the cells from our SLGC lines survive this condition. Typically, primary clones formed spheres, which were enzymatically dissociated, followed by stepwise propagation in larger formats. Before further use in research, the expression of stemness markers was monitored by immunocytochemistry.–The established human glioblastoma cell line U87-MG, referred to as U87 in the manuscript, and the established human colon cancer cell line CaCo2 were grown in DMEM (supplemented with 1% Amphotericin, 1% standard Penicillin/Streptomycin mix) containing 10% and 20% FCS, respectively. All cell cultures were incubated at 37°C in a water-saturated atmosphere in the presence of an air-carbon dioxide (5% CO_2_) mixture.

### Animal experiments

Animal experiments were performed according to the guidelines of the European Communities Council Directive (86/609/EEC) and the national animal welfare regulation with the positive vote of the minister of agriculture, ambience, and rural environment Schleswig-Holstein (Kiel; V242-72241.122-13(77-8/08).—To determine whether the glioma-derived primary cell lines or the clones would be tumorigenic, we used the SCID mouse model (SHO-PrKdc^SCID^Hr^h^, Charles River Research Laboratories, Sulzfeld, Germany). Anesthesia was performed with 8% Rompun (Xylazine hydrochloride, 2%, Bayer, Germany) and 12% Ketavet® (Ketamine hydrochloride, 100 mg/ml Pfizer, Karlsruhe, Germany) in a 0.9% sodium chloride solution. Inoculation of cells was done into the right hemisphere using a stereotactic frame and a Hamilton syringe. Wound closure and suture were carried out with Ethilon*II 3–0 (Johnson & Johnson Intl., St.Stevens-Woluwe, Belgium). During all steps, hypothermia was prevented by an infrared lamp. Immunosuppressed out-bred SCID mice (minimum of six females; 6–8 weeks of age) were transplanted with 50,000, 100,000, and 200,000 cells. Animals were observed on a daily basis and sacrificed after 12 weeks. After two initial series of experiments, T1338-transplanted mice were sacrificed after 6 months. Euthanasia was performed using carbon dioxide; animals developing any health threats were sacrificed before the scheduled time point. Mouse brains were fixed for 24 h in 4% paraformaldehyde, followed by transfer into 70% ethanol, paraffin-embedding, and HE (hematoxylin/eosin) staining according to standard protocols. In cases in which a macroscopically visible tumor was present, a piece of the orthotopic tumor was used to establish a cell line in N-medium. The respective cell lines were named as the parental cell lines plus the suffix “SC” or “SC2” indicating a first and second expansion in the mouse brain, respectively.—A subgroup of xenotransplanted mice was studied using Magnetic Resonance Imaging (MRI). These mice were sedated with a low dose of 12% Ketavet (see above) and fixed in a prone position with a coil-loop and modeling clay. MRI was done on a 3-Tesla clinical routine MR scanner using a two-loop microscopy surface coil (diameter = 47 mm) for signal reception (Achieva 3T, Philips Health Care, Hamburg, Germany). Two 3D data sets were acquired with a spatial resolution of 190 μm x 190 μm x 300 μm, one T1-weighted and another T2-weighted. The T1 data set was recorded in turbofield echo technique (TFE) with TR = 23 msec and TE = 11 msec. The T2-weighted images were measured in turbo spin echo (TSE) with a repetition time (TR) of 2000 msec and an echo time (TE) of 94 msec.

### Growth curves and ELISAs

***Growth curves*** (d0 –d20) were performed in triplicate in 24 well plates using 5 x 10^3^ cells/cm^2^. In order to exclude side effects induced by high cell densities, one half of the cell batches were trypsinized on day d6 and re-plated (1 x 10^4^ cells/cm^2^). Counting was performed in the presence of Trypan blue (Seromed, Berlin, Germany) using a Neubauer counting chamber. ***BrdU ELISA*** was performed with fibronectin-coated plates encompassing a minimum of eight replicates per 96-well plate. Coating with fibronectin (5 μg/ml; Promo Cell, Heidelberg, Germany) was performed at a dilution of 1:100. The *BrdU Labeling and Detection Kit III* (Roche, Mannheim, Germany) was used according to the manufacturer’s instructions. ***Growth factor ELISA*:** Cell culture supernatant was collected from exponentially growing cultures. Cell debris was removed by centrifugation (10 min, 20,000 x g, 4°C). Growth factor contents were determined using the *human EGF and the human bFGF Quantikine ELISA* Kit (R&D Systems, Abingdon, UK) according to the manufacturer’s instructions. The assays encompassed a minimum of two triplicates.

***Flow cytometry*** was carried out using fluorophore-coupled antibodies or suitable isotype controls (Miltenyi Biotech). Antibody binding was performed in the presence of an FcR blocking reagent (human; Miltenyi Biotech). The colon carcinoma cell line CaCo2 (>90% CD133+ cells) served as a reference. Flow cytometry was carried out at the CAnaCore cell sorting facility at the University Clinic Schleswig-Holstein, Campus Lübeck. The software program Summit v4.3 was applied for analysis. The following antibodies were used: αCD51/Integrin-αv -FITC (mouse, IgG1, P1F6 κ-FITC MOPC-21; Abcam; Cambridge, UK); α CD49f/Integrin-α6 (rat, IgG2; BV421; clone GoH3; BD Biosciences; Heidelberg, Germany) as well as αCD15/SSEA-1-APC (My; αCD31/PECAM-PE (mouse IgG1, AC128); αCD44-PE (mouse IgG2b, DB105); αCD133(Prominin-1)2-PE (mouse IgG2b, clone 293C3; epitope 2); αCD144/VE-Cadherin-PE (mouse IgG2b) (Miltenyi Biotech; Bergisch Gladbach, Germany).–Isotype controls: αIgG1 (mouse); αIgG2B (mouse) APC-, PE- and/or FITC-coupled (Abcam, Miltenyi, or BD BioSciences).

### Immunological stains

Primary and secondary antibodies are listed below. All stains were observed with the BZ8000 (Keyence, Neu-Isenburg, Germany) and the BZ9000 software. Microphotographs are Z-stacks of up to 80 individual photos (pitch 0.2–0.3).***–Immunohistochemistry*** (IHC) was performed with human tumor biopsies and orthotopic tumors from SCID mice. Paraffin-embedded material was sliced at 4.5 μm using a microtome (Leica RM2245; Solms, Germany). Sections underwent deparaffination, followed by antigen demasking and staining with antibodies and DAPI according to standard protocols. In order to identify human tumor cells in mouse brains we used the human-specific antibody αStem121. Hematoxylin and eosin (HE) stains were performed according to standard protocols, using Entellan (Merck, Darmstadt, Germany) for mounting. In the case of tumor slices, GBM specimens were embedded in low-melting agarose and sliced with a vibratome (Leica) at 200 μm. Fixation of slices was done with 4% PFA for 5 min at room temperature, followed by treatment with 20% Methanol and 1% Triton X100 for 16 h at 4°C. Staining was in the presence of 10% FCS.–***Immunocytochemistry*** (ICC) of adherent cells and aggregates was carried out using glass coverslips (Roth, Karlsruhe, Germany) or chamber slides (Thermo Fisher Scientific, Schwerte, Germany). Tumor spheres were stained in suspension. Fixation was performed for 7 minutes at -20°C, using a mixture of ethanol/acetic acid (95:5 v:v). Incubation with antibodies was carried out according to the manufacturer’s instructions. All assays included a nuclear counterstain with DAPI (Roth). Fluoromount-G (Southern Biotechnologies, Birmingham, USA) was used for mounting. IHC and ICC analyses encompassed a minimum of three replicates.

***Antibodies*** (symbolized by α) for IHC, ICC and Western blots.*—***Primary antibodies**: αAADC/DDC (αDOPA-Decarboxylase; rabbit; Cell Signaling, Danvers, USA); αABCG2 (mouse, ICC; (BXP-21); Abcam Cambridge, UK); αABCG2 (rabbit, WB (isoforms 1 and 2: 72.3 kDa and 67.5 kDa); (EPR2099(2)); Abcam); αActin (mouse; pan-Actin antibody; MAB 1501R, WB; Merck, Darmstadt, Germany); αAKT (rabbit; C67E7, detects AKT1, AKT2 and AKT3, 55.7–55.8 kDa; Cell Signaling); αCD15/SSEA-1 (mouse, MC813, ICC; Cell Signaling); αCD44 (mouse, 156-3C11, ICC/IHC, WB; Cell Signaling); αCD133/Prominin-1 (rabbit; clone C24B9 (directed against epitope around Asp562), ICC, WB (distinct isoforms ~120 kDa); Cell Signaling); αCD133/Prominin-1 (mouse, (W6B3C1, epitope 1), ICC, WB; Miltenyi Biotech (Bergisch Gladbach, Germany); αCDK4 (mouse, (DCS156), WB (isoform 1: 33.7 kDa); Cell Signaling); αCDK6 (mouse, (DCS83), WB; Cell Signaling); αDARPP32 (rabbit, WB; Abcam, Cambridge, UK); αDLX2 (rabbit; US Biological, USA); αE-cadherin (rabbit; Abcam; Cambridge, UK); αEGFR1-antibody (rabbit, WB (HER1 isoform 1: 134.3 kDa; isoforms 2: 44.7 kDa; isoform 3: 77.3 kDa; isoform 4: 69.2 kDa); Cell Signaling); αFABP7 (rabbit; WB; Imgenex, San Diego, USA); αGFAP (mouse, MAB360, ICC/IHC, WB (several isoforms, largest isoforms 49.9–57.4 kDa); Merck), αGFAP (rabbit AB5804, ICC/IHC; Merck), αGAPDH-POD (rabbit, peroxidase-coupled Ab 3683, WB; Cell Signaling); αHIF1α (rabbit, WB; Abcam); αIDH1 (rabbit; D2H1, WB; Cell signaling); αIDH2 (rabbit; D8E3B, WB; Cell signaling); αIntegrin-αv (rabbit, WB; Cell Signaling); αIntegrin-α6 (rabbit, WB, ab112181; Abcam); αIntegrin-β1 (rabbit, WB; Cell Signaling); αIntegrin-β3 (rabbit, WB; Cell Signaling); αIntegrin-β5 (rabbit, WB; Cell Signaling); αMAP2 (mouse, ICC; Merck); αMGMT (mouse, Merck, Darmstadt, Germany); αMERTK (mouse, 25H2, WB; Cell Signaling); αMusashi (rabbit; WB; Merck); αNanog (rabbit; WB; Imgenex); αN-Cadherin (rabbit; Cell Signaling); αNotch-1 (rabbit; clone D1E11, WB; Cell Signaling); αNestin (mouse, clone 10C2; human-specific, ICC/IHC; Merck); αNF (WB, mixture of antibodies directed against the three neurofilaments NF-L, NF-M and NF-H; AbD Serotec, Puchheim, Germany); αp53 (rabbit, ICC, WB (isoform 1: 43.65 kDa: migrates at ~53 kDa position in SDS-PAGE; isoforms 2–4: 37.8–39.32); Cell signaling); αPax6 (rabbit; WB; Abcam); αPDGFRα (rabbit, (D1E1E), ICC, WB (isoform 1: 122.7 kDa); Cell Signaling); αPDGFRβ (rabbit, (C82A3), ICC, WB (isoform 1: 123.9 kDa); Cell signaling); αPTEN (rabbit; 138G6, WB (canonical isoform: 47. 2 kDa); Cell Signaling); αSox2 (rabbit; clone D6D9 (against region surrounding Gly179), ICC/IHC, WB; Cell Signaling); αSox2 (mouse; (9-9-3), ICC; Abcam); αSox2 (rabbit, WB (38–42 kDa); Merck); Stem121 (human cytoplasmic protein) Ab121-U-050; ICC: 1:100; StemCells Inc, UK)); αTau (rabbit, ICC, WB; Dako, Hamburg, Germany); α−alpha-Tubulin-POD (rabbit, peroxidase-coupled Ab 9099, WB; Cell Signaling); αVimentin (Ab16700, ICC; Abcam); α58K (mouse; mAb-58K-9; ICC; Abcam).—**Secondary antibodies** (ICC, IHC, WB): goat anti-mouse DyLight® 488 (ICC/IHC, Thermo Fisher Scientific; Loughborough, UK), goat anti-rabbit Cy3 (ICC/IHC; Jackson Immuno-Research, Newmarket, Suffolk, UK); goat anti-mouse-POD (Goat F(ab’)2 Fragment Anti-Mouse IgG (H+L) Peroxidase, WB, Beckman Coulter; Krefeld, Germany); goat anti-rabbit-POD (Goat F(ab’)2 Fragment Anti-rabbit IgG (H+L) Peroxidase, WB; Beckman Coulter), goat anti-rabbit HRP (WB, Cell Signaling Technology).

### Western blot analysis (WB)

Cells were harvested in 1 ml of TEN (10 mM Tris-HCl (pH 7.5), 1 mM EDTA (pH 8.0), 150 mM NaCl). Protein extraction was performed with a buffer containing 50 mM Tris-HCl (pH 7.5), 150 mM NaCl, 10% glycerol (v:v), 0.5% Triton-X100 (v:v), 1% PMSF (Phenylmethylsulfonylfluorid; w:v) and a protease inhibitor cocktail (PIC; Roche, Mannheim, Germany). Cell debris was removed by a high speed clearing spin. 15 and 20 μg (ABCG2, AKT, cadherins, CD44, CDKs, EGFR, FABP7, GFAP, IDHs, MERTK, Musashi, Notch, neurofilaments, PTEN, PDGFRα, PDGFRβ, Sox2, Tau, Tp53, β-and α-integrins (except for Integrin-α6)), 30 μg (AADC/DDC, CD133, DARPP32, Hif1α, Nanog, Pax6) and 40 μg (Integrin-α6, CD15/SSEA-1) of whole cell extracts (WCE) were resolved by SDS-PAGE (Minigel Protean III, BioRad, Munic, Germany) and transferred onto nitrocellulose membranes (0.45 μm, BioRad) using the Mini Protean Transfer system (BioRad; for Integrins) or the Semidry Transfer System TransBlot SD (BioRad; all other proteins). Electrophoresis was performed with run buffer (248 mM Trizma Base, 400 mM glycine, 0.5% SDS); the transfer buffer contained 25 mM Trizma base (pH 8.3), 250 mM glycine, 0.3% SDS (w:v), and 20% methanol (v:v). 10% resolving gels were used, except for integrins, neurofilaments and RTKs (8%); stacking gels contained 4% polyacrylamide. Actin (pan), Tubulin (alpha) and/or GAPDH (glyceraldehyde 3-phosphate dehydrogenase) served as loading controls. Detection was carried out with Super Signal® West Dura Extended Duration Substrate (Thermo Fisher Scientific, Loughborough, UK), using the ChemiDoc XRS (BioRad) and the software Quantity One (BioRad). Western blots were carried out with WCEs from a minimum of three distinct passages.–Raw data are depicted in the S1 Raw images.

### Proteome analysis

Cells from identical or very similar passages were grown in 10 cm cell culture dishes and harvested with a rubber policeman and/or by centrifugation. The *Proteome Profiler*^*TM*^ *Array “Human Soluble Receptor Array Kit—Non-Hematopoietic Panel*” (R&D Systems, Abingdon, UK) was used according to the manufacturer’s instructions. Protein extraction was performed with the lysis buffer included in the kit. 250 μg WCE were used per membrane. Signals were revealed using the *Pierce ECL Western Blotting Substrate* (Thermo Fisher Scientific, Loughborough, UK). Chemiluminescence was detected and quantified as described above.

### *RT-PCR (reverse transcriptase PCR) and qRT-PCR* (quantitative real time RT-PCR)

Extraction of total RNA was performed using the RNeasy Mini-Kit (Qiagen), followed by a DNase I-treatment (Ambion, Darmstadt, Germany), extraction with phenol-chloroform (Roth) and ethanol (Merck) precipitation according to standard protocols [33]. 100 ng RNA were reverse-transcribed using the *AMV* (Avian Myeloblastosis Virus) *first-strand cDNA synthesis kit* (Roche, Mannheim, Germany), followed by standard PCR (second strand synthesis) and TBE/agarose gel electrophoresis [33]. Raw data are depicted in the S1 Raw images.

For qRT-PCR, cDNA synthesis was performed with the *iScript*^*TM*^ *Reverse Transcription Supermix for RT-qPCR* kit (BioRad, Munic, Germany), followed by second strand synthesis with the qPCR Core kit for SYBR® Green I kit (Eurogentec, Cologne, Germany), and monitoring of product synthesis with the CFX96 Real-time PCR Detection System (BioRad). Quantification of the PCR products was relative to products obtained with primers for the human reference genes *gapdh*, *ubiquitin ligase*, *and* 18s *r-rna*. The relative expression was calculated according to the ΔΔ-CT method. Analyses were performed in triplicate, using RNA from two biological replicates.—DNA-oligonucleotides were purchased from Eurofins Genomics (Ebersberg, Germany): bFGF, (F: 5’-AGA GCG ACC CTC ACA TCA AG-3’; R: 5’-GTT TCA GTG CCA CAT ACC AAC-3’); E-cadherin (F: 5’- CAC ACT GAA AGT GAC TGA TGC-3’; R: 5’-GCT ACG TGT AGA ATG TAC TGC-3’); N-cadherin (F: 5’-TAT GAG TGG AAC AGG AAC GC-3’; R: 5’-GAT CAA TGT CAT AAT CAA GTG C-3’); VE-cadherin (F: 5’-TGT GGG CTC TCT GTT TGT TG-3’; R: 5’-AAT GAC CTG GGC TCT GTT TC-3’); EGF (F: 5’-ACG CCC TAA GTC GAG ACC G-3’; R: 5’-AAT CCT ACA GGG CAC GTG C-3’); egfr/her-1 (F: 5’-GGT GGT CCT TGG GAA TTT GG-3’; R: 5’-GAC TAT GTC CCG CCA CTG G- 3’); HB-EGF (F: 5’-GAC TGG CGA GAG CCT GGA GC-3’; R: 5’-TGG TGT GGC CAG TGC TTG TGG-3’); gapdh (F: 5’-GAG TCA ACG GAT TTG GTC GT-3’; R: 5’-GGA AGA TGG TGA TGG GAT TT-3’); integrin-α1 (F: 5’-GAT TGT CAT CTT CTG AGA ATG C-3’; R: 5’-TGA GAT TAC ATG TGA TGG TAG C-3’); integrin-α2 (F: 5’-ACT CAC TTT GTT GCT GGT GC-3’; R: 5’-GAG CAC GTC TGT AAT GGT G-3’); integrin-α3 (F: 5’-TGC TGG TGC ATC ACG GAT G-3’; R: 5’-GGT GTC ACC ACA TTG CAG G-3’); integrin-α4 (F: 5’-GCT TCT CAG ATC TGC TCG TG-3’; R: 5’-GTC ACT TCC AAC GAG GTT TG-3’); integrin-α5 (F: 5’-CTG AGC TGT GAC TAC TTT GC-3’; R: 5’-GAT TCT TGC TGA GGA TCT GG-3’); integrin-α6 (F1: 5’-TTG AAT ATA CTG CTA ACC CCG-3’; R1: 5’-TCG AAA CTG AAC TCT TGA GGA TAG-3’); integrin-α8 (F: 5’-CAA GGA CAA GTG ATC ACT GC-3’; R: 5’-GTA ACT GTC ATC ATA GGA AGC-3’); integrin-αv (F: 5’-AAT CTT CCA ATT GAG GAT ATC AC-3’; R: 5’-AAA ACA GCC AGT AGC AAC AAT-3’); integrin-β1 (F: 5’-AAC TAC ACT GGC AGT GCA TG-3; R: 5’-GCT CAG CAC AGA CAC CAA G-3’); integrin-β3 (F: 5’-GGT GAC TAT GGA GCT GAG C-3’; R: 5’-CAT AGC TGC TGA GAG TCA GG-3’); integrin-β5 (F: 5’-CTG CTC GAC AGA CAT CAG C-3’; R: 5’-CTT GGT GCT GCA TGC ATC C-3’); mgmt: (F: 5’-CTG GCT GAA TGC CTA CTT CC-3’; R: 5’- CAA CCT TCA GCA GCT TCC AT-3’); PTEN (F: 5’-AAA CAG TAG AGG AGC CGT C-3’; R: 5’-GAC TTT TGT AAT TTG TGT ATG C-3’); TGFα (F2: 5’-ATA TCA CAT GAA GAC CCT AGC-3’; R2: 5’-ATC ATC TCC AAG GGT GGC G-3’); p53 (F: 5’-CTG CCC TCA ACA AGA TGT TT-3’; R: 5’-CTC AAA GCT GTT CCG TCC CA-3’); 18s r-*rna* (F: 5’- AGT CCC TGC CCT TTG ACA CA-3’; R: 5’-GAT CCG AGG GCC TCA CTA AAC-3’); ubiquitin ligase (F: 5’-ATT TGG GTC GCG GTT CTT G-3’; R: 5’-TGC CTT GAC ATT CTC GAT GGT-3’).

### Sequencing

Gel-purified PCR-products (NucleoSpin® Gel & PCR Clean-up Kit; Macherey-Nagel, Düren, Germany) were used for sequencing. Sequencing was performed with the *Brilliant Dye TM Terminator (v3*.*1) cycle sequencing* kit and the 5x sequencing buffer (both Nimagen B.V., Nijmegen, Netherlands). Separation of the sequencing products was by capillary electrophoresis using the Applied Biosystems™ 3130/3130 Genetic Analyzer (3130 Series Data Collection Software 4; Thermo Fisher Scientific, Schwerte, Germany; at the Department of Paediatric and Adolescent Medicine, University Lübeck). For sequence analysis, we used the *Chromas* software and the ENSEMBL genome browser.–Primer (purchased from Eurofins genomics; Ebersberg, Germany) used in preparation of PCR fragments for sequencing and subsequent sequencing: Tp53 exon 5 (F: 5‘-CTT TAT CTG TTC ACT TGT GCC C-3‘; R: 5 ‘-CAA CCA GCC CTG TCG TCT C-3‘); Tp53 exon 6 (F: 5‘-GAC GAC AGG GCT GGT TGC C-3‘; R: 5‘-TTA ACC CCT CCT CCC AGA GA-3‘); Tp53 exon 7 (F: 5‘-ACT GGC CTC ATC TTG GGC CT-3‘; R: 5‘-GTG CAG GGT GGC AAG TGG C-3‘); Tp53 exon 8 (F: 5‘-TTA AAT GGG ACA GGT AGG ACC-3’; R: 5‘-TCC ACC GCT TCT TGT CCT GC-3‘); IDH-1 (F: 5’-CCA ACG ACC AAG TCA CCA AG-3’; R: 5’- TGT TGA GAT GGA CGC CTA TTT G-3’).

### MSP (methylation-specific PCR)

Methylation of GpC islands in the *mgmt* promoter was studied in tumor tissue and SLGC lines (mc) and clones (cl) obtained by limited dilution assays. Genomic (g)DNA was extracted by means of the DNeasy Blood & Tissue Kit (Qiagen, Hilden, Germany). We used the CpGenome DNA Modification Kit (Chemicon, USA) for bisulfite modification of gDNA and subsequent purification. Bisulfite modification of unmethylated Cytosin (C) yielded Uracil (U), whereas 5-methyl Cytosine (^5m^C) remained untouched. This was revealed by PCR with primers, which permit the detection of modified and unmodified *mgmt* promoter regions, respectively. Primers specific for the methylated (m-) status: MGMT^meth^-F (5‘-GTT TTT AGA ACG TTT TGC GTT TCG AC-3’); MGMT^meth^-R (5‘-CAC CGT CCC GAA AAA AAA CTC CG-3‘); Primers specific for the un-methylated (u-) status: MGMT^un^-F (5‘-TGT GTT TTT AGA ATG TTT TGT GTT TTG AT-3’); MGMT^un^-R (5‘- CTA CCA CCA TCC CAA AAA AAA ACT CCA-3’). MSP analyses of SLGC (mc) lines included a minimum of three biological replicates from increasing passages; in the case of tumor tissue and clones, at least three independent technical replicates of the same gDNA were studied. PCR products were separated on 2.0% TBE-agarose gels; quantification of signal strength was performed with the ChemiDoc XRS (BioRad, Munich, Germany) and the software MicroWin 2000 (Mikrotek Laborsystem GmbH, Overrath, Germnay).–Raw data of the gels are depicted in the S1 Raw images. It has to be noted that MSP analyses with the primers specifying the u-status may show small variations, due to the degree of promoter methylation or slight variations in the efficacy of the Cytosine to Uracil conversion.

### Statistics, cophonetic correlation coefficients and Pearsons correlation coefficient

ANOVA with post-hoc Tukey HSD calculation was used to determine significant differences between optical densities (BrdU ELISA, growth factor ELISA), growth curves and mRNA expression (RT-PCR and qRT-PCR).–***For non-negative matrix factorization (NMF)***, the expression data X of the cell surface proteins determined with the R&D proteome profiler is factorized into two smaller matrices W and H, leading to a low-rank approximation: *X*≈*WH*. For *m* objects with *n* features the (*n×m*) data matrix X is approximated by *k<<min(n*,*m)* feature vectors in W^n*k^ (which are called metaprofiles in this manuscript) and corresponding weights in matrix H^k*m^ (which are the coefficients to reconstruct the original data set X with W). As all entries in X, W and H have to be non-negative, the objects are represented as a non-negative linear combination of feature vectors that can be interpreted as parts of the objects (e.g., parts of a human face, like the nose, eyes, or mouth) [34]. We used the NMF package by Brunet *et al*. [35] for our analysis. Their technique was already used successfully for clustering information from cancer-related microarray data [35]. The update formula for W and H used in the NMF algorithm minimizes the divergence functional $D(X||WH)=\sum_{ij}\left(X_{ij}log(X_{ij}/(WH{)}_{ij}\right)-X_{ij}+(WH{)}_{ij})$, which is related to the Kullback-Leibler divergence [65, Algorithm paper]. For the model selection k, we used the cophenetic correlation coefficient (CCC) to evaluate the robustness of a k-factorial NMF decomposition as proposed by [35]. The more pairwise data is recombined through the same metaprofiles (MPs) in each pass, the higher the CCC score is. Each of the MPs has a (pseudo) expression profile over the measured proteins, i.e., the coefficients in H. We filtered out all proteins that show an expression above 90% percentile (or above 95% percentile, respectively) and plotted them for each MP (see Fig 4A). In addition, we visualized how good the reconstruction X’ = W*H of the initial data X is by reducing the MPs to the proteins from the upper percentiles (90% and 95%)—and setting the other entries to 0 (see, S2 Fig). Finally, the mean squared reconstruction error of the original data was compared with two unrelated, non-SLGC lines too in order to crossvalidate the SLGC-specific metaprofiles. For this, the proposed coding alphabet (i.e., the five metaprofiles) for 1000 repetitions was used. To ensure that the increase in MSE is not a bias induced by the fact that training and test data were compared, the expected test error for similar assays with a leave-2-out cross validation (L2CV) was estimated. The protein extracts used for CCC analysis were additionally used for Western blot analysis.

Pearsons correlation coefficient calculation was performed in order to compare the relative expression determined by western analysis, RT-PCR, flow cytometry, and growth behavior, respectively. For western blot data: Actin-normalized expression levels obtained with the same protein extracts were compared to each other. This included a minimum of three independent biological replicates from passages p4-9 (set I), p12-18 (set II) and p20-30 (set III). Additionally, higher passages (p35 –p65) were included for T1371, T1440, T1447, and T1522.

## Results

### Features of SLGCs from distinct GBMs and GSs

**Fig 1:**
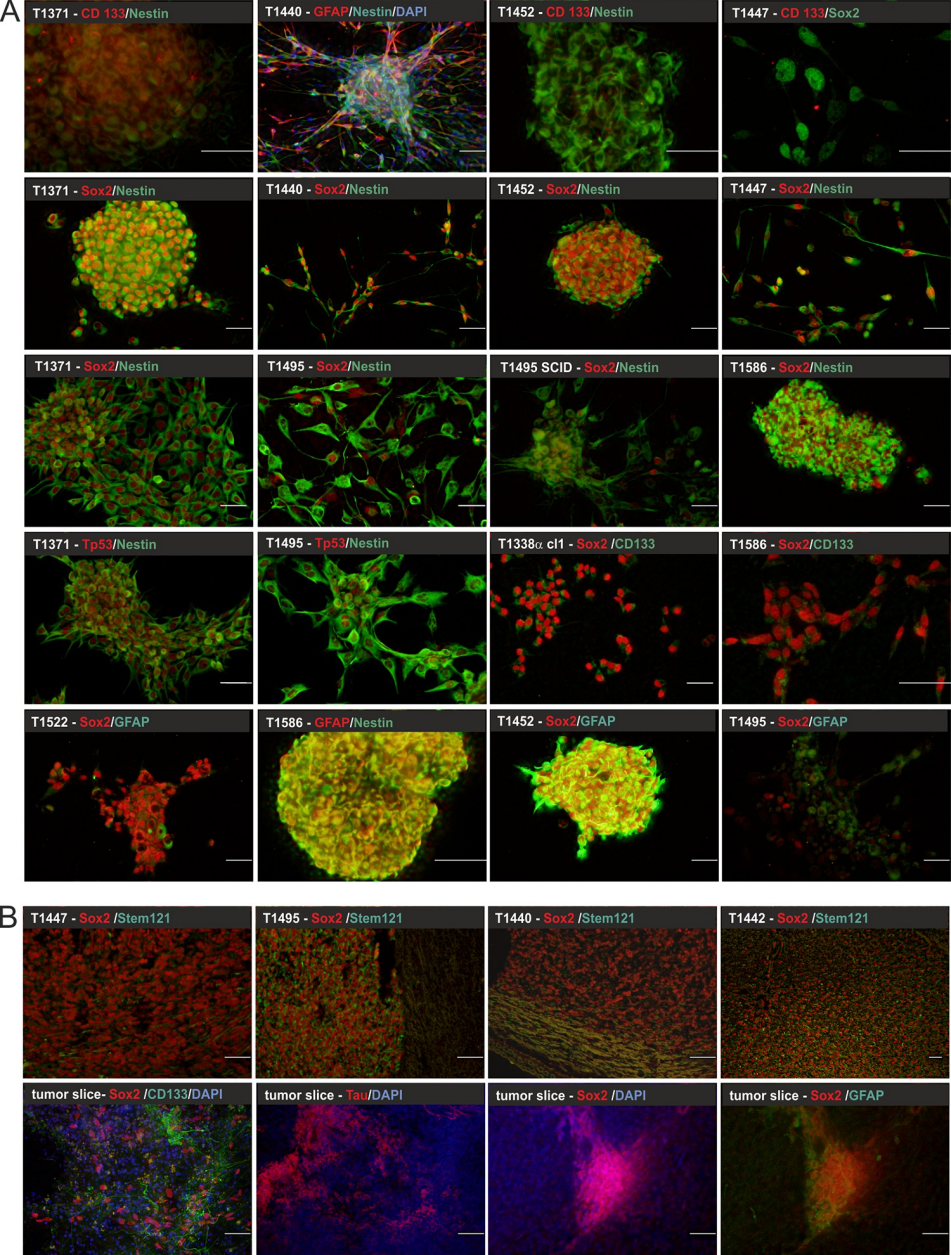
Immunofluorescence analysis of SLGC cultures and tumor specimen. (**A**) Immunocytochemistry; (**B**) Immunohistochemistry analyses of 200 μm slices from human GBM specimens (upper panel) and 4.5 μm slices from orthotopic tumors (lower panel).—The primary antibodies indicated in the figures were revealed with goat anti-mouse DyLight®488 (green) and goat anti-rabbit Cy3 (red), respectively. All microphotographs are z-stacks. For the sake of clarity, DAPI nuclear counterstain (blue) was omitted from several overlays.—Bars, 50 μm. GFAP, glial fibrillary acidic protein; CD133, Prominin-1; Sox2, SRY [sex determining region Y]-box transcription factor 2; Tp53, tumor suppressor p53; DAPI, 4′,6-diamidino-2-phenylindole.

**Fig 2:**
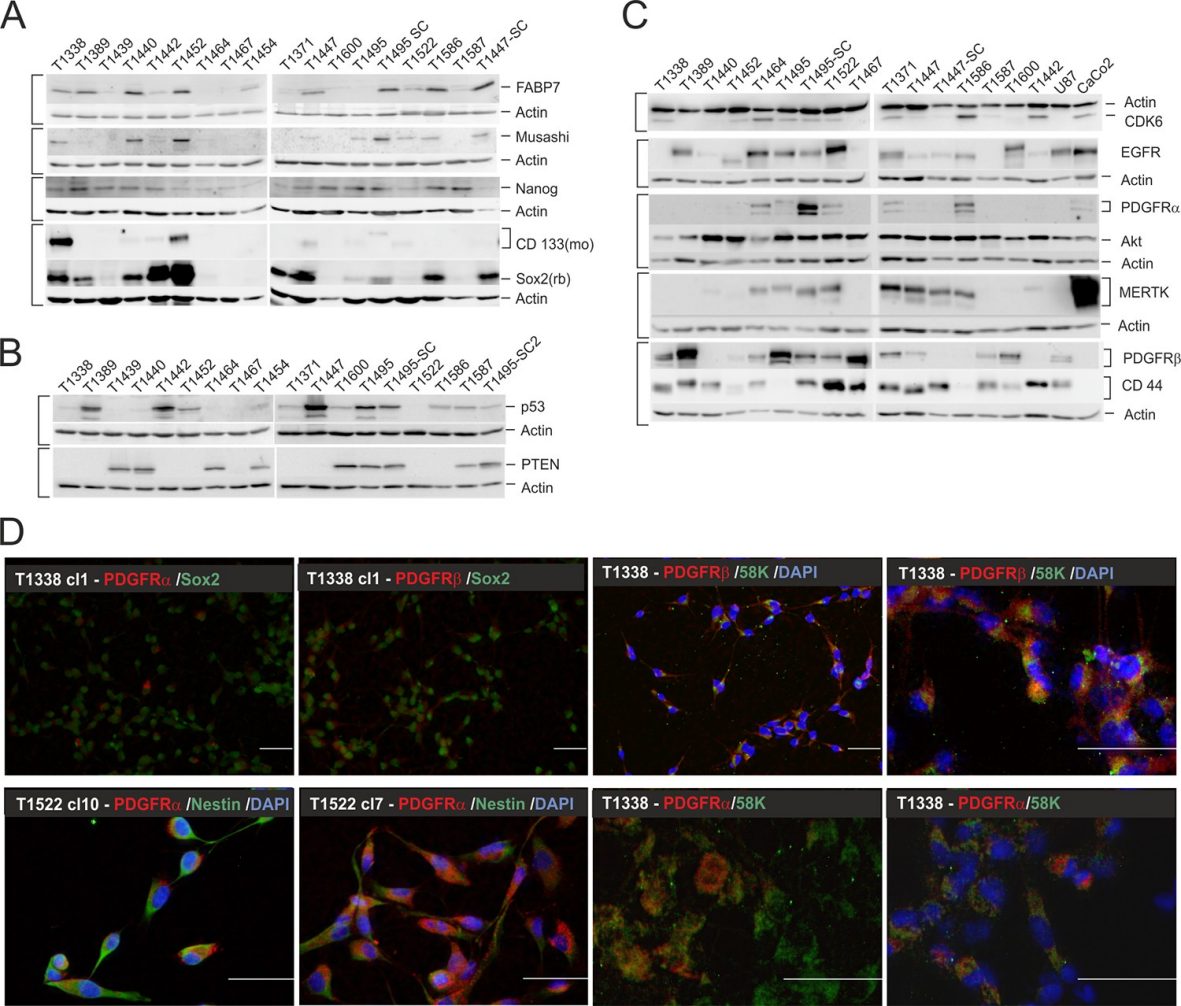
Expression of SLGC and GBM subtype markers. (**A-C**) Western blot analyses. The loading controls Actin (42 kDa) or GAPDH (glyceraldehyde-3 phosphate dehydrogenase; 36 kDa) are shown below the corresponding blots. Brackets indicate that the same nitrocellulose filter was used for the respective detections. (**D**) Immunofluorescence analysis of the expression of the platelet-derived growth factor receptors-1 and -2 (PDGFR-α, -β). The primary antibodies were revealed with goat anti-mouse DyLight®488 (green) and goat anti-rabbit Cy3 (red), respectively. K58 served as a marker for the Golgi apparatus.—Bars, 50 μm; DAPI, 4′,6-diamidino-2-phenylindole.–Akt, protein kinase B; CD133, Prominin-1; CD44, cell surface receptor that engages extracellular matrix components such as hyaluronan; CDK6, cyclin-dependent kinase 6; EGFR, epidermal growth factor receptor; FABP7, fatty acid binding protein 7; MERTK, tyrosine-protein kinase Mer; Musashi, RNA binding protein; Nanog, DNA binding homeobox transcription factor; PDGFR-α, -β, platelet-derived growth factor receptor α and β; PTEN, phosphatase and Tensin homolog; p53, tumor suppressor p53; Sox2, SRY-box transcription factor 2; SC, derived from orthotopic tumor.

SLGC cultures were established from 17 distinct human glioblastoma multiforme (GBM) and three gliosarcoma (GS) specimens, one of which (T1447) was a recurrent tumor. The cells were cultured in serum-free medium containing the growth factors EGF and bFGF, as serum would induce a more differentiated phenotype [36]. All SLGC cultures, including those derived from the gliosarcoma specimen, expressed the intermediate filament Nestin (examples in Fig 1A). Vimentin and CD15/SSEA-1 were observed in all cases, yet the distribution within the cells and on the cell surface, respectively, was very inhomogeneous (examples in S1 Fig). The pluripotency and transcription factor Sox2 and the glycoprotein CD133/Prominin-1 were observed in all SLGC cultures, but exhibited drastic differences in abundance and expression levels (Figs 1A and 2A). For example, in T1338/T1338 cl1, T1452 and T1586 cultures >90% of the cells showed an intensive Sox2 antibody stain, whereas only a few cells were CD133-positive, indicating that these cultures primarily contained type I cells (examples in Fig 1A). On the contrary, T1495 cultures established a pronounced cellular hierarchy with 10–30% type I and 70–90% more differentiated type II and III cells (Fig 1A). In GBM specimens Sox2-positive cells were observed in clusters, and co-expression of Sox2/CD133 was not evident (examples in Fig 1B, lower panel). In agreement with the observation that SLGCs may develop from neural stem-cell-like GFAP-positive or more differentiated GFAP-negative brain cells [37], we observed co-expression of high levels of GFAP and Sox2 in T1440, T1464, T1452 and T1586 (Fig 1A and Table 1). The neuronal microtubule-associated protein Tau, but not MAP2 was expressed by the SLGCs (examples in S1A Fig). Finally, the transcription factor Nanog, as well as the fatty acid binding protein FABP7 and the RNA binding protein Musashi were differentially expressed, with the levels of FABP7 and Musashi being more divergent (Fig 2A).

**Table 1:** Characteristics of SLGC lines and correlations.

Tumor type / Cell line	Meta-profile	PTEN	P53	growth	Sox2	IDH 1	DARPP-32	pref. Pax6	pref. CDKs	pref. RTKs	GFAP/stage	Tau	pref. α-/ β-Int
**ADAM9/Integrin**													
GBM–T1440	MP 1	-/+	WT	adh-rs	+—+++	WT	+	sh		-	stem	+	αv/β1
GBM–T1495	MP 1	+	GOF	s-adh	+—+++	WT	(-)	5a/sh	4,6	(E),Pα,Pβ,M	diff	+	αv/β1
T1495 SC	MP 1	+	GOF	sph	+—+++	WT	(-)	sh	4,6	(E),Pα,Pβ,M	diff	+	αv*/β1
**Jam-C/VE-Jam/Semaphorin–adhesion/migration**													
GBM–T1338	MP 2	-	WT§	s-adh	+++	WT	+	sh	6	(Pβ)	diff	+	αv/β1
GBM–T1464	MP 2	+	WT	s-adh	+—++	WT	(-)	sh	4,6	E,Pα,Pβ,M	stem	++	αv/β1,β**5**
**IG-H3/V-CAM-1/IL8 –immune cell adhesion**													
GBM–T1389	MP 3	-	GOF	s-adh	+—++	WT	(-)	5a/sh	4	Pβ	diff	+	αv/β1
GS*–T1447	MP 3	-	GOF	s-adh	+—++	WT	++	5a/6c/sh	6	(Pβ),M	diff	(+)	αv/β1
**Galectin-3b-BP/NCAM L1 –neural CAM/Galectin-BP**													
GBM–T1452	MP 4	-	mut	Sph	+++	WT	++	sh	4	-	stem	++	αv/β**2**
**Galectin-3/NCAM L1/ADAM17 –neural CAM/Galectin**													
GBM–T1586	MP 5	-	WT	sph	+++	WT	(-)	5a/sh	4,6	Pα,M	stem	++	αv/β**2**
GBM–T1587	MP 5	+	WT	sph	+—++	WT	(-)	5a/6c	4	-	diff	++	αv/β1
GS–T1371	MP 5	-	GOF	s-adh	+—+++	WT	++	(5a)/6c	4,6	E,Pβ,M	diff	(+)	αv/β1
GS–T1600	MP 5	+	GOF	sph	+—+++	WT	(-)	(sh)	4	E,Pβ	diff	++	α5/β1

GBM; glioblastoma multiforme; GS, gliosarcoma; GS*, recurrent gliosarcoma; PTEN, phosphatase and Tensin homolog; p53, tumor suppressor p53; WT, wild-type; WT§, heterozygous p53 mutant in a subpopulation of cells; mut, mutant; LOF, known loss of function mutant (N239D); GOF, known gain of function mutants (R175H, R273H, R248W); s-adh, semi-adherent; adh-rs, adherent rosettes; sph, spheres; IDH2, NADP-dependent mitochondrial isocitrate dehydrogenase CD44, receptor for e.g. hyaluronan; Pax6: 6c canonical isoform (422 AA; 46.7 kDa); 5a, isoform 5a (436 AA, 48.2 kDa); sh, shorter isoforms (33–35 kDa; 25–32 kDa); CDK4/6, high levels of cyclin-dependent kinases 4 and 6, respectively, are indicated; RTK, high levels of receptor tyrosine kinases are indicated; E, epidermal growth factor receptor; Pα, Pβ, platelet-derived growth factor receptors α and β; M, Met; GFAP, glial fibrillary protein; diff, neural differentiation; stem; stem-like state; Tau, microtubule-associated protein; pref. α−/β-Int, preferred combination of alpha and beta integrins; αv*, no clear preference for any alpha integrin; DARRP32, phosphatase 1 inhibitor; AADC, aromatic amino acid decarboxylase; isoforms: L (53.9 kDa, canonical), M (45.6 and 44.2 kDa, not characterized), S (37.1 kDa, in e.g. kidney; here observed in CaCo2 cells); symbols (-), (+), +, ++, +++, indicate increasing expression levels from—or (-) absent or below detection level/, (+) detectable/low, till high expression. E, Pα, Pβ, M

With regard to factors integrated in the new WHO guidelines for GBM diagnosis [2–4] we analyzed the IDH1, PTEN and p53 status. Only eight out of 20 SLGC lines expressed PTEN, even though RT-PCR revealed *pten* transcripts in all cell lines (Fig 2B; for examples, see S1 Appendix). T1440 and two other SLGC lines displayed a mixed PTEN phenotype, so we applied limited dilution assays. We isolated PTEN-positive and PTEN-negative T1440 subclones, and clones with a mixed PTEN phenotype [S1 Table], indicating intratumor heterogeneity. Those SLGC lines, which we planned to use in prospective treatment experiments, were analyzed by Sanger sequencing to determine their p53 and IDH-1 status. Except for one silent mutation (T1389), all IDH-1 sequences matched the wildtype [S1 Table]. With respect to p53, eight out of 16 SLGCs were homozygous for the p53 wildtype, seven were homozygous for p53 mutations, and one (T1338) was heterozygous for a p53 mutation in a subpopulation of cells. The identified p53 mutations included (i) well-known gain-of-function (GOF) mutations, such as p53^R175H^ (T1389, T1371, T1600), p53^R273H^ (T1495), p53^R248W^ (T1447), (ii) the loss-of-function mutation p53^N239D^ (T1442) and (iii) a so far unknown splice mutant (T1452, Table 1 and S1 Table). By means of limited dilution assays, we isolated ten T1338 subclones. Sequencing revealed that only half of the T1338 clones were heterozygous for the p53 mutation [S1 Table], while all others (e.g. T1338 cl1) carried the p53^WT^ on both alleles. Expectedly, the presence of the p53 mutations was reflected by strong p53 signals in immunocytological stains and western blot analyzes (examples in Figs 1A and 2B).

All SLGC lines, except for T1338 (and T1338 subclones), generated orthotopic tumors in mice within 12 weeks after xenotransplantation, which were revealed by HE staining, immunohistochemistry, and, in some cases, accessorily verified by coronal T2-weighted MRI scan (examples in S1B and S1C Fig). T1338, as well as the T1338-p53^WT^ and T1338-p53^WT/mut^ subclones, did not generate orthotopic tumors within 6 months, though Sox2-positive human cells could still be detected in the respective mouse brains (examples in S1D Fig). The largest orthotopic tumors were derived from T1495 and T1447, both of which displayed a pronounced cellular hierarchy in cell cultures, whereas SLGC lines consisting of mainly type I cells (high Sox2 expression in >90% of the cells and at best weak CD133 signals) generated smaller (e.g., T1440,T1452) or no orthotopic tumors (e.g. T1338 cl1). Notably, all orthotopic tumors consisted of human cells (revealed by αStem 121) expressing high levels of Sox2 (examples in Fig 1B).

### Metaprofiles of SLGCs

We selected eight of the GBM-derived and all GS-derived SLGC lines for the analysis of the cell surface protein signature. In addition, we included the T1495-SC line, which was derived from a macroscopically visible orthotopic tumor that developed from xenotransplanted T1495 cells. The criteria for selection were (i) absence or presence of Sox2/GFAP co-expression, (ii) absence or presence of the type I-III cellular hierarchy, and (iii) growth behavior. The majority of the SLGC lines displayed semi-adherent growth, i.e., the cells formed aggregates with the ability to loosely adhere to the growth matrix. Exclusive spherical growth was observed for T1495-SC, T1452, T1586 and T1587, whereas T1440 cells formed an adherent rosette-like structure (Fig 1A) reminiscent of self-renewing human ES cell-derived neural stem cell with the potential of neuronal differentiation [38]. For sake of comparison, the established GBM-derived cell line U87 (Sox2-negative) and the colon carcinoma cell line CaCo2 were included (CaCo2 cells are CD133-positive; S1A Fig).

**Fig 3:**
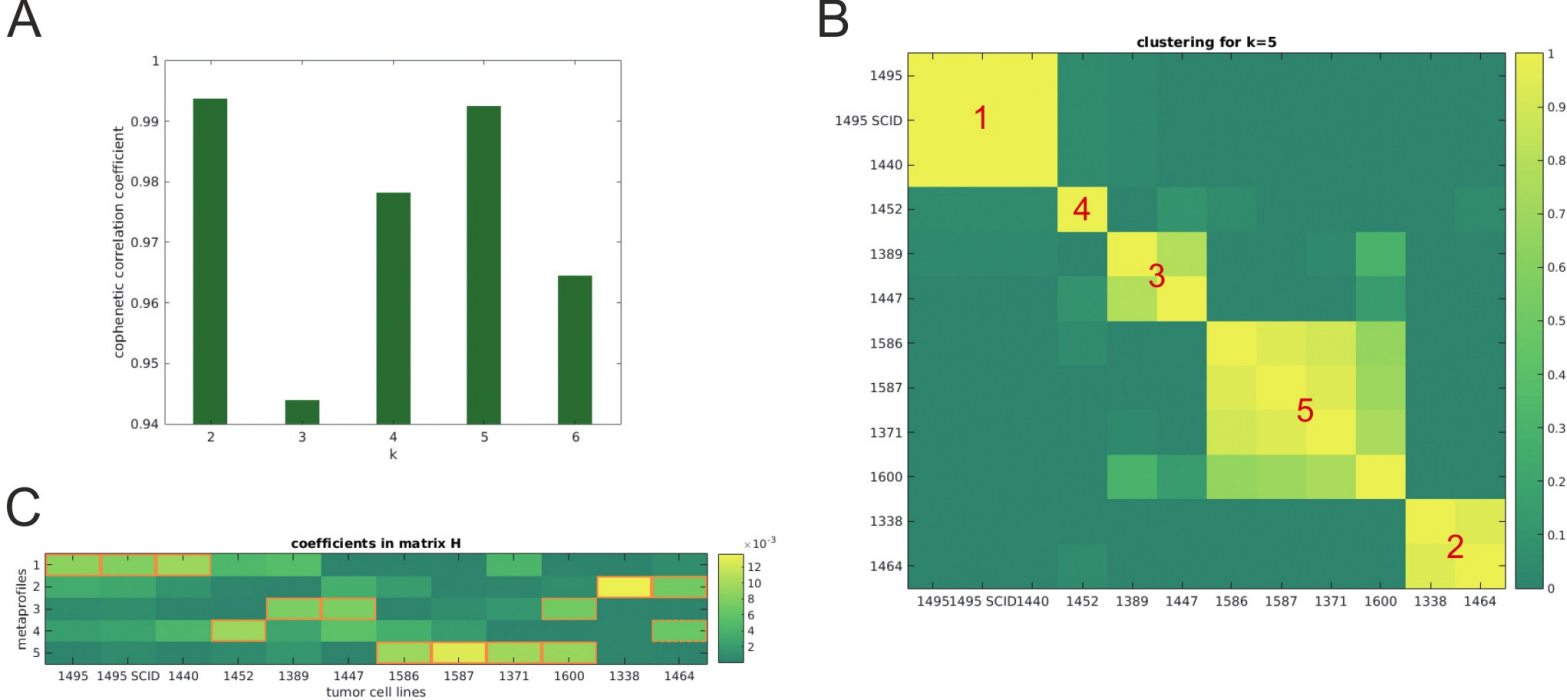
Cophenetic Correlation Coefficients (CCC). **(A)** The cophenetic correlation coefficient (CCC) is calculated for 100 runs with k = 2,..,6. The CCC reaches two maxima, one for k = 2 and one for k = 5. **(B)** Cluster plot illustrating similarities between the different SLGC lines for k = 5. This subfigure shows the pairwise distance between the different SLGC lines. The largest coefficients in matrix H can be used to assign the individual clusters to their corresponding metaprofile (MP, indicated by numbers). Although the clusters can be reduced to one MP, this is not a sufficient criterion for the fact that the MP conversely only characterizes these specific cell lines. For cluster 4 (MP4), e.g., there is only line 1452 with a maximum expression for this profile, even though for several other lines MP 4 plays a role. Cell line T1600, with two (almost equally) sized coefficients for MP 3 and 5, is located somewhere between their corresponding clusters. **(C)** Column-wise representation of the coefficients characterizing the SLGC lines. The 12 SLGC lines are indicated on the X-axis, and the five coefficients from matrix H are depicted on the y-axis and are color coded. The brightness indicates the coefficients value, a bright yellow for the largest coefficient, a dark green for the lowest. These coefficients linearly combine with the 5 metaprofiles to the original expression pattern.

The protein expression data obtained from a commercial *Non-Hematopoietic* cell surface receptor array were applied to a cophenetic correlation coefficient calculation (CCC). The CCC reached two maxima, one for k = 2 and one for k = 5 (Fig 3A). The similarities between the different SLGC lines are illustrated in the cluster plot in Fig 3B, which additionally assigns the SLGC lines to the five metaprofiles (MP1-MP5). The column-wise representation of the coefficients characterizing the SLGC lines in Fig 3C illustrates, e.g., the similarities between T1495 and T1440 and T1495-SC (MP1). Whereas T1452 is the only SLGC line characterized by MP4, two SLGC lines are assigned to MP3 and MP5 each, and four to MP5. Notably, the three GS-derived lines were assigned to distinct MPs, in which both primary GS lines T1371 and T1600 were best characterized by MP5 and the SLGC line from the recurrent GS (T1447) by MP3. The five metaprofiles were validated by matrix reconstruction (S2 Fig).

**Fig 4:**
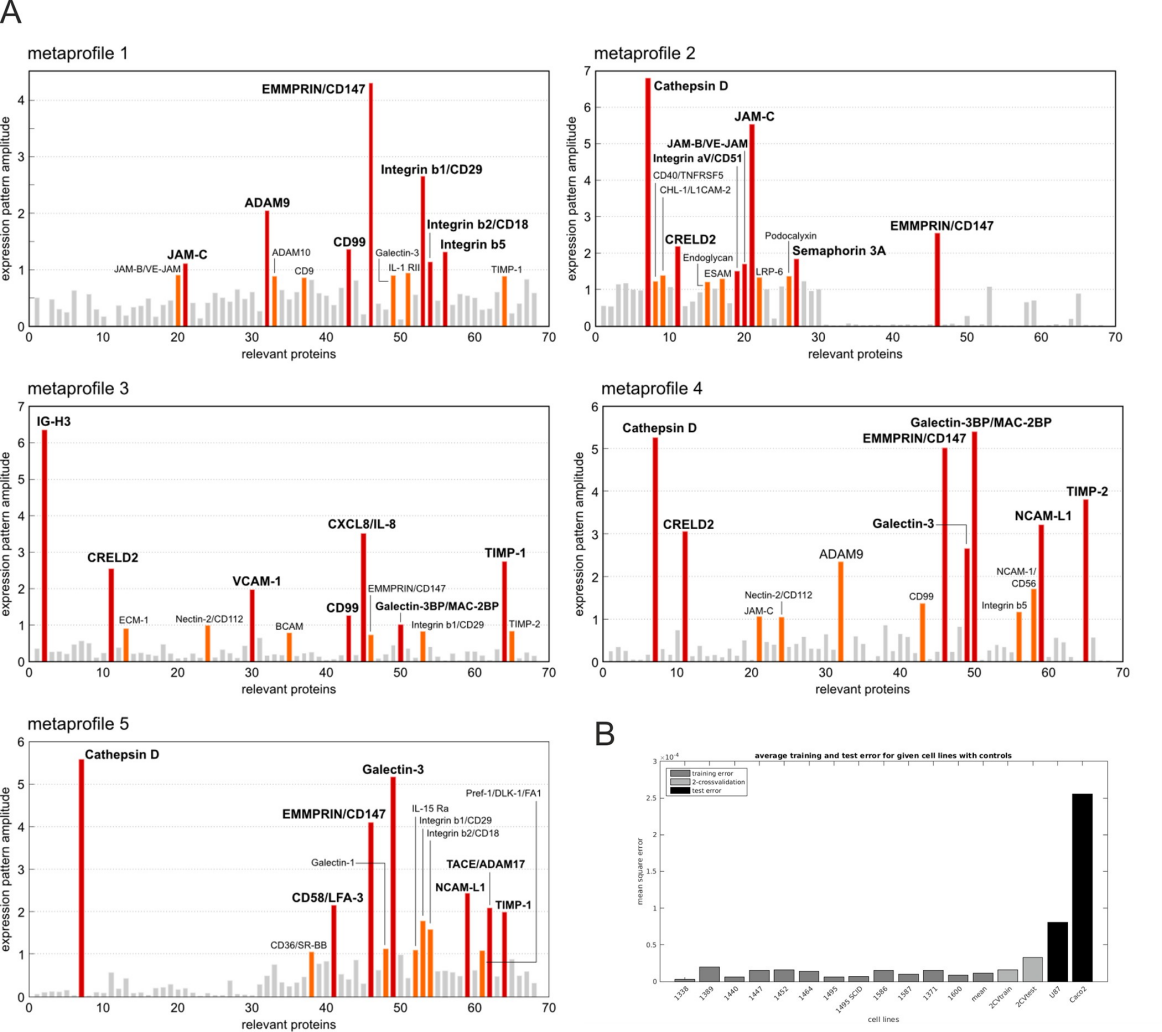
Assignment of proteins to metaprofiles and crossvalidation. (**A**) Protein-specific characterization of the metaprofiles (MPs). Each of the five plots decomposes each metaprofile along its signature proteins. Proteins that showed no significance (<80% percentile) for at least one of the MPs were discarded. The remaining 68 proteins are indicated in color codes, highlighting the top 10% for each MP in orange, and the top 5% in red with a bold label. The proteins highly specific for the MPs are: ADAM 9, disintegrin and metalloprotease domain 9; B-CAM, basal cell adhesion molecules; Cathepsin D; acid protease; CD 99, antigen involved in cell adhesion processes; CD58/LFA-3, lymphocyte function-associated antigen 3; CHL-1/L1CAM-2, neural adhesion molecule L1-like protein; CRELD2, Cysteine-rich with EGF-like domains 2; Galectin-3BP/MAC-2BP, galactose-specific lectin; EMMPRIN/CD147 (Basigin), targets monocarboxylate transporters and is probably a glycan receptor; CXCL8/IL8, chemotactic factor with high affinity to the receptors CXCR1 and 2; ESAM, endothelial elective adhesion molecule; IL-1 RII, interleukin-1 receptor type 2; Integrins β1, β2, β5, αv; JAM B and C, junctional adhesion molecules B and C; Semaphorin 3A, protein promoting e.g. migration, dendrite outgrowth but also immune responses; IG-H3, transforming growth factor (TGF) β-induced protein; LRP-6, low-density lipoprotein receptor-related protein 6; NCAM-L1, neural cell adhesion molecule L1; VCAM, Vascular cell adhesion protein 1; TIMP1 and 2, tissue inhibitor of metalloproteinases 1 and 2; LFA, lymphocyte-associated antigen-3; TACE/ADAM 17, disintegrin and metalloprotease domain 17; CD40/TNFRSF5, tumor necrosis factor receptor superfamily member 5. For additional abbreviations and explanations, see the text. (**B**) Comparison of the mean squared reconstruction error of the original data (SLGC lines) with two non-SLGC lines U87 (established glioblastoma cell line without stemness features) and CaCo2 (established colon cancer cell lines with >95% CD133-positive cells). A two-fold cross-validation leads to slightly higher training and test errors than the average training error on all expression data. The test on the non-SLGCs shows that the learned features are rather SLGC-specific.

**Fig 5:**
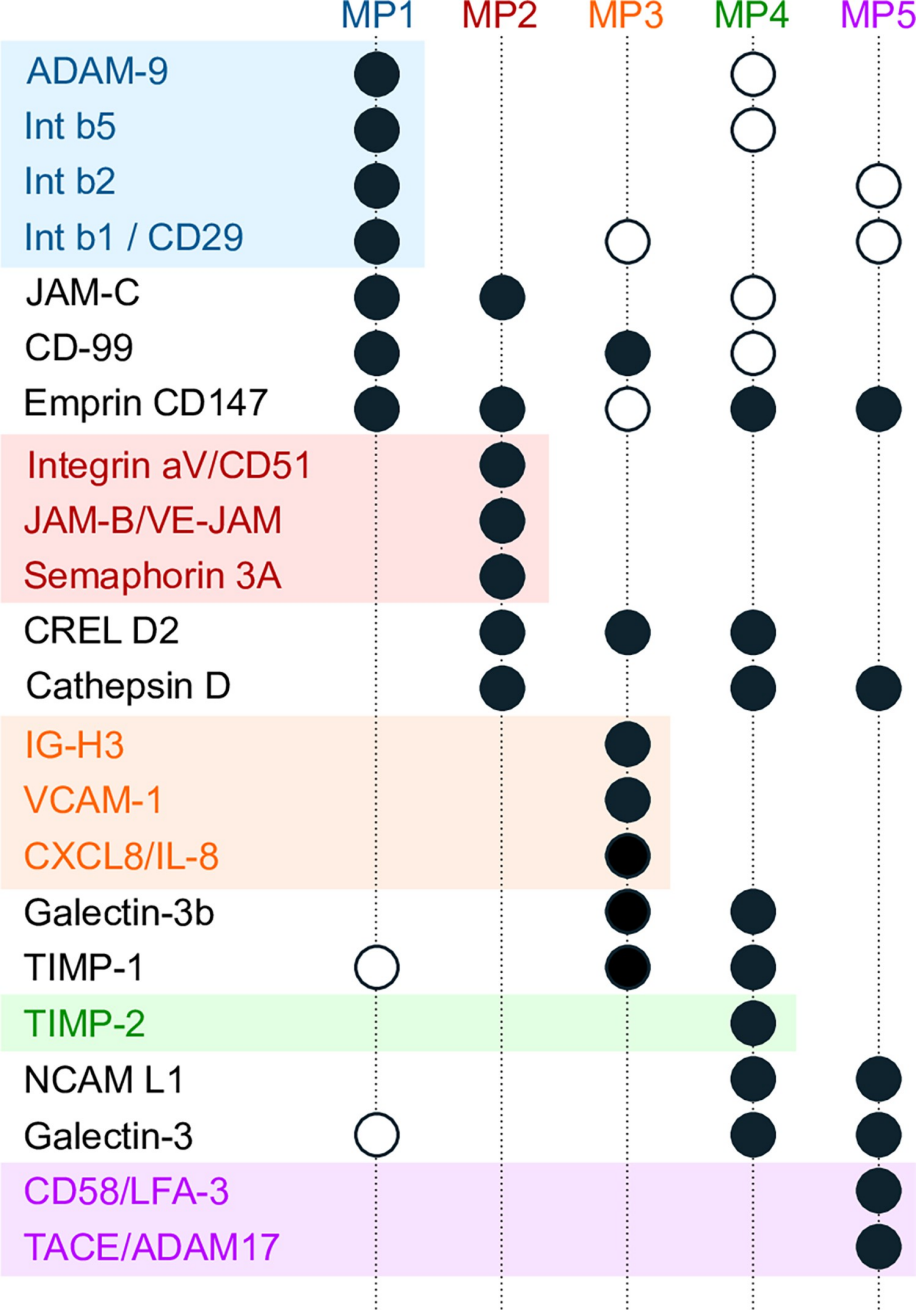
Proteins characterizing the metaprofiles. For all proteins that are significant (>95% percentile) for at least one metaprofile (MP1-5), their decoding along all MPs is shown. Solid dots indicate those above 95%; the shallow dots are those above 90% (see also Fig 4A). Furthermore, the proteins are sorted to match their principal MPs. All proteins that can be assigned to exactly one MP are clustered, and these clusters are color coded (MP1: blue, MP2: red, MP3: orange, MP4: green, and MP5: violet). As no unique protein could be linked to MP1, we used for clustering those that have expression above 90% but below 95% for the other MPs. All other proteins are assigned to a minimum of two metaprofiles (e.g., Galectin 3, NCAM L1) or all metaprofiles (CD147).—For abbreviations, see the legend to Fig 3.

Except for MP3, the extracellular matrix metalloproteinase inducer EMMPRIN/CD147 [39] and membrane-associated proteases, such as Cathepsin D [40], ADAM9 or ADAM17 dominated the metaprofiles (Figs 4A and 5). With respect to the ADAM (A disintegrin and metalloproteases) proteases [41], ADAM9 was the dominant protease in MP1 and MP4, whereas ADAM17 characterized MP5 (Fig 4). Beta-integrins, in particular Intβ1, Intβ2 and Intβ3, were among the factors characteristic for MP1 and MP3-MP5, but not MP2; instead, MP2 was characterized by the alpha-integrin Intαv (Figs 4A and 5). The neural cell surface protein NCAM-L1, which seems associated with poor prognosis in glioma [42], characterized MP4 and MP5. Semaphorin 3A, which has been shown to influence innervation and sympathic nerve outgrowth [43], was most specific to MP2. With the exception of Intβ1, EMMPRIN/CD147 and CD99 (a protein involved in cell adhesion, migration, death, and differentiation as well as intracellular protein, trafficking, endocytosis, and exocytosis [44]) the metaprofile MP3 appeared quite distinct from the others. MP3, which describes best the SLGC lines T1389 and T1447, is characterized by Ig-H3 (a RGD-containing collagen-associated protein, promoting cell adhesion [45]), VCAM-1 (vascular cell adhesion molecule; a cytokine-induced adhesion molecule upregulated by EGFR in GBMs [46]) and CXCL8/IL8 (a chemotactic factor attracting neutrophils, basophils, and T-cells [47]). A factor shared between MP2, MP3, and MP4, which has not been mentioned yet, is CRELD2 (Cysteine-rich with EGF-like domain protein 2; Fig 5), a factor regulated by the ROCK-PERK-ATF4 axis with the potential to promote tumor progression in breast cancer [48]. Additional factors characterizing the metaprofiles MP2 (T1338, T1464), MP4 (T1452) and MP5 (T1371, T1600, T1586, T1587) are the lectins Galectins 3 and 3b (Figs 4A and 5), proteins with (i) high affinities for β-galactosides, (ii) potential roles in neuroprotection and neuroinflammation, and (iii) a significance for processes involved in neurodegenerative diseases, such as Alzheimer’s or Parkinson’s disease [49]. Taken together the metaprofiles MP1, MP2, MP4, and MP5 suggest a signature which is characterized by EMMPRIN/CD147 and several proteases, which proposes a high cell motility. In addition, the factor NCAM L1 suggests a stronger neural character of the cell lines assigned to MP4 and MP5. The factors specific for MP3 (T1389, T1447) and the additional importance of TIMPs (metallo-peptidase inhibitors) for this metaprofile could suggest that cells with the MP3 signature are less motile but rather recruit non-tumorous cells and establish cell-cell interactions.

When we applied the cophenetic correlation coefficient calculation (CCC) to array data obtained for U87 and CaCo2, it became evident that these cell lines are not characterized by any of the five SLGC metaprofiles (Fig 4B). This is not surprising for the CaCo2 line, as these CD133-positive cells are derived from a human colon cancer. The U87 cell line, however, is closer to the SLGC lines than to the CaCo2 line, but its metaprofile is still distinct from the SLGC metaprofiles (Fig 4B).

### GBM- and SLGC-associated expression pattern

**Fig 6:**
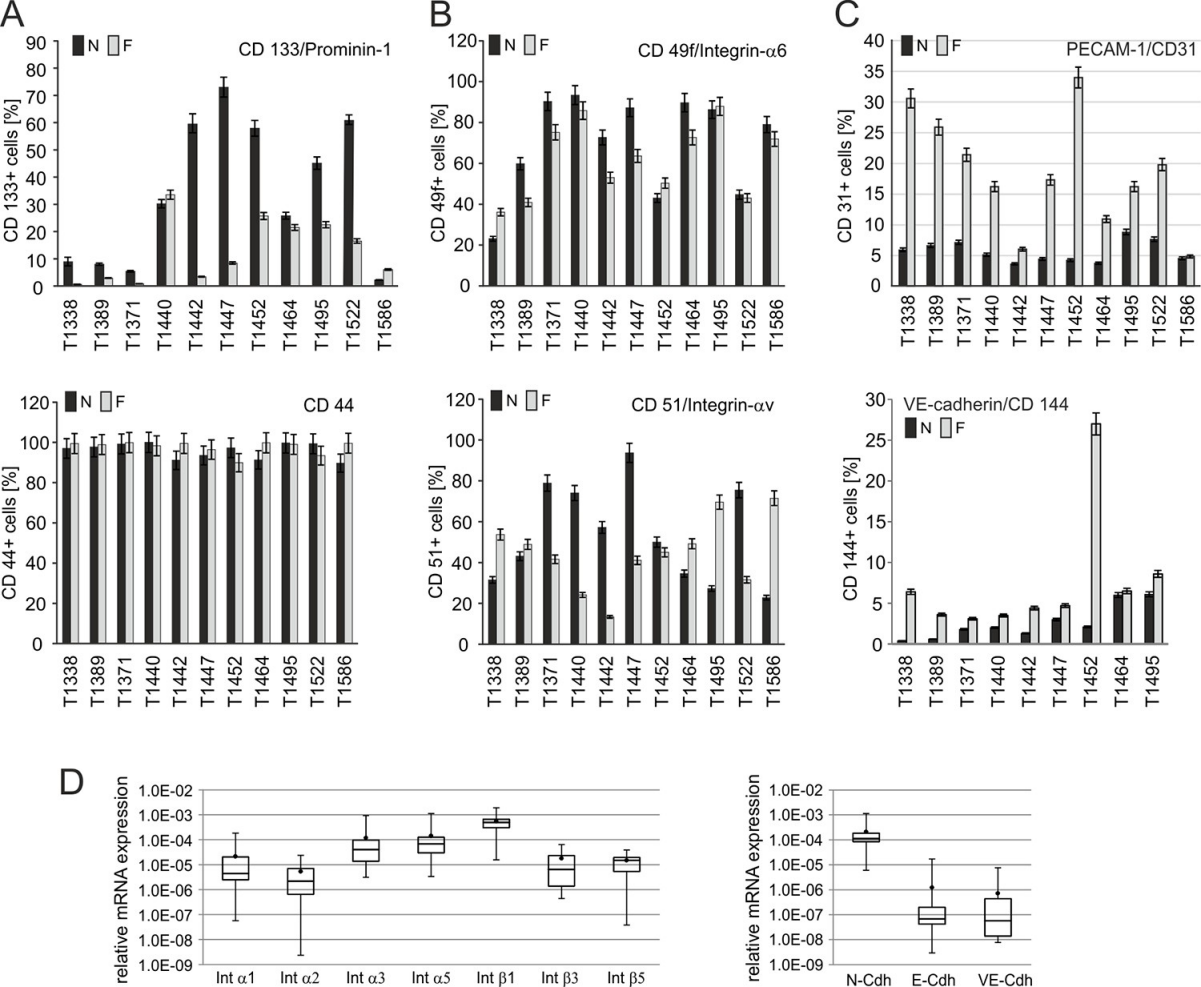
Expression of cell surface proteins. (**A, B, C**) Flow cytometry analyses of SLGCs expanded in serum-free (N) and serum-containing (F) medium. Fluorochrome-coupled antibodies directed against CD133/Prominin-1, CD44, integrin α6/CD49f, and integrin αv/CD51, as well as the endothelial CAM (cell adhesion molecule) PECAM/CD31 or the endothelial cadherin VE-cadherin/CD144 were used. A minimum of 10,000 cells was analysed. Assays with corresponding N- and F cultures were done in parallel. Bars represent mean values, whiskers represent the variation between technical replicates. (**D**) Box plots summarizing the real-time PCR (qRT-PCR) analyses using primers directed against integrins (Int) or cadherins (Cdh). The plots indicate the mean values calculated from the qRT-PCR data of 12 distinct SLGC lines, in which each SLGC line was analyzed in three individual reactions. The standard deviations are indicated by the upper and lower borders of the boxes, and the median is symbolized by the central line. The range of minimal and maximal values is represented by the whiskers. The qRT-PCR values were normalized against the reference genes *gapdh*, *ubiquitin ligase*, and 18 s *r-rna* prior to calculation of the Box plots.

The cophenetic correlation coefficient calculation (CCC) suggested a key role of the integrins Int-β1, Int-β2, Int-β3, and Intαv. Altogether, the proteome array provided data for five alpha-integrins (Int-α3, -α5, -α6, -α9, and Intαv) and six beta-integrins (Int-β1, -β2, -β3, -β4, -β5, and Intβ6). Even for those cell lines that were not assigned to a metaprofile characterized by integrins, the relative expression of Intαv and Intβ1 was the highest (S3A and S3B Fig). In order to further validate this observation, we used the SLGC lines characterized by the proteome assay and eight additional SLGC lines, for flow cytometry, qRT-PCR and Western blot analysis. These experiments proved the preferential expression of Int-β1 and a higher variability of the expression of Int-β2 and Int-β5 (Fig 6B, 6D and S3C, S3E Fig, and examples in S1 Appendix). Contrary to expectations based on previous reports [50, 51], we observed lower levels of protein and mRNA for Int-α6 than for Int-αv. In flow cytometry analyzes the relative numbers of Int-α6- and Int-αv-positive cells varied between 18% and 99%. When SLGCs from the same passage were grown under expansion (N-medium) and differentiation (F-medium) conditions, respectively, it became evident, that differentiation had very little effect on Int-α6 expression (Fig 6B and S3 Fig), predicting that Int-α6 is not a marker for the stemness state. A marked, but diverse responsiveness to differentiation was discovered for Int-αv expression (Fig 6B and S3 Fig). For example, the Int-αv levels (number of positive cells, mRNA levels) were increased in some SLGC lines (T1338, T1495, T1586) and decreased in others (T1371, T1440, T1442). Notably, the numbers of Int-αv- and Int-α6-positive cells also varied between biological replicates, though mostly within minor ranges (examples in S1 Appendix). With respect to the type II cell marker CD133, we observed a marked reduction of the number of positive cells when grown under differentiation conditions for most SLGC lines but not for T1440, T1464 or T1586 (Fig 6A).

Considering the observation that SLGCs might transdifferentiate to an endothelial-cell-like phenotype [52], we analysed the numbers of PECAM-1/CD31- (endothelial cell adhesion molecule) and VE-(vascular endothelial)-cadherin/CD144-positive cells by flow cytometry in three distinct biological replicates. Except for T1442 and T1586, the FCS-mediated upregulation of CD31+ cells was very pronounced, and CD144 upregulation was efficient in all cases except for T1464 and T1495 (Fig 6C). The expression of VE-cadherin mRNA was already revealed by qRT-PCR in SLGCs grown in N-medium (Fig 6D). In this regard, the expression of VE-Cadherin mRNA was relatively similar to that of the E-cadherin mRNA, whereas the expression N-cadherin mRNA was much higher (Fig 6D). That N-Cadherin dominated over E- and VE-cadherin was additionally supported by the proteome array and Western blots (S4A–S4C Fig, and examples in S1 Appendix).

The proteome array indicated the presence of all four types of EGF receptors (HER1-4) (S5A Fig). As the EGFR (HER1) has been (i) proposed as a GBM subtype maker [16, 20, 21], (ii) the EGFR is typically expressed in activated adult neural stem cells and progenitors [53], and (iii) EGF is one of the two growth factors present in the serum-free N-medium used for SLGC cultures [22, 23] we investigated the role of the EGFR in more detail. First, we observed that all SLCGs expressed *egfr* mRNA and EGFR protein, though at distinct levels (Fig 2C and S5B Fig; and examples in S1 Appendix). A very strong Western blot signal, which could be interpreted as a sign of EGF receptor amplification, was only observed in extracts from T1464 and disappeared over the passages (S5B Fig, and examples in S1 Appendix). EGFRs of abnormal size were, e.g., revealed for T1452 (truncation) and T1600 (increased length) by Western Blots (e.g., Fig 2A). Second, all SLGC lines expressed several EGFR/HER-1 ligands (mRNA and protein; examples in S5A, S6A and S6D Figs), suggesting that SLGCs might stimulate their growth by autocrine mechanisms. This hypothesis was supported by experiments indicating that removal of EGF from the culture medium resulted in a partial growth reduction. In addition, bFGF mRNA was also expressed by the SLGC lines, and bFGF depletion provoked growth reduction in the majority of SLGC lines (examples in S6B and S6C Fig). Growth factor ELISA revealed that the stability of exogenously added EGF is much higher than that of bFGF, and that the amounts of EGF secreted by the SLGCs reached 25 pg (T1371) or even 100 pg (T1447), whereas the secretion of bFGF was barely detectable (examples in S6D Fig). Third, the same SLGC lines often expressed EGFR in the presence of PDGFRα or –β, or the MERTK (MER proto-oncogene, tyrosine kinase). In particular, MERTK, which is expressed in distinct types of cancer, including glioblastoma [54, 55], showed high expression in two out of the three GS-derived SLGC lines and three GBM-derived lines (Fig 2C and Table 1). High levels of the PDGFRα were observed for T1495-SC, followed by T1464, T1586, T1522, and, e.g., T1495, all of which additionally displayed intermediate to high levels of EGFR and MERTK (Fig 2C and S5B Fig). The PDGFRα blots revealed reproducibly two truncated forms of the receptor, with a molecular weight of approx. 80–90 kDa and 30–35 kDa, in which the truncated forms were often more abundant than the full-length protein (S5B Fig). The immunocytological stains indicated a heterogeneous dispersion of PDGFRα in the plasma membrane and in the cytoplasm of cells with high PDGFRα levels. SLGCs with low PDGFRα levels, however, displayed PDGFRα signals in dividing cells in the cytoplasm (examples in Fig 2D). Co-stains with a K58 antibody (Fig 2D) indicated a cytoplasmic PDGFRα location outside the Golgi apparatus, suggesting that the truncated receptors are located in distinct cytoplasmic compartments. Fourth, the proteome array indicated the expression of VEGF (vascular endothelial growth factor) receptor-1 and -2 in all SLGC lines, with a high abundance of VEGFR-2 in the cases of all GS-derived SLGC lines and three GBM-derived SLGC lines (T1587, T1586, and T1338; S7B Fig).

**Fig 7:**
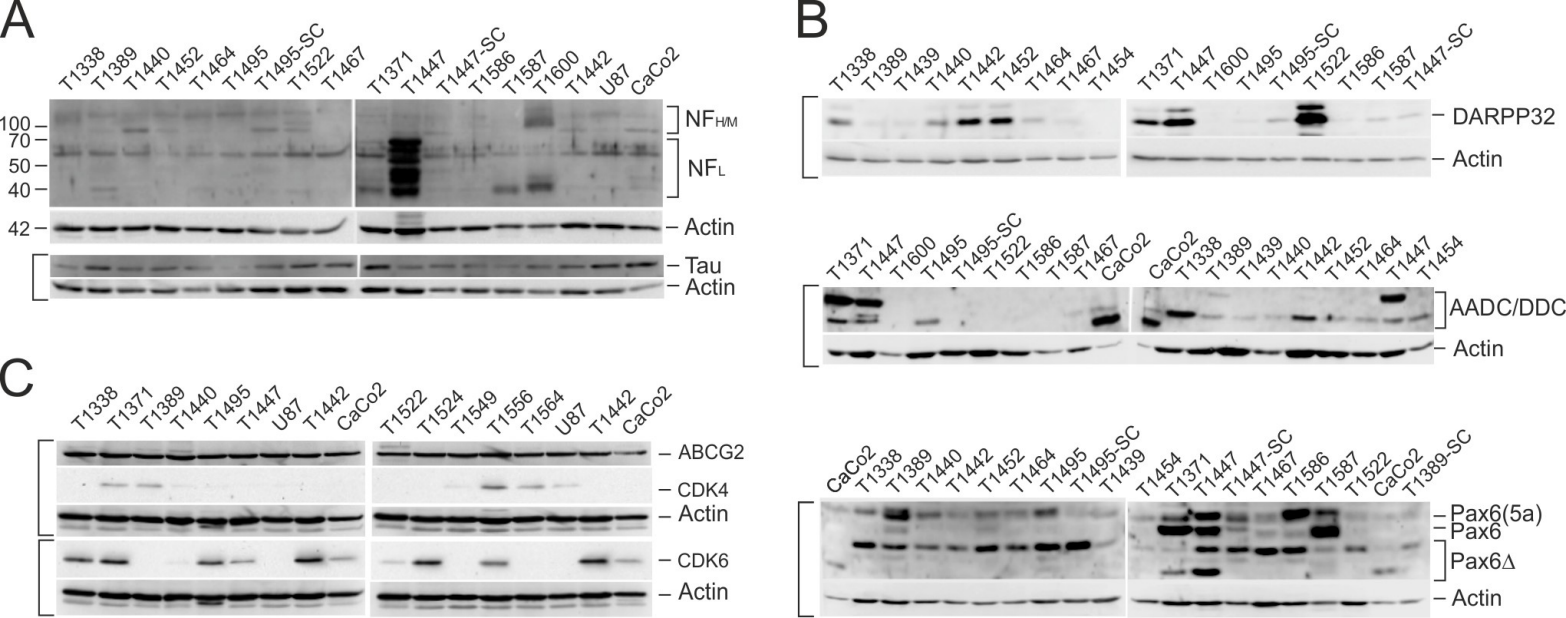
Expression of neuronal proteins, cell cycle regulators and ABCG2. (**A-C**) Western blot analyzes: The loading control Actin is shown below the corresponding blots. Brackets indicate that the same nitrocellulose filter was used for the respective detections.—AADC/DDC, aromatic L-amino acid decarboxylase/DOPA decarboxylase [isoforms due to alternative transcript splicing: 53.9 kDa; 40–48 kDa; 37.1 kDa]; ABCG2, ATP-binding cassette subfamily G2 member 2; CDK4 and 6, cyclin-dependent kinases 4 and 6; DARPP32, 32 kDa dopamine and cyclic adenosine 3’,5’-monophosphate regulated phosphoprotein; NF, neurofilaments H, M and L [heavy polypeptide, 220 kDa; medium polypeptide, 160 kDa; light polypeptide, 68 kDa]; Pax 6, paired box protein 6 (isoform 1: 46.7 kDa; isoform 5a: 48.2 kDa; additional isoforms: molecular weight below 40 kDa); Tau, microtubule-associated protein Tau. SC, derived from orthotopic tumor.

Next, we focused on additional factors associated with neural identity. Neurofilament expression, which had been associated with GBM subtypes [16] was very low except for the GS-derived line T1447 (Fig 7A). A high co-expression of the DOPA-Decarboxylase (AADC/DDC, aromatic amino acid decarboxylase) and DARPP32 (32 kDa dopamine and cyclic adenosine 3’,5’-monophosphate regulated phosphoprotein) was observed in T1442 and T1338, and in particular in the GS-derived lines T1371 and T1447, whereas T1522 displayed high DARPP32 levels in the absence of the AADC/DDC (Fig 7B). Though both the AADC/DDC and DARPP32 proteins are not exclusively expressed in neurally programmed cells [56, 57], and references therein, their high co-expression levels could suggest that these SLGC lines originate from regions with neurogenic activities. It is striking that the AADC/DDC-positive GS-derived cell lines express an isoform of a larger molecular weight and that a third, smaller isoform was observed in the CaCo2 cell line (Fig 7B). An isoform-related expression pattern was also observed for the transcription factor Pax6, which is important in various developmental processes in the central nervous system, including the modulation of Sox2 expression in neural progenitors ([58] and references therein). As for the AADC/DDC, the Pax6 isoform expression pattern showed similarities between T1371 and T1447, whereas the GBM-derived SLGC lines primarily expressed the isoform Pax6-5a in addition to truncated Pax6 isoforms (Fig 7B).

Aiming at the identification of correlations between marker expression and SLGC identity, we additionally analyzed the expression of the cell cycle regulators CDK4 and CDK6, the ABC transporter ABCG2 [59], the hyaluronan receptor CD44 [60], MGMT (O-6-Methylguanine-DNA Methyltransferase), IDH1 and IDH2, the kinase Akt, the type III cell marker DLX2 [28], as well as Notch and the Notch ligand Jagged [61], the BMP receptor IB/Alk6 [62], the co-receptor LRP-6, the glycoprotein CD58/LFA-3, and compared the relative expression of proteases and protease inhibitors (Fig 7, S5C, S5E, S7C and S8A–S8C Figs, and examples in S1 Appendix). These data entered into the calculation of the Pearson correlation coefficients, and only a few important aspects will be described here. The ABC transporter ABCG2 has been assigned several roles in GBMs, including the regulation of self-renewal and stem cell marker expression [59]. We observed nearly invariable expression of ABCG2 in Western blots and immucytochemistry in SLGC lines (Fig 7C and S1A Fig), which appeared independent of degree of stemness and differentiation. The hyaluronan receptor CD44, which has also been proposed as a SLGC and GBM subtype marker with significance in tumor progression [60] showed varying expression levels in Western blots, in which high CD44 expression was observed for both SLGC cultures with high and low Sox2 levels (Fig 2C). Moreover, the number of CD44+ cells varied between 95–99% in undifferentiated and differentiated SLGC cultures und appeared unrelated to the numbers of CD133-positive cells as well as CD133 and Sox2 levels (Figs 1A, 2A, 6A, S1A and S3D Figs) suggesting that it is inappropriate to consider CD44 a SLGC marker. In this context, it is important to note that (i) the numbers of CD133+ but not of CD44+ cells varied during continuous passaging, (ii) the numbers of CD44+ were constantly above 95% and (iii) the numbers of CD133+ cells were already very heterogeneous during early passages (Fig 6A and S3D Fig, and examples in S1 Appendix). IDH1 displayed a nearly invariantly high expression in all SLGC lines, whereas the expression of IDH2 appeared highly variable in Western blots (S5E Fig). Finally, whereas the relative expression of the ADAM proteases and TIMPs varied, Cathepsin D expression was generally high (S8A–S8C Fig).

### Pearsons correlation coefficients

**Fig 8:**
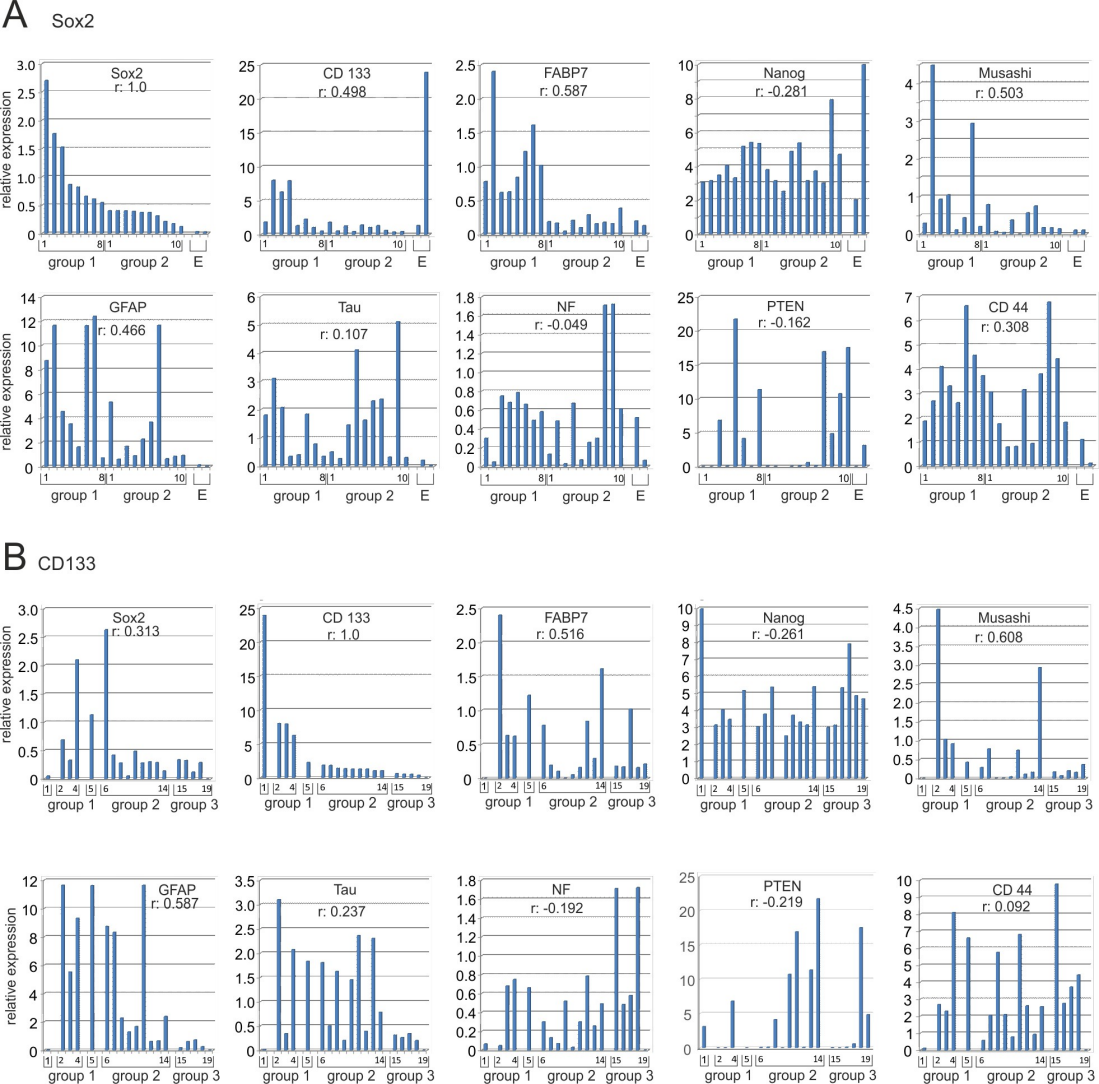
Pearson correlation coefficients (k): SLGC markers. In (**A**) cell lines were ranked according to their Sox2 expression at the time of analysis. The Actin-normalized relative expression of the respective proteins is indicated in the plots. Group 1 displayed significantly higher Sox2 levels than Group 2. Group E comprises the established cell lines U87 and CaCo2, both of which lack Sox2 expression. The ranking was as follows: [T1452, T1371, T1495-SC, T1586, T1440, T1587, T1447-SC, T1495, T1447, T1338], [T1522, T1389, T1467, T1442, T1464, T1454, T1600, T1439], [CaCo2, U87]. In **(B**) cell lines were ranked according to their CD133 expression at the time of analysis. The Actin-normalized relative expression of the respective proteins is indicated in the plots. The CD133 levels were highest in the CaCo2 cell line, followed by the SLGCs assigned to groups with decreasing CD133-levels. The non-SLGC line CaCo2, which encompasses >90% of CD133 positive cells, is indicated on the very left. The ranking was as follows: [CaCo2], [T1452, T1333, T1495-Sc], [T1447-SC], [T1586, T1442, T1587, U87, T1600, T1464, T1447, T1495, T1440], [T1522, T1371, T1389, T1467, T1439, T1454].–For abbreviations, see the legends to Figs 5 and 6.

**Fig 9:**
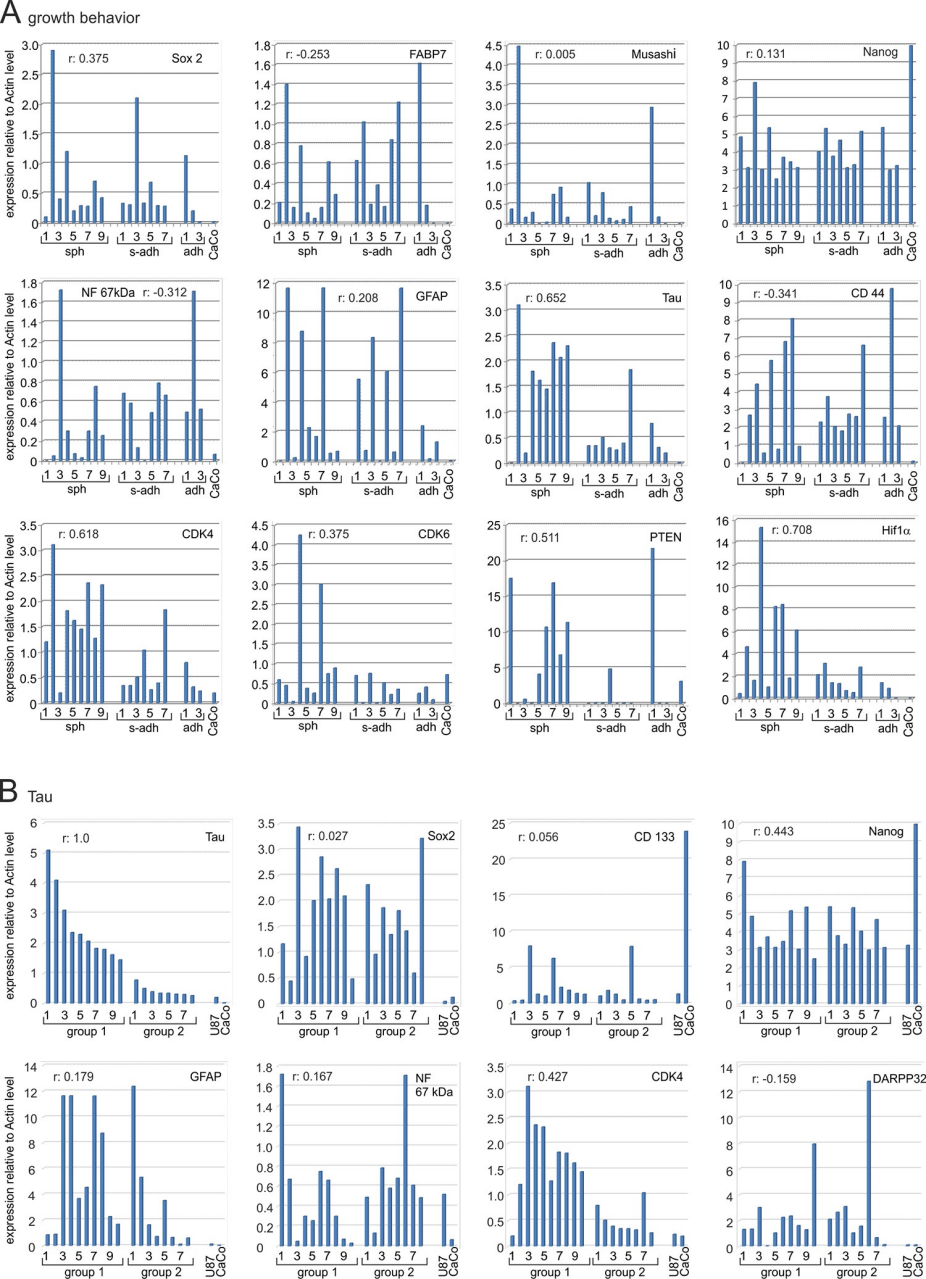
Pearson correlation coefficients (k): Growth behavior and Tau. In (**A**) Pearson correlation coefficients relating protein expression to growth behavior. The “sph” group comprises SLGC lines/cultures that solely displayed spherical growth at the time of analysis. The T1439 line is ranked first because the cultures showed single floating cells and tiny aggregates of a maximum of 10 cells. All other cell lines formed large aggregates consisting of hundreds, sometimes thousands of cells. Adherent lines (adh) formed rosettes (T1440) or foci on top of the monolayers. The ranking was as follows: [T1439, T1452, T1467, T1586, T1587, T1600, T1464, T1495SC, T1495], [T1338, T1389, T1442, T1454, T1371, T1447, T1447SC], [T1440, T1522, U87]. In (**B**) cell lines were ranked in two groups according to their Tau expression at the time of analysis. The established cell lines U87 and CaCo2 are indicated on the right. SLGC lines were ranked as follows: [T1467, T1439, T1452, T1464, T1495, T1495-SC, T1447-SC, T1586, T1587, T1600], [T1440, T1442, T1447, T1389, T1338, T1522, T1454, T1371]–For abbreviations, see the legends to Figs 2–6.

The expression data (proteome array, Western blot, and qRT-PCR) as well as the growth behavior and the numbers of cells positive determined by flow cytometry were used to calculate Pearsons correlation coefficients. This analysis encompassed Western blot data from three biological replicates, except for T1467, T1556, T1564, and T1600, as these SLGC lines ceased. The most interesting data, which is related to stemness, differentiation, and growth behavior, are summarized in Figs 8 and 9. Based on the model of cellular hierarchy, we first used Sox2 expression levels to calculate the correlation coefficients. It turned out that Sox2 expression positively correlated with that of FABP7 (+0.587), Musashi (+ 0.503), followed by CD133 (+0.498), GFAP (+0.486), AKT (+0.479), N-cadherin (+0.411) and CDK4 (+0.376). Low positive correlations (r values between +0.01 and +0.200) were revealed for, e.g., Tau, CDK6, PDGFRα, and Nanog, negative correlations (between -0.01 and– 0.3) for Hif1α, PTEN, EGFR, PDGFRβ, MERTK, and in particular E-cadherin (-0.286). When CD133 served as a reference the situation changed: similar to the situation for Sox2, the correlation coefficients between CD133 and FABP7, Musashi and GFAP levels were above r = +0.5 (Fig 8). The correlations between CD133 levels and those for Tau, Neurofilament, PTEN, Nanog and DLX2, however, were higher than those between Sox2 and these proteins. When the growth behavior was considered, which varied between adherent rosettes (T1440), semi-adherent (e.g., T1371) and pure spherical growth (e.g., T1586), the situation was also distinct. It appeared that the spherical phenotype was positively correlated with the expression of Hif1α, CDK4, Tau and PTEN. A still positive, but reduced correlation between spherical growth and the expression levels of Sox2, FABP7, Nanog, GFAP, CD133 and AKT was revealed. The correlations between spherical growth and the type II cell marker CD133 and the type III cells marker DLX2 levels were +0.056 and +0.071, respectively. Altogether, this specifies Sox2, CD133, FABP7, Musashi, Akt and GFAP as factors associated with the stemness state, whereas the spherical growth behavior appeared to be associated with PTEN expression and increased levels of Hif1α, Tau, PDGFRα and β (Fig 9A). When Tau levels served as a reference, positive correlations were determined for CDK4 (0.427) and Nanog (0.443), but not for Sox2 (+0.027), CD133 (0.056), GFAP (0.179) or DARPP32 (-0.159).

Notably, the N-cadherin levels showed a similar positive correlation (+0.41 to +0.42) to Sox2 and CD133 levels as well as spherical growth, whereas the correlations to E-cadherin were negative (-0.41 to -0.42). In addition, the MGMT levels appeared independent of stemness (Sox2/MGMT: -0.001); CD133/MGMT: -0.151) or spherical growth (sph/MGMT: -0.012). To further validate the conclusions on MGMT, we analyzed the expression of *mgmt* mRNA (RT-PCR analyzes; T1338, T1371, T1440, T1447, and T1452) and the methylation status of the *mgmt* promoter (MSP analyzes; all SLGC lines) and related the data to the Western blot data (S9 Fig). We identified three SLGC lines (T1338, T1440, T1556) that displayed a pure m-status in MSP analyzes, that was stable over increasing passages, whereas T1371 showed an enduring pure u-status. Moreover, the T1338, T1440, and T1371 clones that had been isolated by limited dilution assays showed the same behavior as the corresponding parental SLGC lines. All other SLGC lines showed a mixed m/u-status, i.e., the primers specifying the m- and the u-status, respectively, allowed for the amplification of PCR products. Notably, the m/u status was also revealed in the tumor specimen, which served as the source for the respective SLGC lines (examples in S9 Fig). The SLGC lines with a m/u status could be grouped in lines with m>u (T1442, T1452, T1464, and T1495), m~u (T1389, T1522, T1524, T1549, and T1564) and m<u (T1586, T1587, T1447, and T1600). In cases of SLGC lines with a m/u-status, in which clones from limited dilution assays were available (T1452, T1464, T1495, T1522, and T1586), the methylation status and MGMT protein expression were additionally determined in the clones. For T1452, T1522 and T1586 the data for the SLGC line (mc) and the clones were similar. In contrast, the T1464 mc displayed a m>u status, whereas the clones varied between m>u and m~u and the T1495 mc/T1495SC showed less methylation than the T1495 clones. None of the clones (T1452, T1464, T1495, T1522, and T1586), however, carried a pure m- or u-status, indicating that the cells in the SLGC (mc) cultures harbored partially methylated *mgmt* promoters. In Western blot analyses using 20 μg whole cell extract the MGMT protein was detectable when the methylation status was m~u or m<u. Moreover, *mgmt* mRNA was only revealed by RT-PCR in SLGC lines with a pure (T1371) or a primarily (T1447) u-status. Taken together, this indicated that the Western blot data well reflected the methylation status of the *mgmt* promoter and supported the above conclusion that growth behavior and stemness of SLGCs are independent of the *mgmt* promoter’s methylation status.

The highest positive correlation coefficients (in descending order) were determined for the pairs Sox2/CDK6, Sox2/GFAP, Sox2/integrin-αv and Sox2/CD133 and the pairs CDK6/GFAP and CDK6/integrin-αv. The r-values for the pairs CDK4/Tau, CDK4/GFAP and PDGFRβ/Tau were also among the most positive ones (S8D and S8E Fig).

## Discussion

A large amount of data has accumulated since the first evidences for the existence of cancer stem cells in GBM and GS were published [22, 23, 63, 64] (for overview, see, [24, 25]). On the basis of the first reports, many researchers utilized CD133-positive GBM cells in their experiments, others used established cell lines such as U87 or U251 (e.g., [32, 65–67]). The situation is further complicated by the intertumor and intratumor heterogeneity of GBMs [15–21] and the possible impact of the cell origin on therapy sensitivity [37]. Moreover, many candidate markers have been proposed, none of which turned out to be universally expressed. In particular, the significance for CD133 is still under debate, as several authors argue for a role of CD133 in maintenance of the stem cell state, regulation of signaling cascades, and therapy sensitivity, whereas others propose a cell-cycle and differentiation stage-associated expression, respectively (overview in [24, 25]).

In the present work we set out to identify cell surface markers that would be applicable to glioma stem cells (SLGCs) from malignant brain tumors with distinct sets of founder mutations. For this purpose, we used SLGCs from GBMs and GSs with (i) Tp53^WT^ or Tp53^mut^ and (ii) PTEN^WT^ or PTEN loss. All SLGC lines showed the IDH1 wildtype and expressed Sox2, CD133, Vimentin, SSEA1/CD15 and Nestin. A detailed immuncytochemistry analysis confirmed the model of cellular hierarchy [28], with several modifications: Firstly, all SLGC lines used in the study established a cellular hierarchy in cultures, in which some of them consisted of >90% type I cells (e.g., T1338/T1338 cl1, T1452, and T1586), whereas others (e.g., T1495 and T1447) established a pronounced hierarchy. These patterns remained stable for most SLGC lines during continuous passaging, suggesting an endogenous regulatory mechanism. Secondly, the largest and most rapidly growing orthotopic tumors were derived from the SLGC lines with a pronounced cellular hierarchy (e.g., T1447, T1495), whereas the SLGC lines with the highest degree of stemness displayed a lower efficacy (e.g., T1452, T1586) or did not give rise to any tumor (T1338/T1338 cl1). Thirdly, GFAP was associated with high Sox2 expression in some SLGC lines (e.g., T1586, T1452, and T1440) and served as a differentiation marker in others (e.g., T1338, T1495, and T1522). With the exception of T1338/T1338 cl1 (human cells survived in the mouse brains for 6 months but did not expand to generate a tumor), all SLGC lines generated orthotopic tumors in SCID mice, in which the human cells (revealed by the human-specific marker Stem 121) expressed high levels of Sox2, irrespective of the degree of cellular hierarchy observed in the xenotranspanted cultures. In addition, we were able to show that Sox2 positive cells are organized in clusters in human tumor specimens.

We used SLGC lines with a PTEN^WT^/p53^WT^ (e.g., T1464), PTEN^mut^/p53^WT^ (e.g., 1338), PTEN^WT^/p53^mut^ (e.g., 1600), PTEN^mut^/p53^mut^ (e.g., T1371) genotype for the determination of the expression of cell surface proteins. In this connection, we included three SLGC lines from GSs and one derived from an orthotopic tumor (i.e., T1495-SC). By means of cophenetic correlation coefficient calculation (CCC) [34, 35] we could assign the 12 SLGC lines to five distinct metaprofiles (MP1-MP5). Based on these data several general statements could be made: (i) In comparison to the established cell lines U87 (human GBM) and CaCo2 (human colon cancer) all SLGC lines showed similar metaprofiles, indicating that the stem-like glioma cells are distinct from the non-SLGC GBM line U87 and the CD133+ Caco2 cell line; (ii) the highest similarity could be assigned to T1495 and T1495-SC, attesting a good preservation of the phenotype during expansion in the mouse brain; (iii) the three GS lines were assigned to two distinct metaprofiles, which additionally encompassed GBM-derived SLGCs, indicating that the differences between SLGCs from GSs and GBMs may be smaller than those between SLGC lines derived from distinct GBMs; (iv) the growth behavior (adherent rosettes, semi-adherent or pure spherical growth) appeared unrelated to the metaprofiles; (v) SLGCs with a high degree of stemness and pronounced cellular hierarchy, respectively, were present in all metaprofiles; (vi) whether SLGC lines co-expressed GFAP and Sox2 appeared likewise unrelated.

All five metaprofiles are characterized by high levels of the extracellular matrix metalloproteinase inducer EMMRPIN/CD147, which has been associated with adverse tumor outcomes, such as reduced overall survival and progression-free survival, as well as a high risk for chemotherapy resistance or metastasis in distinct types of cancer [39]. In addition, the expression of Cathepsin D was high in all SLGC lines and particularly in those assigned to MP2, MP4, and MP5. This is in agreement with reports describing the importance of lysosomal enzymes for brain tumor invasiveness [40]. Moreover, a recent report indicated that inhibition of Cathepsin D increased the radiosensitivity of U251 GBM cells [67]. Besides of Cathepsin D two members of the MMP (matrix metalloprotease) subfamily, known as disintegrin and metalloproteases (ADAM), characterized the metaprofiles. In particular, ADAM9 appeared specific to MP1 and ADAM17 to MP5, in which MP1 is additionally characterized by beta-integrins and CD99, and MP5 by neural proteins, such as NCAM-L1. ADAM9 and -17 expression have been associated with tumor grade and were proposed as potential diagnostic/prognostic biomarkers [41]. Our data suggest, that the potential use of ADAM9/-17 as biomarkers may be extended to SLGCs. CD99 is a transmembrane protein overexpressed in several malignancies, including astrocytomas of different malignant grades, which might impact on migration and invasiveness of cancer cells [68]. CD99 was expressed on all SLGC lines, though at distinct levels, and appeared specific for MP1 and MP3.

As the expression of β-integrins was high in all SLGC lines and Intβ1, Intβ3, and Intβ5 characterized MP1 and Intαv MP2, respectively, we analyzed the expression of integrins in more detail and included additional SLGC lines in these studies. It turned out that Intβ1 is the β-integrin with the highest expression in the 24 SLGC lines analyzed. Regarding the alpha-integrins the expression of Intαv was particularly high, and high amounts of Intαv- and Intα6-positive cells were present in the same SLGC cultures. Under differentiation conditions, Intα6 levels remained largely unchanged, whereas Intαv was upregulated in some and downregulated in other SLGC lines. Considering the Sox2 levels in the respective cultures, our data would rather not support previous reports that proposed that Intα6 supports the stemness state and serves as a SLGC marker [50, 51]. Lectins, such as Galectin-3 and -3b specified MP4/MP5 and MP3/MP4, respectively. Galectin levels seem to be important in the context of neurodegenerative disease [49] and Galectin-3 binding proteins were proposed to potentially serve as early biomarkers for glioma [69].

Our cophenetic correlation coefficient calculations (CCC) are in support of EMMPRIN/CD147, CD99, Cathepsin D, Intβ1, ADAM9/-17, and Galectin-3 being suitable biomarkers for glioma cells and potentially, SLGCs. Another cell surface protein with a general high expression is N-cadherin. We could show, that N-cadherin largely dominates over E-cadherin in all SLGCs. In addition, the E-cadherin expression was comparable to that of VE-cadherin/CD144 in proliferating cultures. When we used our Western blot data for the calculation of Pearson correlation coefficients, we observed a positive correlation between the N-cadherin levels and the expression of Sox2 (~+0.41) and CD133 (~+0.41) and a similar negative correlation for E-cadherin and Sox2 and CD133. Notably, VE-Cadherin, as well as PE-CAM-1/CD31 became upregulated under differentiation conditions in the majority of SLGC cultures, which would be in agreement with previous reports describing the differentiation of stem-like GBM cells into endothelial-like cells [52].

The situation for the cell surface protein CD44, which may function as a receptor for hyaluronan appeared more complicated. Immunocytochemistry and flow cytometry documented CD44 expression on more than 95–99% of the cells in cultures of all SLGC lines under proliferation and differentiation conditions. Based on our Western blot data, which revealed variable CD44 levels, the Pearson correlation coefficient calculation indicated a weak positive correlation between Sox2 levels, no correlation with CD133 levels, and a negative correlation with spherical growth. Considering our previous analysis of tumor slices [23] and the present work, CD44 may serve as a general GBM marker rather than a SLGC marker. A previous report [70] identified CD44 as the sole marker analyzed that exhibited a positive correlation with GBM cell radioresistance, and found a negative correlation between radioresistance and the expression of SLGC-associated factors, such as Nestin, Sox2, CD133, and Musashi.

When we determined factors positively related to the expression of Sox2, we identified FABP7 and Musashi, followed by CD133 and GFAP. The same type of calculation using CD133-levels instead revealed a positive correlation between CD133 levels and those of Musashi, followed by FABP7, GFAP, and at last Sox2. In part this confirms the data by Chen et al. [28], who assigned FABP7 expression to Sox2+/CD133- type I and Sox2+/CD133+ cells type II cells. Spherical growth displayed a good positive correlation with Hif1α, CDK4, Tau, and PTEN expression, but only a low positive correlation with the expression of stemness-associated factors. That PTEN expression rather than stemness is associated with spherical growth has also been proposed by Chen et al. [28]. Notably, both MGMT (protein expression and *mgmt* promoter methylation) and ABCG2 (protein levels) appeared unrelated to spherical growth, as well as to Sox2 and CD133 levels, indicating that these proteins are not specifically associated with SLGCs or growth behavior. The same applies to Nanog, CDK6, DLX2, and the receptor tyrosine kinases, except for PDGFRβ.

Though the positive correlation between Tau levels and spherical growth might suggest induction of differentiation, we observed Tau expression in SLGCs with high Sox2 levels, detected Tau-positive tumor cells in GBM slices, and observed Tau in all SLGC lines. Hence, we consider Tau, in addition to Nestin, as a neural protein universally expressed in proliferating SLGC lines, which is not the case for another microtubule-associated neuronal protein (MAP2) or the neuronal βIII-tubulin, synaptophysin and neurofilaments. However, it appeared that the enduring growth of SLGCs under differentiation conditions ultimately led to higher Tau levels. Pearson correlation coefficients identified a positive correlation between high Tau expression and increased Nanog, PDGFRβ and CDK4 levels, suggesting that Nanog and CDK4 levels are also related to differentiation. The proteins DARPP32 [56] and the Dopa-Decarboxylase AADC/DCC [57], which are expressed in dopaminergic neurons but also in certain cancer cells [71–73] displayed high expression levels in two of the GS- and three of the GBM-derived cell lines. Co-expression of DARPP32 and AADC/DDC was observed in the two GS lines T1371 and T1447 and the GBM-derived cell lines T1442 and T1338. Based on the expression of the stemness-associated proteins DARPP32 and AADC/DDC expression is not related to stemness or differentiation. The presence of these proteins could possibly identify neural progenitors as the cells of origin. This would be supported by the particular high expression of the transcription factor Pax6, which regulates neuronal specification during embryogenesis [74] and might impact on the TMZ sensitivity of GBM cells [75].

Amongst the receptor tyrosine kinases analyzed, the expression of all four members of the HER (human EGFR receptor) family was detected by the proteome array, and none of them was specific for any of the metaprofiles. Nonetheless, EGFR/HER1 has been implicated in the classification of GBM subtypes, together with PDGFRα and is used to distinguish between GBM subtypes [16–21]. We observed expression of EGFR in all SLGC lines analyzed, but high expression of a truncated EGFR was only observed in one out of 24 SLGC lines and disappeared during ongoing passaging. In addition, increased but stable EGFR levels were detected in two cell lines. In addition, truncated or larger versions of the EGFR were observed. The expression of high levels of EGFR, both PDGF receptors, or the Mer tyrosine kinase (MERTK) was not mutually exclusive, suggesting that co-expression of several receptor tyrosine kinases is a property of many SLGC lines. This could promote the hypothesis that the GBM classification, which indicated differential expression of receptor tyrosine kinases in GBM subtypes, would not be applicable to SLGCs. Moreover, a potential general significance of the EGFR seems likely, as SLGCs synthesize not only EGF but also additional EGFR/HER1 activating ligands (i.e., TGFα, HB-EGF, Amphiregulin, Epiregulin). The central role of EGFR in all SLGC lines would not be surprising, as the EGFR receptor also drives the proliferation of neural stem cells and progenitors [53]. The co-expression of receptors and ligands, suggesting autocrine stimulation via EGRR signaling cascades, was further supported by our growth factor ELISA and growth curves. As discussed above, the expression of PDGFRβ but not PDGFRα might be associated with an induction of differentiation. The expression of the PDGFRα was very heterogeneous in many respects. This included the presence of two truncated forms in addition to the full-length receptor and a distinct subcellular location. For example, SLGC lines with general low levels of PDGFRα showed a receptor location in the cytoplasm of dividing cells. On the contrary, SLGC lines with high PDGFRα expression showed an inhomogeneous distribution of the receptors in the plasma membrane, presumably in lipid rafts.

Altogether, the present work extended and modified the model of cellular hierarchy [28] to include GBM- and GS-derived SLGCs with distinct p53 and PTEN states. Moreover, we provided evidence for a general association of FABP7 and Musashi with the stemness state but could not support an association of CD44, Hif1α, Intα6, Nanog or MGMT. In addition, the present data supports the hypothesis that the expression of GFAP, DARPP32, AADC/DDC and Pax6 might reflect the origin of SLGCs. Moreover, the data are in support of a general role of EGFR in SLGCs and a general neural signature of SLGCs from GBMs and GSs. Cell surface proteins that might represent promising candidates for targeting GBM and GS cells include Cathepsin D, CD99, EMMPRIN/CD147, Intβ1, the Galectins of the subfamily 3, and N-cadherin, all of which appeared rather independent of the stemness state.

## Supporting information

S1 Fig Characterization of SLGC lines. **(A)** Immunocytochemistry analyzes: primary antibodies indicated in the figure were revealed with goat anti-mouse DyLight®488 (green) and goat anti-rabbit Cy3 (red), respectively. Except for T1389, all microphotographs are z-stacks. DAPI (4′,6-diamidino-2-phenylindole) nuclear counterstain (blue) is shown. Bars, 50 μm. CD15, Stage-Specific Embryonic Antigen1 (SSEA1); CD133, Prominin-1; GFAP, glial fibrillary acidic protein; Nestin, *type* IV intermediate filament; Sox2, SRY (sex determining region Y)-box transcription factor 2; Vimentin, *type* III intermediate filament.—(**B**) Capacity of SLGCs to generate orthotopic tumors in the SCID mouse model. HE (hematoxylin and Eosin) staining of 4.5 μm slices. The SLGCs were inoculated into the right hemisphere. Arrows point to the inoculation site, and arrowheads mark tumor borders. Bars, 50 μm; SC, cell line derived from an orthotopic tumor.—(**C**) Coronal T2-weighted MRI scan of SCID mice 6 weeks after inoculation with T1447 and 6 months after inoculation with T1338 cells. The left panel of MRI images depicts distinct sections of the same orthotopic tumor. The MRI of the T1338-inoculated mouse did not show any signs of tumor formation in the entire scan.—(**D**) Immunohistochemistry analysis of two SCID mouse brains (a, b) inoculated with T1338 cells. Triple stain with the antibody pairs rabbit-Sox2/goat-anti-rabbit-Cy3 and mouse-Stem-121/goat-anti-mouse-DyLight® 488, followed by nuclear counterstain with DAPI. Staining of 4.5 μm slices was done 12 months after xenotransplantation. White arrows point to Sox2-positive T1338 cells, red arrow heads to erythrocytes. Bars, 50 μm.
(TIF)

S2 Fig Matrix reconstruction with metaprofiles and their signature proteins. (Top left) The plot shows the experimentally measured protein expression X for all 12 cell lines. (Top right) This plot shows the reconstruction of these patterns by the 5 metaprofiles MP1-5. (Bottom) For the reconstruction, only metaprofiles with their signature, i.e., expression values above 90% (left) or above 95% (right) percentile, were used.
(TIF)

S3 Fig Expression of integrins. (**A**) Comparison of the relative amounts of α-integrins expressed in the SLGC lines indicated (data from proteome array). Based on the assumption that the antibodies spotted on the filters of the proteome array possessed similar K_D_s, the relative expression of the integrins was calculated. The sum of the signals of all integrins was arbitrary set at 100%. The bars indicate to what percentage the individual α-integrins contribute to this expression. (**B**) Similar analysis as in (A) comparing the relative expression of the integrins β1, β2, β3, β4, β5, and β6. (**C**) Western blot analysis using antibodies directed against the integrins αv, β1, β3, and β5. The loading controls Actin and GAPDH (glyceraldehyde-3-phosphate dehydrogenase) are shown below the corresponding blots. (**D**) Flow cytometry analysis experiment using early passages of SLGCs. The established non-SLGC cells lines U87 (glioblastoma) and CaCo2 (colon carcinoma) lack stemness. (**E**) Expression of mRNAs coding for the β-Integrins indicated. Total RNA was isolated from SLGC lines, subjected to reverse transcription and qRT- PCR analysis. Signal intensities were normalized against *gapdh*. (F) A similar experiment as in (E) investigating the effect of serum (10% fetal calf serum) on the expression of α- and β-integrin mRNAs, respectively. In order to highlight serum-mediated changes, the relative expression obtained with RNAs isolated from the corresponding serum-free cultures was arbitrary set at 1.- SC, derived from an orthotopic tumor.
(TIF)

S4 Fig Expression of cadherins and CAMs (cell adhesion molecules). (**A**) Comparison of the relative amounts of Cadherins expressed in the SLGC lines indicated (data from proteome arrays). Based on the assumption that the antibodies spotted on the filters of the proteome arrays possessed similar K_D_s, the relative expression of the cadherins was calculated. The sum of the signals of all cadherins was arbitrary set at 100%. The bars indicate to what percentage the individual cadherins contribute to this expression. C4, C11, and C13, cadherins 4, 11 and 13; E-C, E-cadherin; N-C, N-cadherin; P-C, placental cadherin; VE-C, VE-(vascular endothelial) cadherin/CD144. (**B**) Quantification of Western blot analyzes investigating the expression of N-cadherin in the SLGC lines indicated. Bars represent the mean expression normalized against Actin, whiskers represent the variation between technical replicates. (**C**) Similar experiment as in Fig 5 using biological replicates of the various SLGC lines (flow cytometry analysis). The established cell lines U87 (glioblastoma) and/or CaCo2 (colon carcinoma), which lack stemness were used as references. The antibodies bind to the VE-cadherin/CD144 and PECAM-1 (platelet endothelial cell adhesion molecule -1)/CD31, respectively. Bars depict the percentage of positive cells determined in one representative experiment. (**D**) Comparison of the relative amounts of CAMs expressed in the SLGC lines indicated (data from proteome arrays). The sum of the signals of all CAMs was arbitrary set at 100%. The bars indicate to what percentage the individual CAMs contribute to the total CAM expression. Cea-CAM-5 or -1, Carcinoembryonic antigen-related cell adhesion molecule -5 or -1; CHL-1, cell adhesion molecule L1-like; N-CAM, nerve cell adhesion molecule; VCAM, Vascular cell adhesion protein 1; PECAM, platelet endothelial cell adhesion molecule; ICAM-2, intercellular adhesion molecule 2; Neurotrimin, GPI-anchored cell adhesion molecule that might promote neurite outgrowth; NCAM-1, neural adhesion molecule-1; NCAM-L1, neural cell adhesion molecule L1; EpCAM, epithelial cell adhesion molecule.
(TIF)

S5 Fig Expression analyses HER family. **(A) Left**: Comparison of the relative amounts of members of the EGF-receptor/HER family (data from proteome arrays). Based on the assumption that the antibodies spotted on the filters of the proteome arrays possessed similar K_D_s, the relative expression was calculated. The sum of the signals from the four receptors was arbitrary set at 100%. The bars indicate to what percentage the individual HERs contribute to this expression. **Right**: similar comparison considering Epiregulin, HB-EGF, Amphiregulin, all of which may activate EGFR/HER1. (**B**) Western blot analysis of the expression of the proteins indicated in the blots. Upper panel of left: The full-length(fl) EGF (epidermal growth factor) receptor and truncated EGFR proteins are shown. Right: Similar blots using antibodies against the PDGF (platelet-derived growth factor) receptors α and β. Lower panel on left: comparison of GFAP and CD133 expression. (**C**) Relative expression of CDK4 and CDK6: expression was normalized against the corresponding Actin signals. The mean values of three independent biological replicates are shown; whiskers indicate variations. (**D**) RT-PCR analysis of p53 transcripts. p53* indicates that the PCR product is longer than usual due to a mutation in a splice site. The triangle indicates increasing passages (p2, p5, p9, p26, p35); g*apdh* served as a reference gene. (**E**) Western blot analysis of the expression of IDH1 (IDH1: cytoplasmic, 46.7 kDa), IDH2 (IDH2: mitochondrial isoform 1: 50.9 kDa, isoform 2: 45.2 kDa) and p53 in the SLGC lines indicated. The loading control GAPDH is shown below the respective blots. Brackets indicate that the analyses were performed using the same nitrocellulose filter.—GAPDH, glycerol aldehyde-3-phosphate dehydrogenase.
(TIF)

S6 Fig Growth factor requirements and growth curves. (**A**) Expression (RT-PCR analysis) of mRNAs coding for EGF (epidermal growth factor), TGFα (transforming growth factor α), HB-EGF (Heparin-binding EGF-like growth factor), and bFGF (basic fibroblast growth factor). Bars depict the mean of a minimum of three replicates, whiskers the standard deviation. Significant differences relative to the expression in the T1338 cell line are indicated (**, p<0.005; ANOVA). (**B**) Effects of growth factor depletion on proliferation (BrdU ELISA). The combination of growth factors added into the medium is indicated by distinct levels of gray(white: EGF/bFGF; light grey: EGF; dark grey: bFGF; black: no growth factor added). The bars depict mean values and standard deviations. Significant reduction of BrdU incorporation is indicated by p-values (**, p<0.005). (**C**) Growth curves of the SLGC lines indicated were performed in the presence of EGF (red line), bFGF (black line) or both (blue line), or in the absence of growth factors (violet line). After six days (d6) cells were re-plated for studies with extended incubation times. Values for d9 were determined from both the original and the replated cultures. Significant differences between the growth curves at specific time points are indicated by p-values (*, p<0.05 and **, p<0.005; ANOVA). (**D**) Growth factor ELISA. The amounts of EGF and bFGF present in T1371 and T1447 cultures were determined at days d2 and d9 after plating. The assays were performed in DMEM/Ham’s F12 containing fetal calf serum (10% FCS) or the serum supplements BIT (bovine albumin, insulin, and transferrin) and B27, respectively. The presence of exogenous growth factors is indicated by EGF/bFGF. Each assay encompassed a minimum of four replicates. Significant differences are indicated (**, p<0.005).
(TIF)

S7 Fig Expression of Notch, VEGFRs, Alk6, and CD58. (**A**) Expression of Notch and Notch-ligand–(Left): Comparison of the relative expression of Notch and the Notch ligand Jagged in the SLGC lines indicated (data from proteome arrays). Right: Western blot analysis of Notch-1 expression in the SLGC lines indicated. The loading control Actin is shown below the blot. The bar graph below depicts the mean values of Notch-1 expression (normalized against Actin) and their variation in technical replicates of the SLGC lines indicated. (**B**) Relative expression of VEGF-R1 and–R2. Comparison of the relative expression of the two receptors in the SLGC lines indicated (data from proteome arrays). (**C**) Relative expression of the BMP (bone morphogenetic protein) receptor IB/Alk6, LRP (low density lipoprotein-related protein 1) and CD58/LFA-3 (lymphocyte-associated antigen-3) in the SLGC lines indicated (proteome array data). The bars depict the mean from two independent arrays, the whiskers show the variation.
(TIF)

S8 Fig Expression of proteases and Pearson correlation. (**A**) Comparison of the relative amounts of ADAM (a disintegrin and metalloprotease domain) proteases as well as BACE (beta-secretase) and MMP2 (matrix metallopeptidase 2) in the SLGC lines indicated (data from proteome array). Based on the assumption that the antibodies spotted on the filters of the proteome array possessed similar K_D_s, the relative expression of the proteases was calculated. The sum of the signals of all proteases was arbitrary set at 100%. The bars indicate to what percentage the individual proteases contribute to this expression. (**B**) Similar assay as in (A). The relative Cathepsin D levels were compared to the relative expression of all other proteases, depicted in panel (A). (**C**) Similar analysis as in (A) comparing the relative expression of the protease inhibitors TIMP (tissue inhibitor of metalloproteinases) 1, 2, 3 and 4.–Parts **D and E** graphically summarize some of the correlation data shown in Figs 8 and 9. In (**B**) the coefficients are indicated on the y-axis, highlighting expression levels with a positive correlation to the Sox2 (blue) or CD133 (violet) dots. In (**C**) the Pearson correlation coefficients were calculated relative to the levels of the neural proteins Tau and GFAP, as well as the hyaluronan receptor CD44, the integrin αv, and the N- and E-cadherin, respectively. In all cases, the expression levels that entered the calculations were determined with the same whole cell extracts. The calculations were confirmed with biological replicates.
(TIF)

S9 Fig MGMT-status. (**A**) RT-PCR analysis of *mgmt* mRNA expression; *gapdh* served as a control. T1371 was studied during passages p2, p6, and p26. (**B, C**) Examples of methylation-specific PCR (MSP) using gDNA from tumor tissue as well as SLGC lines (mc) or clones (cl) obtained by a limited dilution assay. M (methylated status), PCR using the primers MGMT^meth^-F/MGMT^meth^-R; U (unmethylated status), PCR using the primers MGMT^un^/MGMT^un^-R. Arrows point to the specific PCR products, *, labels unused primers/frequently seen byproducts of <40 bps. The box depicts cell lines with a pure **m**- or **u**- status, including SLGCs as well as the non-SLGC GBM line U87 and the colon cancer cell line CaCo2, which served as controls in various experiments. (**D**) Summary of the quantification of the MSP data; mc/SC, SLGC/SCID lines; cl, clones derived from SLGC lines by means of limited dilution assays.–For mc/SC the mean values ± SEM from a minimum of three biological replicates are shown. For clones, the bars represent the mean values calculated from all clones together, and whiskers indicate the variations between the relative percentages of the PCR products determined for the clones. Altogether, six T1338, four T1371, five T1452, three T1447, six T1464, seven T1522 and five T1586 clones were included in the study. (E) Western blot analysis of the expression of the MGMT protein.–Altogether, six T1338, four T1371, three T1447, six T1464, seven T1522 and five T1586 clones were included in the study.
(TIF)

S1 Table GBM; glioblastoma multiforme; GS, gliosarcoma; GS*, recurrent gliosarcoma; suffix “SC”, indicates that the cell lines was established from an orthotopic tumor grown in a SCID mouse (SC2, was derived from xenotransplanted T1495-SC); PTEN, *Phosphatase and tensin homolog;* +/loss*, PTEN status in T1440 subpopulations is +/+, +/- or -/-; T1389 mutant*, subpopulations with mixed Tp53 status in exons 5 and 6; T1338 WT*, a subpopulation of T1338 cells is heterozygote for Tp53 mutation; RTK, receptor tyrosine kinase, IDH, isocitrate dehydrogenase; E, [EGFR] epidermal growth factor receptor; E^§^, amplification of truncated EGFR; Pα, Pβ, platelet-derived growth factor [PDGF] receptors α and β; MERTK, tyrosine protein kinase Mer—n.t., not tested. (DOCX)

S1 Raw images Raw data and uncropped blots are depicted in the supplementary file. (PDF)

S1 Appendix Additional data (xenotransplantation of SCID mice; additional flow cytometry, MSP and western blot data) are available via. (PDF)
