# Supplementary figures and images for: Molecular signature of stem-like glioma cells (SLGCs) from human glioblastoma and gliosarcoma
Source: PLoS One. 2024 Feb 2;19(2):e0291368. doi: 10.1371/journal.pone.0291368 (PMC10836714; doi:10.1371/journal.pone.0291368)

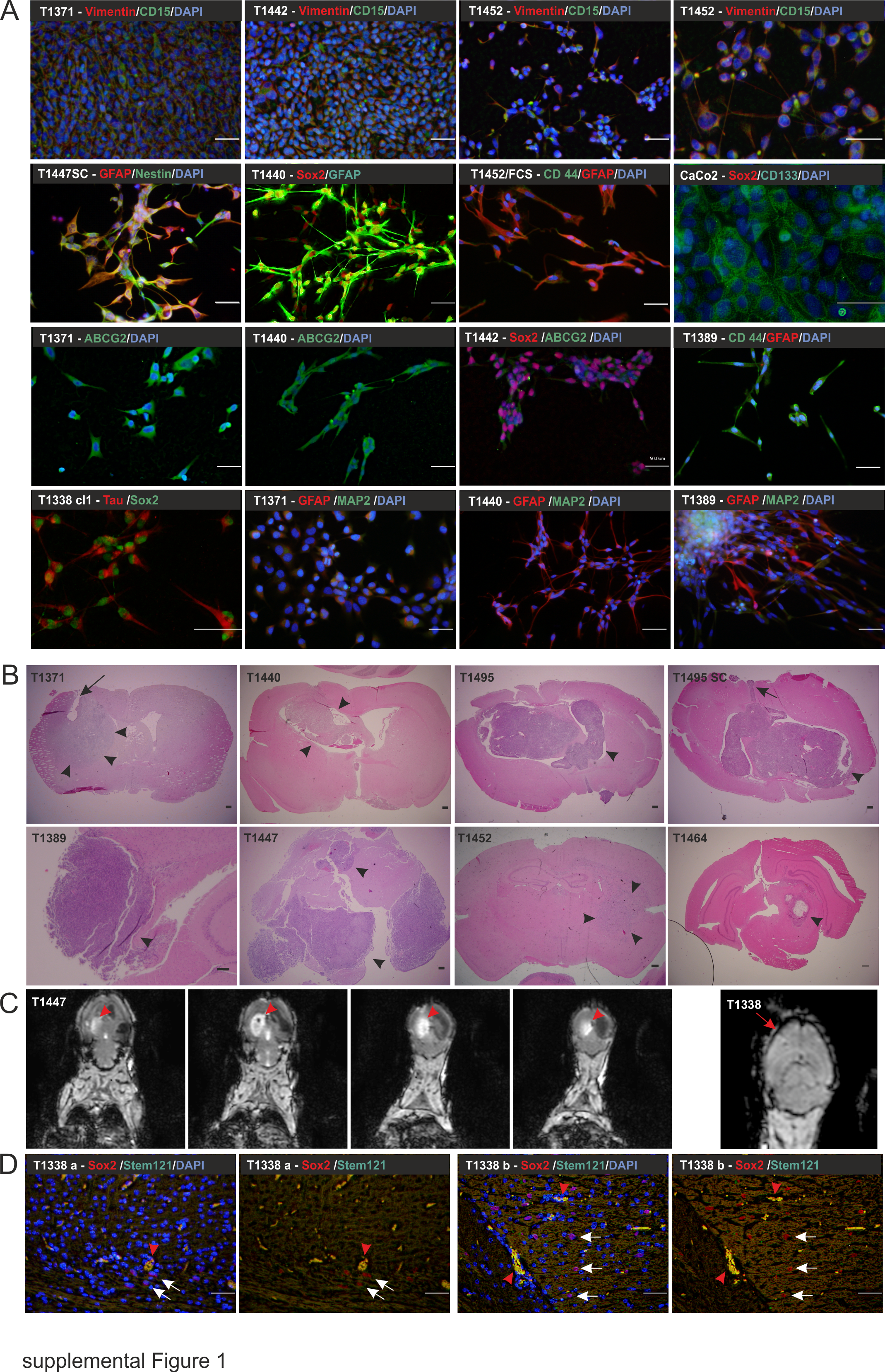

Supplement: S1 Fig — (A) Immunocytochemistry analyzes: primary antibodies indicated in the figure were revealed with goat anti-mouse DyLight®488 (green) and goat anti-rabbit Cy3 (red), respectively. Except for T1389, all microphotographs are z-stacks. DAPI (4′,6-diamidino-2-phenylindole) nuclear counterstain (blue) is shown. Bars, 50 μm. CD15, Stage-Specific Embryonic Antigen1 (SSEA1); CD133, Prominin-1; GFAP, glial fibrillary acidic protein; Nestin, type IV intermediate filament; Sox2, SRY (sex determining region Y)-box transcription factor 2; Vimentin, type III intermediate filament.—(B) Capacity of SLGCs to generate orthotopic tumors in the SCID mouse model. HE (hematoxylin and Eosin) staining of 4.5 μm slices. The SLGCs were inoculated into the right hemisphere. Arrows point to the inoculation site, and arrowheads mark tumor borders. Bars, 50 μm; SC, cell line derived from an orthotopic tumor.—(C) Coronal T2-weighted MRI scan of SCID mice 6 weeks after inoculation with T1447 and 6 months after inoculation with T1338 cells. The left panel of MRI images depicts distinct sections of the same orthotopic tumor. The MRI of the T1338-inoculated mouse did not show any signs of tumor formation in the entire scan.—(D) Immunohistochemistry analysis of two SCID mouse brains (a, b) inoculated with T1338 cells. Triple stain with the antibody pairs rabbit-Sox2/goat-anti-rabbit-Cy3 and mouse-Stem-121/goat-anti-mouse-DyLight® 488, followed by nuclear counterstain with DAPI. Staining of 4.5 μm slices was done 12 months after xenotransplantation. White arrows point to Sox2-positive T1338 cells, red arrow heads to erythrocytes. Bars, 50 μm. (TIF) [file pone.0291368.s001.tif]

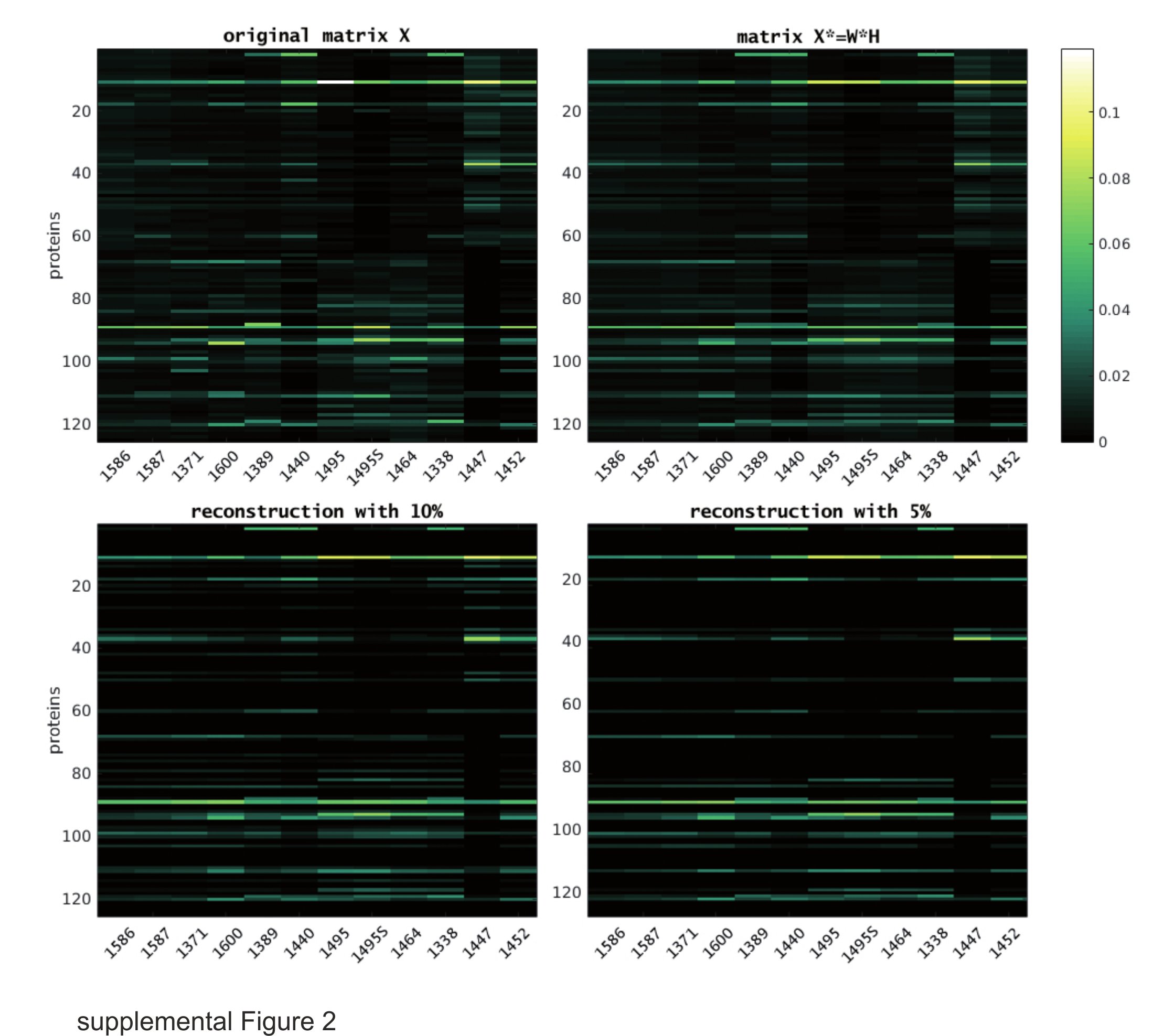

Supplement: S2 Fig — (Top left) The plot shows the experimentally measured protein expression X for all 12 cell lines. (Top right) This plot shows the reconstruction of these patterns by the 5 metaprofiles MP1-5. (Bottom) For the reconstruction, only metaprofiles with their signature, i.e., expression values above 90% (left) or above 95% (right) percentile, were used. (TIF) [file pone.0291368.s002.tif]

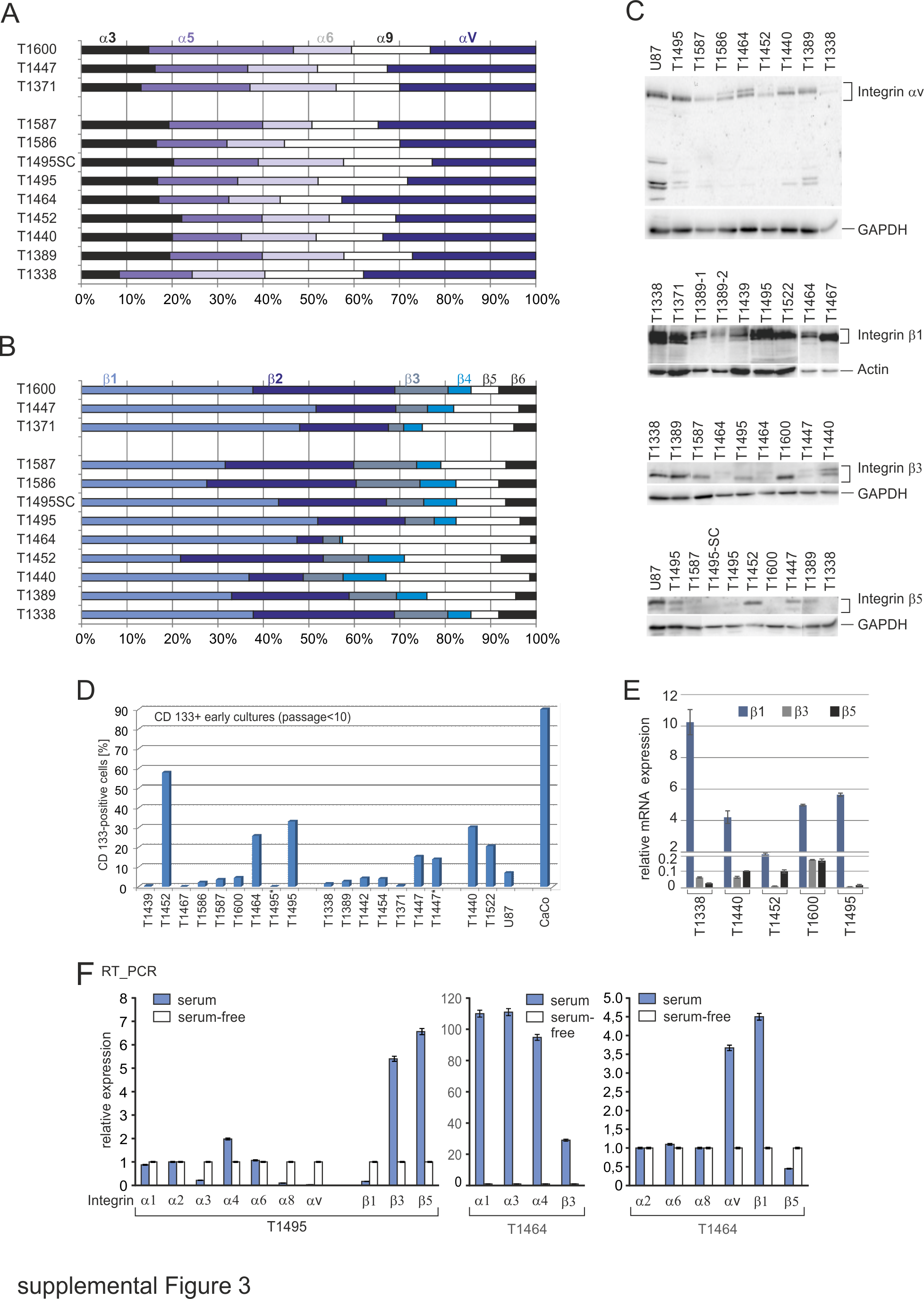

Supplement: S3 Fig — (A) Comparison of the relative amounts of α-integrins expressed in the SLGC lines indicated (data from proteome array). Based on the assumption that the antibodies spotted on the filters of the proteome array possessed similar KDs, the relative expression of the integrins was calculated. The sum of the signals of all integrins was arbitrary set at 100%. The bars indicate to what percentage the individual α-integrins contribute to this expression. (B) Similar analysis as in (A) comparing the relative expression of the integrins β1, β2, β3, β4, β5, and β6. (C) Western blot analysis using antibodies directed against the integrins αv, β1, β3, and β5. The loading controls Actin and GAPDH (glyceraldehyde-3-phosphate dehydrogenase) are shown below the corresponding blots. (D) Flow cytometry analysis experiment using early passages of SLGCs. The established non-SLGC cells lines U87 (glioblastoma) and CaCo2 (colon carcinoma) lack stemness. (E) Expression of mRNAs coding for the β-Integrins indicated. Total RNA was isolated from SLGC lines, subjected to reverse transcription and qRT- PCR analysis. Signal intensities were normalized against gapdh. (F) A similar experiment as in (E) investigating the effect of serum (10% fetal calf serum) on the expression of α- and β-integrin mRNAs, respectively. In order to highlight serum-mediated changes, the relative expression obtained with RNAs isolated from the corresponding serum-free cultures was arbitrary set at 1.- SC, derived from an orthotopic tumor. (TIF) [file pone.0291368.s003.tif]

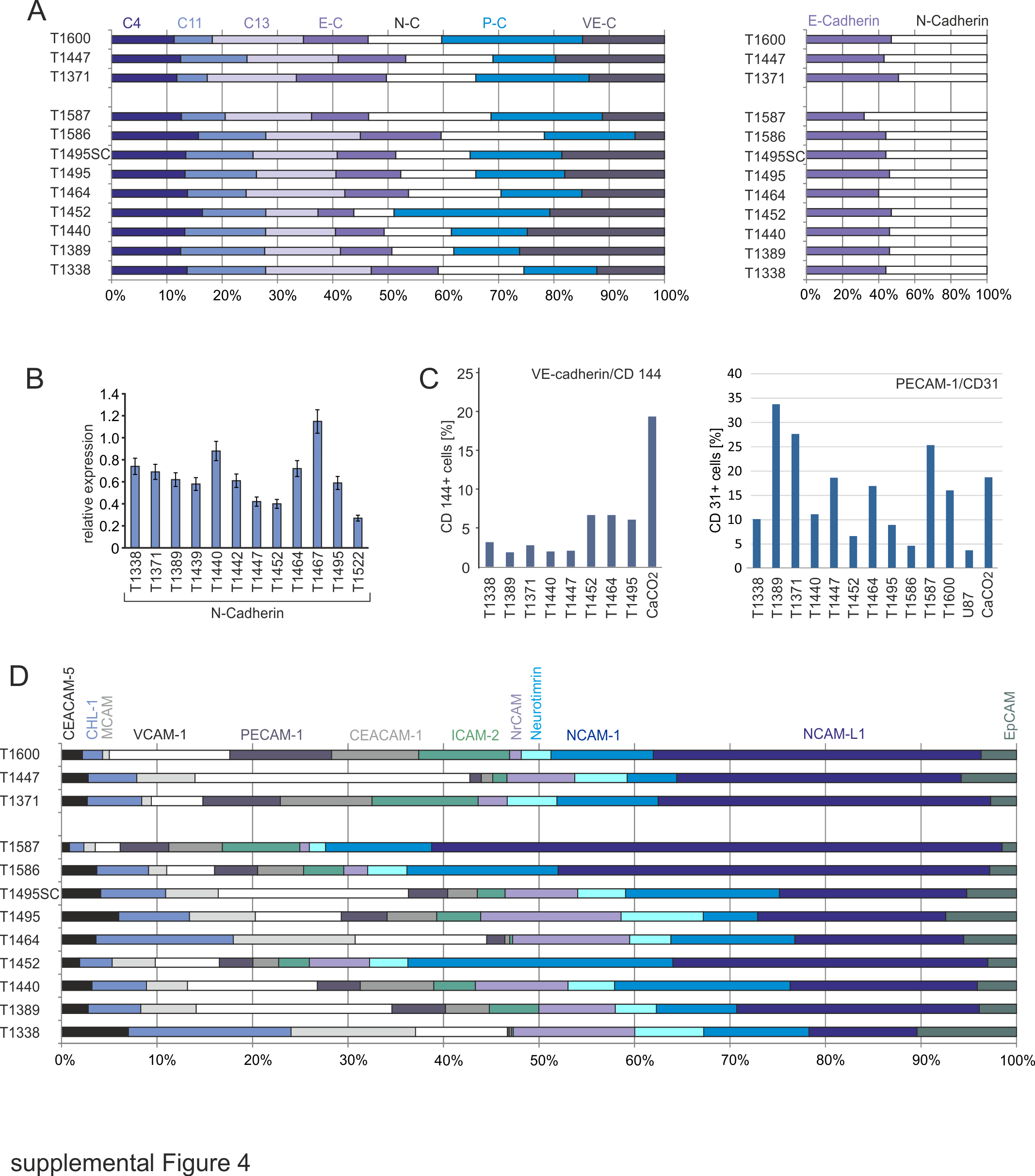

Supplement: S4 Fig — (A) Comparison of the relative amounts of Cadherins expressed in the SLGC lines indicated (data from proteome arrays). Based on the assumption that the antibodies spotted on the filters of the proteome arrays possessed similar KDs, the relative expression of the cadherins was calculated. The sum of the signals of all cadherins was arbitrary set at 100%. The bars indicate to what percentage the individual cadherins contribute to this expression. C4, C11, and C13, cadherins 4, 11 and 13; E-C, E-cadherin; N-C, N-cadherin; P-C, placental cadherin; VE-C, VE-(vascular endothelial) cadherin/CD144. (B) Quantification of Western blot analyzes investigating the expression of N-cadherin in the SLGC lines indicated. Bars represent the mean expression normalized against Actin, whiskers represent the variation between technical replicates. (C) Similar experiment as in Fig 5 using biological replicates of the various SLGC lines (flow cytometry analysis). The established cell lines U87 (glioblastoma) and/or CaCo2 (colon carcinoma), which lack stemness were used as references. The antibodies bind to the VE-cadherin/CD144 and PECAM-1 (platelet endothelial cell adhesion molecule -1)/CD31, respectively. Bars depict the percentage of positive cells determined in one representative experiment. (D) Comparison of the relative amounts of CAMs expressed in the SLGC lines indicated (data from proteome arrays). The sum of the signals of all CAMs was arbitrary set at 100%. The bars indicate to what percentage the individual CAMs contribute to the total CAM expression. Cea-CAM-5 or -1, Carcinoembryonic antigen-related cell adhesion molecule -5 or -1; CHL-1, cell adhesion molecule L1-like; N-CAM, nerve cell adhesion molecule; VCAM, Vascular cell adhesion protein 1; PECAM, platelet endothelial cell adhesion molecule; ICAM-2, intercellular adhesion molecule 2; Neurotrimin, GPI-anchored cell adhesion molecule that might promote neurite outgrowth; NCAM-1, neural adhesion molecule-1; NCAM-L1, neural c [file pone.0291368.s004.tif]

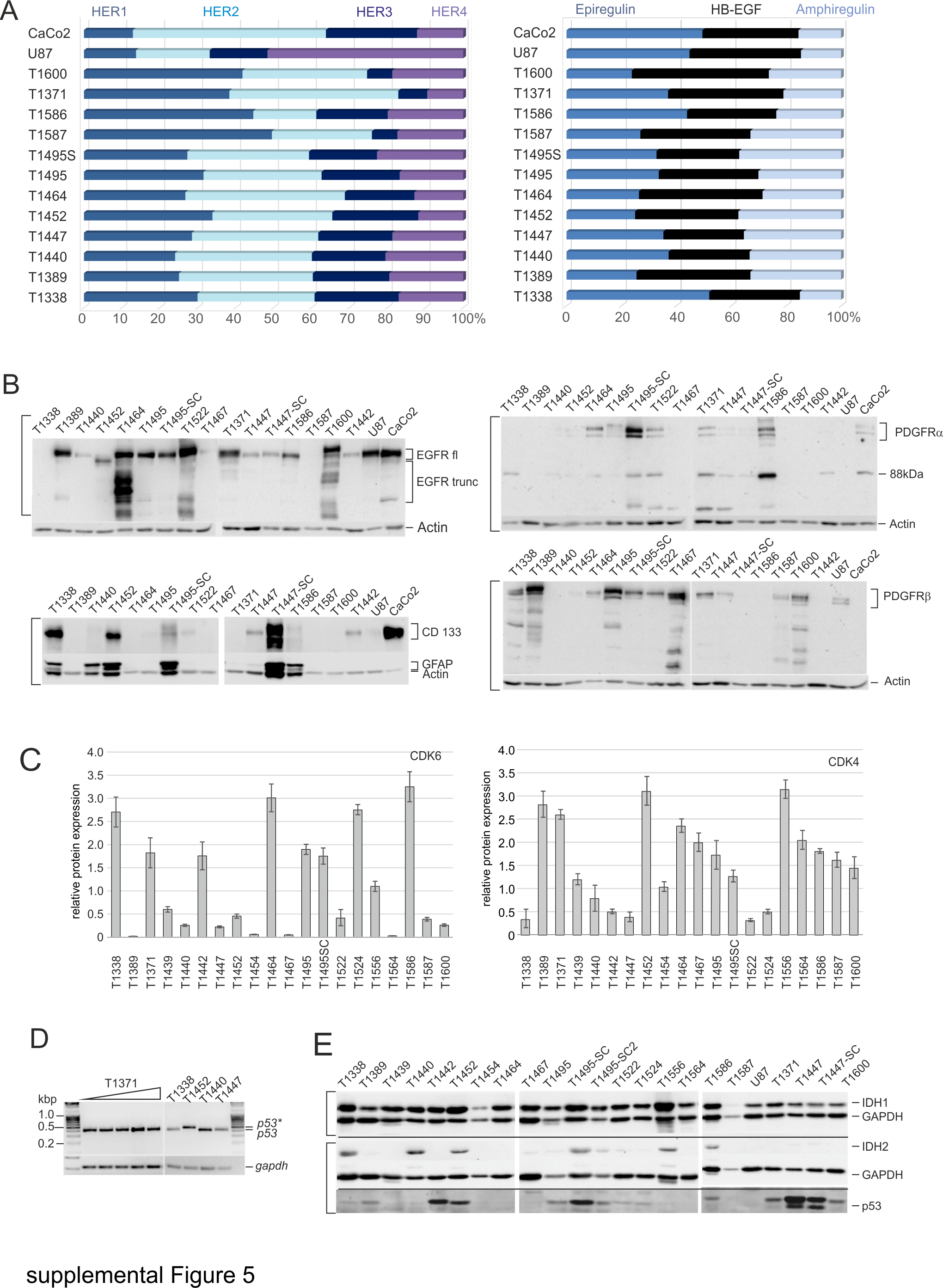

Supplement: S5 Fig — (A) Left: Comparison of the relative amounts of members of the EGF-receptor/HER family (data from proteome arrays). Based on the assumption that the antibodies spotted on the filters of the proteome arrays possessed similar KDs, the relative expression was calculated. The sum of the signals from the four receptors was arbitrary set at 100%. The bars indicate to what percentage the individual HERs contribute to this expression. Right: similar comparison considering Epiregulin, HB-EGF, Amphiregulin, all of which may activate EGFR/HER1. (B) Western blot analysis of the expression of the proteins indicated in the blots. Upper panel of left: The full-length(fl) EGF (epidermal growth factor) receptor and truncated EGFR proteins are shown. Right: Similar blots using antibodies against the PDGF (platelet-derived growth factor) receptors α and β. Lower panel on left: comparison of GFAP and CD133 expression. (C) Relative expression of CDK4 and CDK6: expression was normalized against the corresponding Actin signals. The mean values of three independent biological replicates are shown; whiskers indicate variations. (D) RT-PCR analysis of p53 transcripts. p53* indicates that the PCR product is longer than usual due to a mutation in a splice site. The triangle indicates increasing passages (p2, p5, p9, p26, p35); gapdh served as a reference gene. (E) Western blot analysis of the expression of IDH1 (IDH1: cytoplasmic, 46.7 kDa), IDH2 (IDH2: mitochondrial isoform 1: 50.9 kDa, isoform 2: 45.2 kDa) and p53 in the SLGC lines indicated. The loading control GAPDH is shown below the respective blots. Brackets indicate that the analyses were performed using the same nitrocellulose filter.—GAPDH, glycerol aldehyde-3-phosphate dehydrogenase. (TIF) [file pone.0291368.s005.tif]

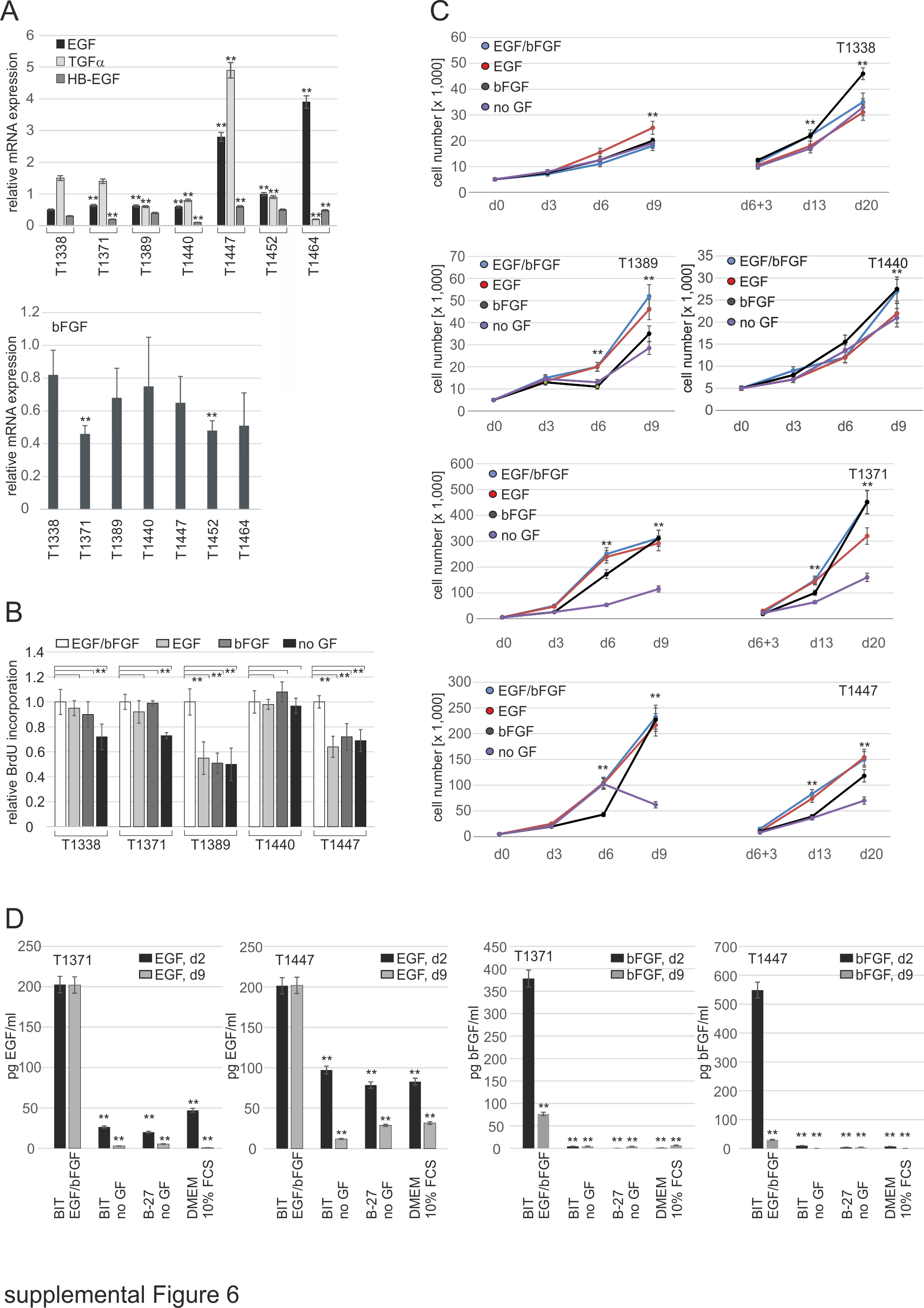

Supplement: S6 Fig — (A) Expression (RT-PCR analysis) of mRNAs coding for EGF (epidermal growth factor), TGFα (transforming growth factor α), HB-EGF (Heparin-binding EGF-like growth factor), and bFGF (basic fibroblast growth factor). Bars depict the mean of a minimum of three replicates, whiskers the standard deviation. Significant differences relative to the expression in the T1338 cell line are indicated (**, p<0.005; ANOVA). (B) Effects of growth factor depletion on proliferation (BrdU ELISA). The combination of growth factors added into the medium is indicated by distinct levels of gray(white: EGF/bFGF; light grey: EGF; dark grey: bFGF; black: no growth factor added). The bars depict mean values and standard deviations. Significant reduction of BrdU incorporation is indicated by p-values (**, p<0.005). (C) Growth curves of the SLGC lines indicated were performed in the presence of EGF (red line), bFGF (black line) or both (blue line), or in the absence of growth factors (violet line). After six days (d6) cells were re-plated for studies with extended incubation times. Values for d9 were determined from both the original and the replated cultures. Significant differences between the growth curves at specific time points are indicated by p-values (*, p<0.05 and **, p<0.005; ANOVA). (D) Growth factor ELISA. The amounts of EGF and bFGF present in T1371 and T1447 cultures were determined at days d2 and d9 after plating. The assays were performed in DMEM/Ham’s F12 containing fetal calf serum (10% FCS) or the serum supplements BIT (bovine albumin, insulin, and transferrin) and B27, respectively. The presence of exogenous growth factors is indicated by EGF/bFGF. Each assay encompassed a minimum of four replicates. Significant differences are indicated (**, p<0.005). (TIF) [file pone.0291368.s006.tif]

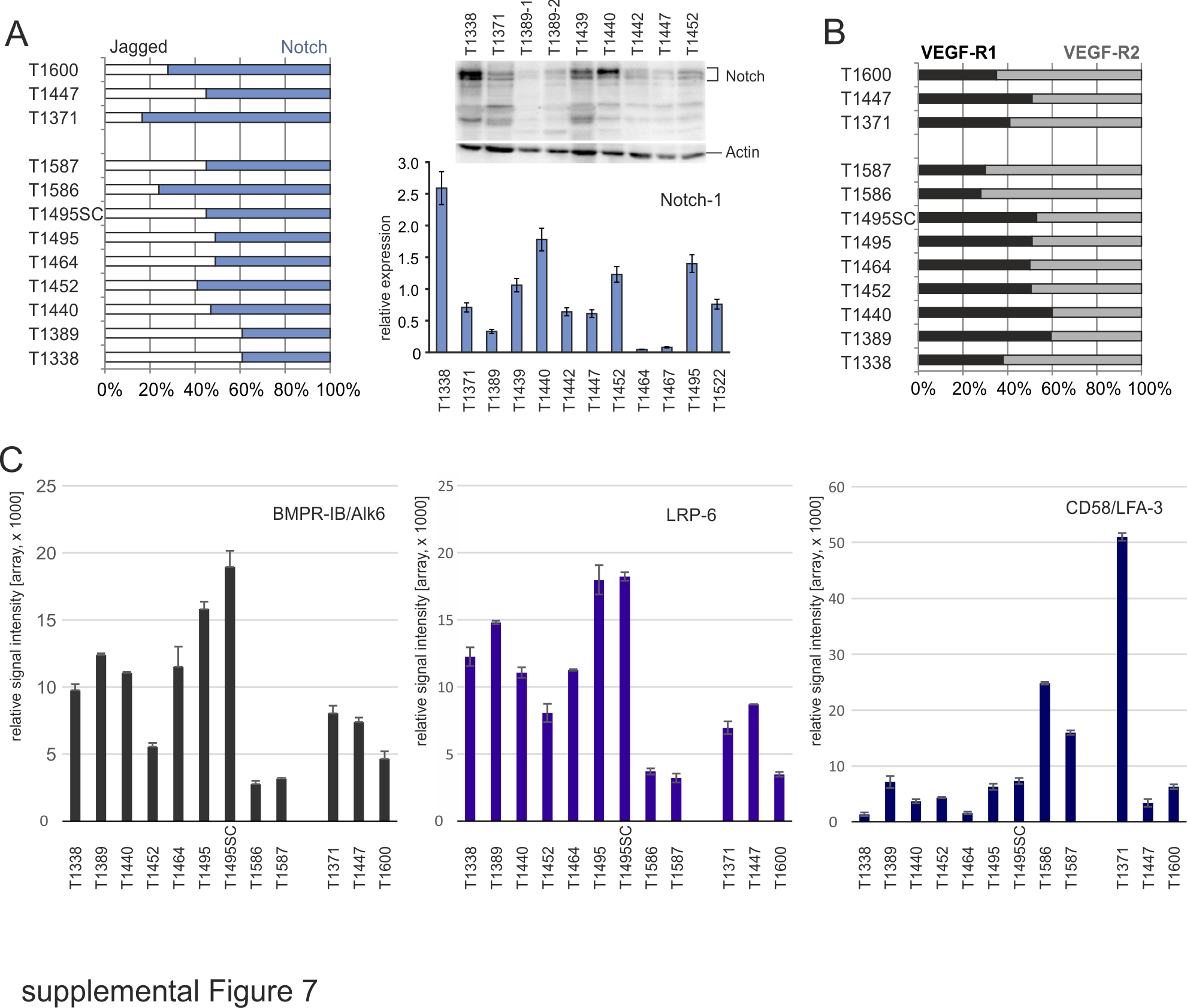

Supplement: S7 Fig — (A) Expression of Notch and Notch-ligand–(Left): Comparison of the relative expression of Notch and the Notch ligand Jagged in the SLGC lines indicated (data from proteome arrays). Right: Western blot analysis of Notch-1 expression in the SLGC lines indicated. The loading control Actin is shown below the blot. The bar graph below depicts the mean values of Notch-1 expression (normalized against Actin) and their variation in technical replicates of the SLGC lines indicated. (B) Relative expression of VEGF-R1 and–R2. Comparison of the relative expression of the two receptors in the SLGC lines indicated (data from proteome arrays). (C) Relative expression of the BMP (bone morphogenetic protein) receptor IB/Alk6, LRP (low density lipoprotein-related protein 1) and CD58/LFA-3 (lymphocyte-associated antigen-3) in the SLGC lines indicated (proteome array data). The bars depict the mean from two independent arrays, the whiskers show the variation. (TIF) [file pone.0291368.s007.tif]

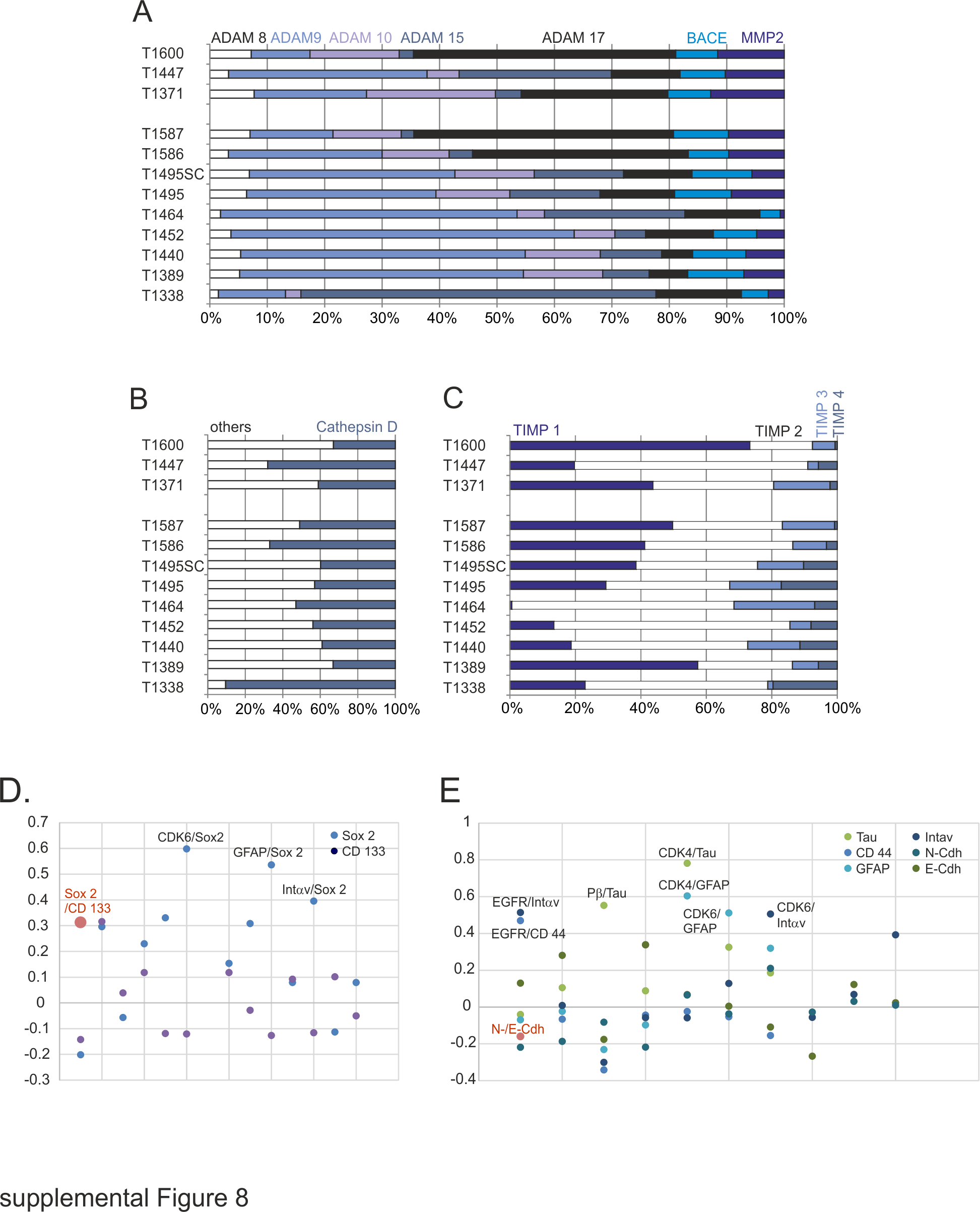

Supplement: S8 Fig — (A) Comparison of the relative amounts of ADAM (a disintegrin and metalloprotease domain) proteases as well as BACE (beta-secretase) and MMP2 (matrix metallopeptidase 2) in the SLGC lines indicated (data from proteome array). Based on the assumption that the antibodies spotted on the filters of the proteome array possessed similar KDs, the relative expression of the proteases was calculated. The sum of the signals of all proteases was arbitrary set at 100%. The bars indicate to what percentage the individual proteases contribute to this expression. (B) Similar assay as in (A). The relative Cathepsin D levels were compared to the relative expression of all other proteases, depicted in panel (A). (C) Similar analysis as in (A) comparing the relative expression of the protease inhibitors TIMP (tissue inhibitor of metalloproteinases) 1, 2, 3 and 4.–Parts D and E graphically summarize some of the correlation data shown in Figs 8 and 9. In (B) the coefficients are indicated on the y-axis, highlighting expression levels with a positive correlation to the Sox2 (blue) or CD133 (violet) dots. In (C) the Pearson correlation coefficients were calculated relative to the levels of the neural proteins Tau and GFAP, as well as the hyaluronan receptor CD44, the integrin αv, and the N- and E-cadherin, respectively. In all cases, the expression levels that entered the calculations were determined with the same whole cell extracts. The calculations were confirmed with biological replicates. (TIF) [file pone.0291368.s008.tif]

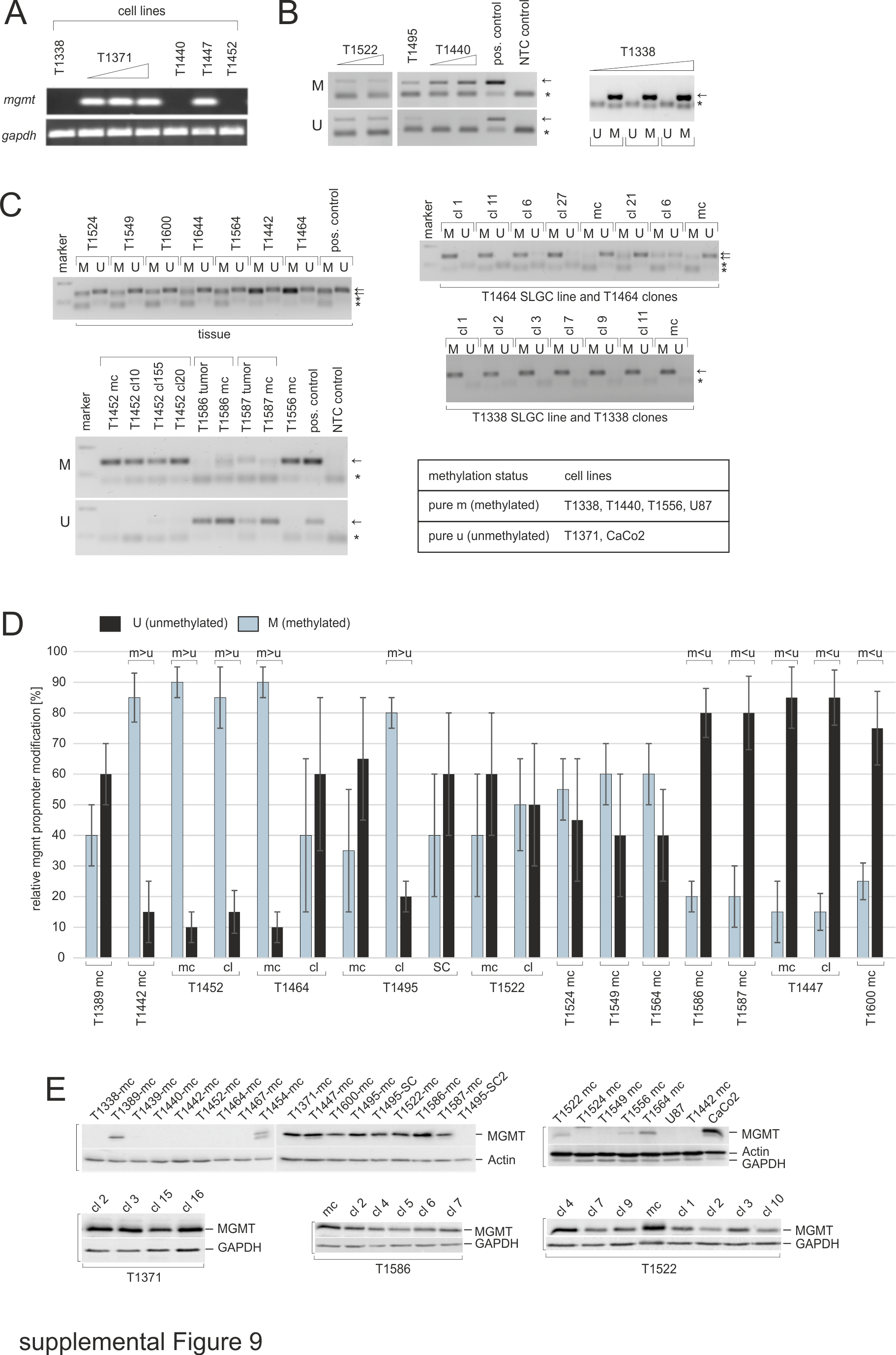

Supplement: S9 Fig — (A) RT-PCR analysis of mgmt mRNA expression; gapdh served as a control. T1371 was studied during passages p2, p6, and p26. (B, C) Examples of methylation-specific PCR (MSP) using gDNA from tumor tissue as well as SLGC lines (mc) or clones (cl) obtained by a limited dilution assay. M (methylated status), PCR using the primers MGMTmeth-F/MGMTmeth-R; U (unmethylated status), PCR using the primers MGMTun/MGMTun-R. Arrows point to the specific PCR products, *, labels unused primers/frequently seen byproducts of <40 bps. The box depicts cell lines with a pure m- or u- status, including SLGCs as well as the non-SLGC GBM line U87 and the colon cancer cell line CaCo2, which served as controls in various experiments. (D) Summary of the quantification of the MSP data; mc/SC, SLGC/SCID lines; cl, clones derived from SLGC lines by means of limited dilution assays.–For mc/SC the mean values ± SEM from a minimum of three biological replicates are shown. For clones, the bars represent the mean values calculated from all clones together, and whiskers indicate the variations between the relative percentages of the PCR products determined for the clones. Altogether, six T1338, four T1371, five T1452, three T1447, six T1464, seven T1522 and five T1586 clones were included in the study. (E) Western blot analysis of the expression of the MGMT protein.–Altogether, six T1338, four T1371, three T1447, six T1464, seven T1522 and five T1586 clones were included in the study. (TIF) [file pone.0291368.s009.tif]
