# Supplementary material for: Molecular signature of stem-like glioma cells (SLGCs) from human glioblastoma and gliosarcoma
Source: PLoS One. 2024 Feb 2;19(2):e0291368. doi: 10.1371/journal.pone.0291368 (PMC10836714; doi:10.1371/journal.pone.0291368)
Supplement: S1 Table — (DOCX) [file pone.0291368.s010.docx]

**Supplemental Table** : **mutations and protein expression**

| Tumor type / Cell line | PTEN  protein | Tp53  sequence | IDH1  sequence | IDH2  protein | highly expressed RTK |
| --- | --- | --- | --- | --- | --- |
| GBM – T1338 | - | WT/mutant | WT | + | (Pβ) |
| T1338 cl1 | - | WT | WT | + | - |
| GBM – T1389 | - | mutant* | Silent mutation | - | Pβ |
| T1389 SC | - | n.d. | n.d. | - | Pβ |
| GBM – T1439 | + | WT | n.t. | - | Pα, Pβ, M |
| GBM – T1440 | +/loss* | WT | WT | + | - |
| T1440 cl4 | +/- | WT | n.t. | n.d. | - |
| T1440 cl5 | + | WT | n.t. | n.d. | - |
| T1440 cl8 | - | WT | WT | + | - |
| GBM – T1442 | - | N239D | WT | - | - |
| GBM – T1452 | - | Δ3‘ splice site | WT | + | - |
| T1452 cl10 | - | Δ3‘ splice site | WT | + |  |
| GBM – T1454 | + | WT | n.t. | - | Pβ, M |
| GBM – T1464 | + | WT | WT | - | E^§^, (Pα), Pβ, M |
| GBM – T1467 | - | WT | n.t. | (+) | E,Pβ |
| GBM – T1495 | + | R273H (GOF) | WT | (+) | (E),(Pα), Pβ, M |
| T1495 SC | + | R273H (GOF) | n.t. | + | (E),Pα, Pβ, M |
| T1495 SC2 | + | R273H (GOF) | n.t. | + | (E),Pα, Pβ, M |
| GBM – T1522 | - | WT | WT | (+) | E, (Pα), M |
| GBM – T1524 | (+) | n.d. | n.t. | (+) | E,Pα |
| GBM – T1549 | (+) | n.d. | n.t. | (+) | - |
| GBM – T1556 | + | n.d. | n.t. | + | - |
| GBM – T1564 | + | n.d. | n.t. | - | Pα |
| GBM – T1586 | - | WT | WT | + | Pα, M |
| GBM – T1587 | + | WT | WT | - | - |
|  |  |  |  |  |  |
| GS – T1371 | - | R175H (GOF) | WT | - | E, (Pα), Pβ, M |
| T1371 cl16 | - | R175H (GOF) | WT | - | (Pα), M |
| GS* – T1447 | - | R248W (GOF) | WT | (+) | (Pβ),M |
| T1447 SC | - | R248W (GOF) | n.t | (+) | (Pβ),M |
| T1447 cl4 | - | R248W (GOF) | WT | (+) | (Pβ),M |
| GS – T1600 | + | R175H (GOF) | WT | - | E^§^,Pβ |

GBM; glioblastoma multiforme; GS, gliosarcoma; GS*, recurrent gliosarcoma; suffix “SC”, indicates that the cell lines was established from an orthotopic tumor grown in a SCID mouse (SC2, was derived from xenotransplanted T1495-SC); PTEN, *Phosphatase and tensin homolog;*  +/loss*, PTEN status in T1440 subpopulations is +/+, +/- or -/-; T1389 mutant*, subpopulations with mixed Tp53 status in exons 5 and 6; T1338 WT*, a subpopulation of T1338 cells is heterozygote for Tp53 mutation; RTK, receptor tyrosine kinase, IDH, isocitrate dehydrogenase; E, [EGFR] epidermal growth factor receptor; E^§^, amplification of truncated EGFR; Pα, Pβ, platelet-derived growth factor [PDGF] receptors α and β; MERTK**, tyrosine protein kinase** Mer - **n.t., not tested.**
