# Supplementary material for: Molecular signature of stem-like glioma cells (SLGCs) from human glioblastoma and gliosarcoma
Source: PLoS One. 2024 Feb 2;19(2):e0291368. doi: 10.1371/journal.pone.0291368 (PMC10836714; doi:10.1371/journal.pone.0291368)
Supplement: S1 Raw images — (PDF) [file pone.0291368.s011.pdf]

S1_ raw_data:

uncropped gels and western blots

- Images of raw data are organized according to their appearance in the manuscript or the supplementary.

- Agarose gels were detected with the ChemiDoc XRS using 5 increasing exposure times. Gel images were exported using the software MicroWin 2000 and the function "export raw data/TIF". One suitable exposure time was selected to show the raw data. Most gels are shown after "color inversion".

- Western blots were detected with the ChemiDoc XRS using 15 – 20 increasing exposure times: Again images were exported using the software MicroWin 2000 and the function "export raw data/TIF". The most suitable exposure time (good signal-noise ratio and contrast) was selected.

Fig. 2A → FABP7

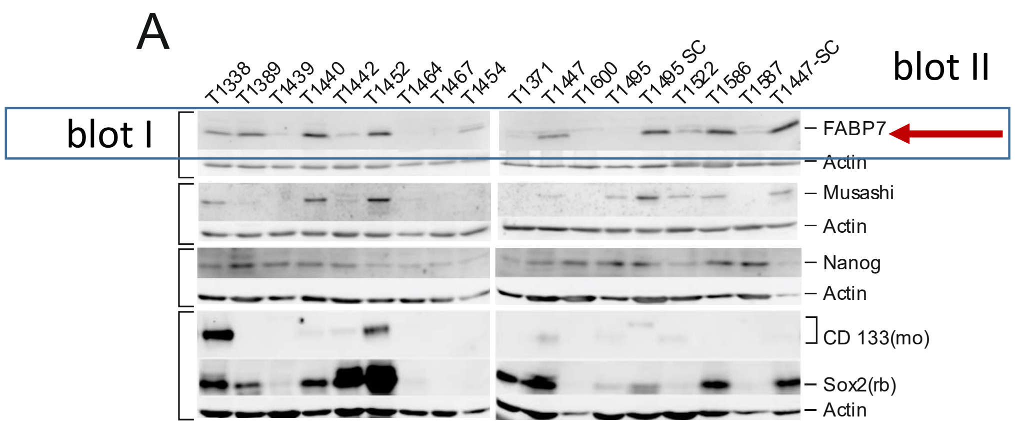
A
T1338
T1389
T1439
T1440
T1442
T1452
T1464
T1467
T1454
T1371
T1447
T1600
T1495
T1495 SC
T1522
T1586
T1587
T1447-SC
blot II
blot I
FABP7
Actin
Musashi
Actin
Nanog
Actin
CD 133(mo)
Sox2(rb)
Actin

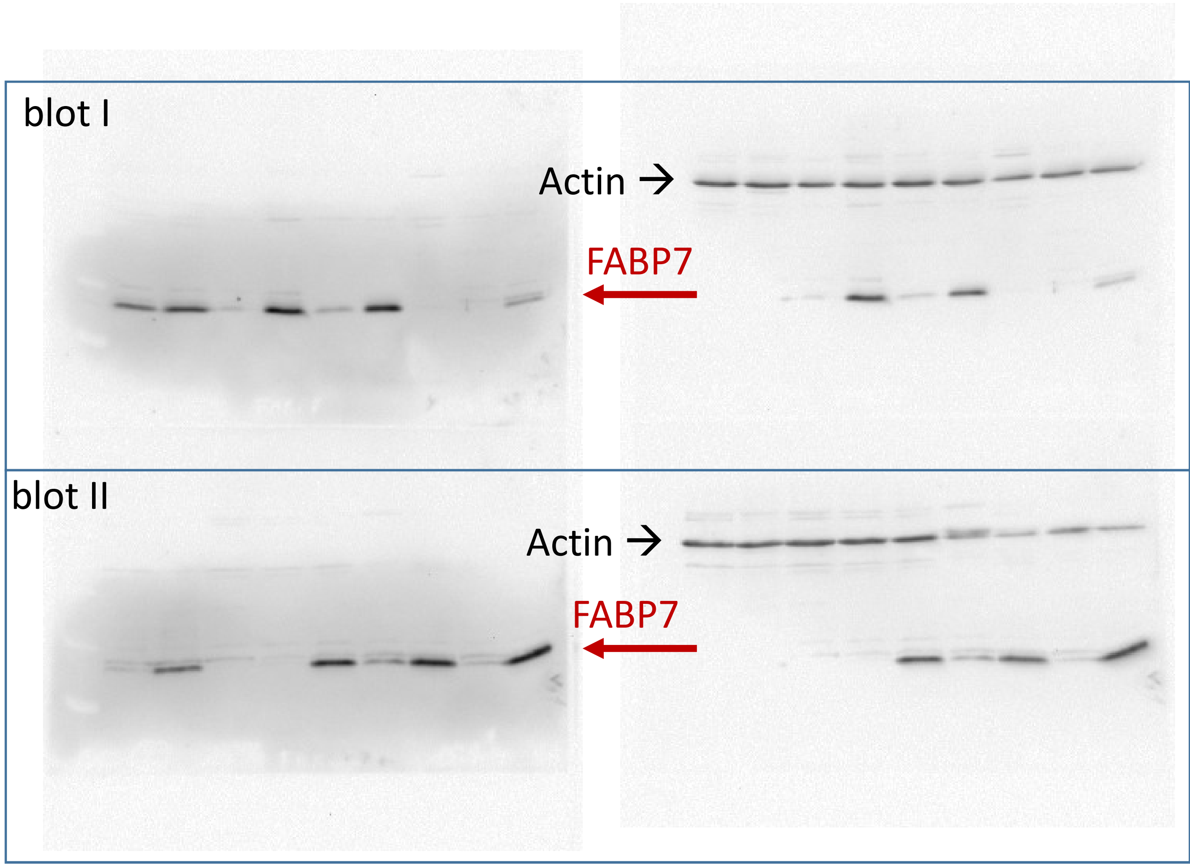
blot I
Actin →
FABP7
blot II
Actin →
FABP7

Fig. 2A → Musashi

A

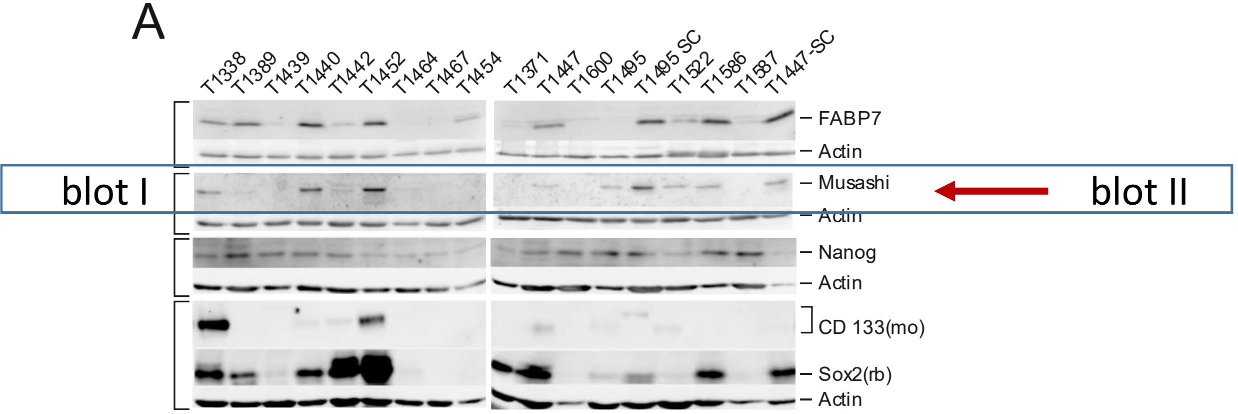

blot I

blot II

blot I

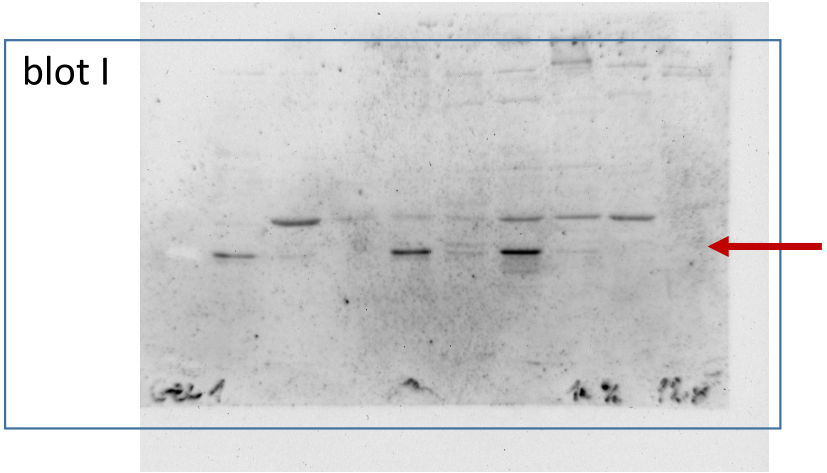

blot II

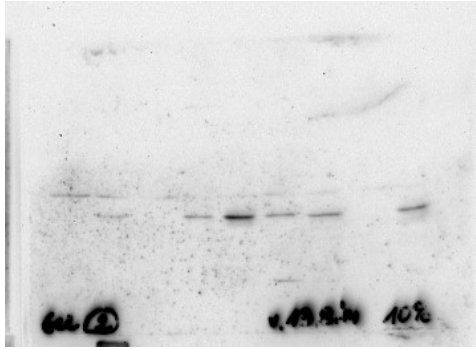

Fig. 2A → Nanog

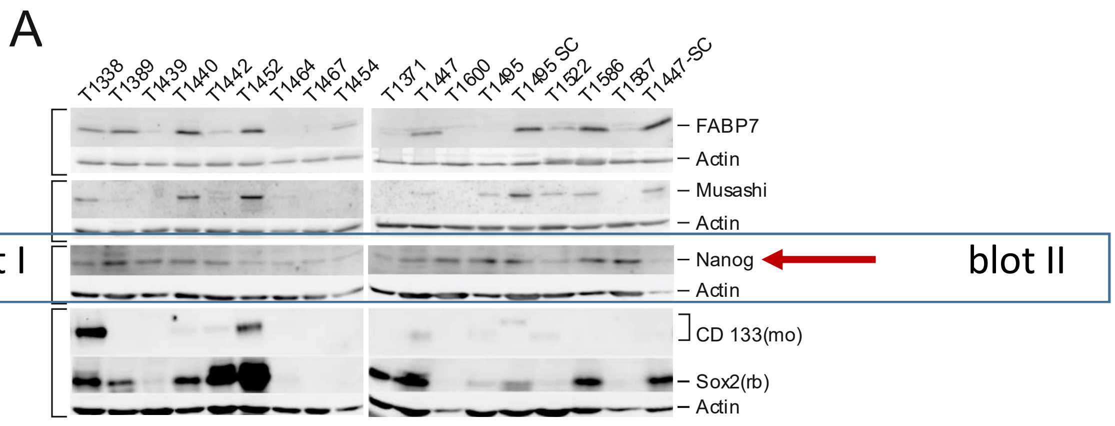
A
T1338
T1389
T1439
T1440
T1442
T1452
T1464
T1467
T1454
T1371
T1447
T1600
T1495
T1495 SC
T1522
T1586
T1587
T1447-SC
FABP7
Actin
Musashi
Actin
Nanog
Actin
CD 133(mo)
Sox2(rb)
Actin
blot I
blot II

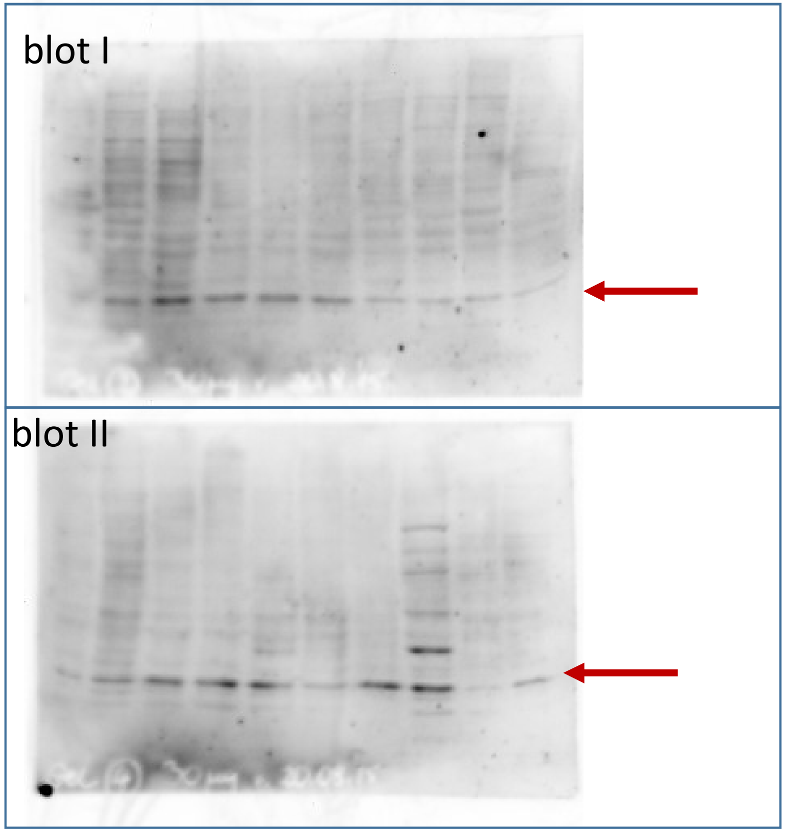
blot I
blot II

Fig. 2A → CD133

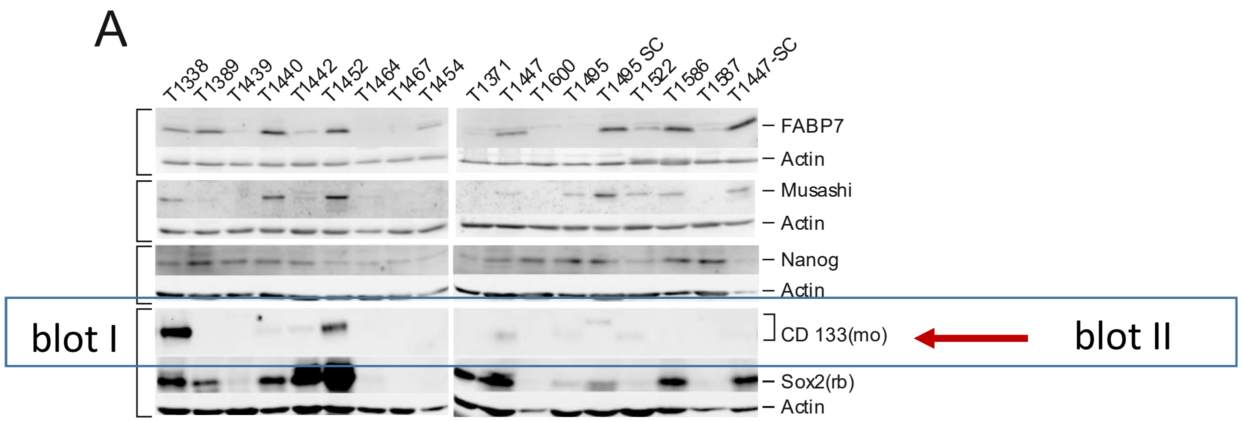
A
T1338
T1389
T1439
T1440
T1442
T1452
T1464
T1467
T1454
T1371
T1447
T1600
T1495
T1495 SC
T1522
T1586
T1587
T1447-SC
FABP7
Actin
Musashi
Actin
Nanog
Actin
CD 133(mo)
Sox2(rb)
Actin
blot I
blot II

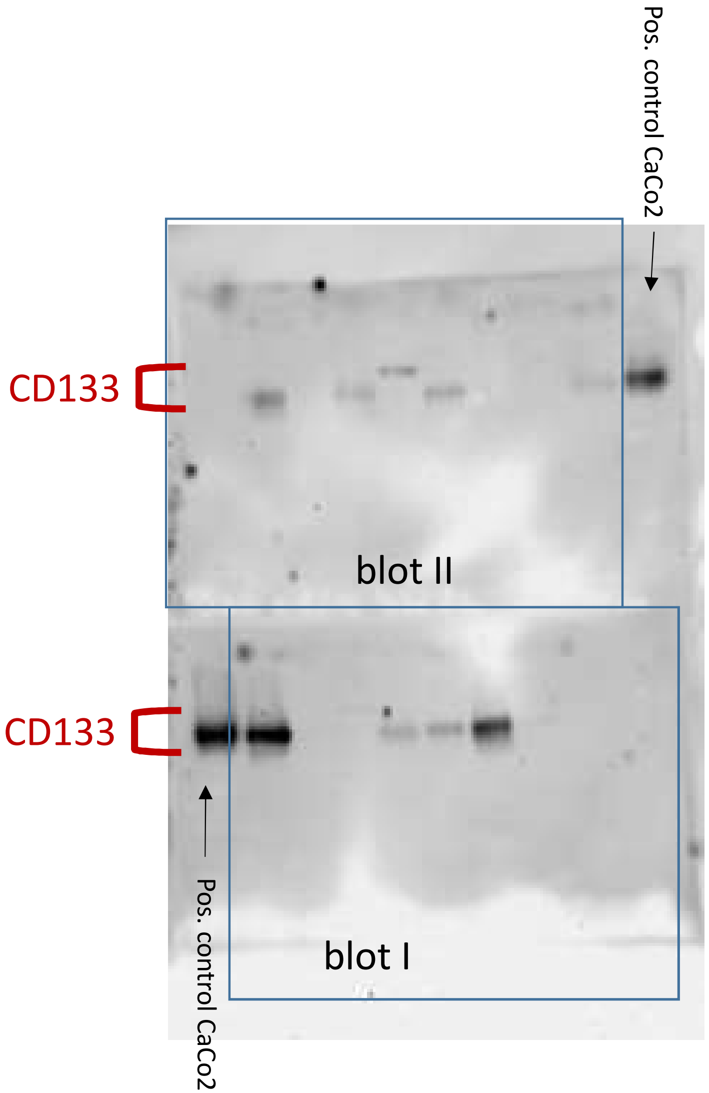
Pos. control CaCo2
CD133
blot II
CD133
Pos. control CaCo2
blot I

Fig. 2A → Sox2

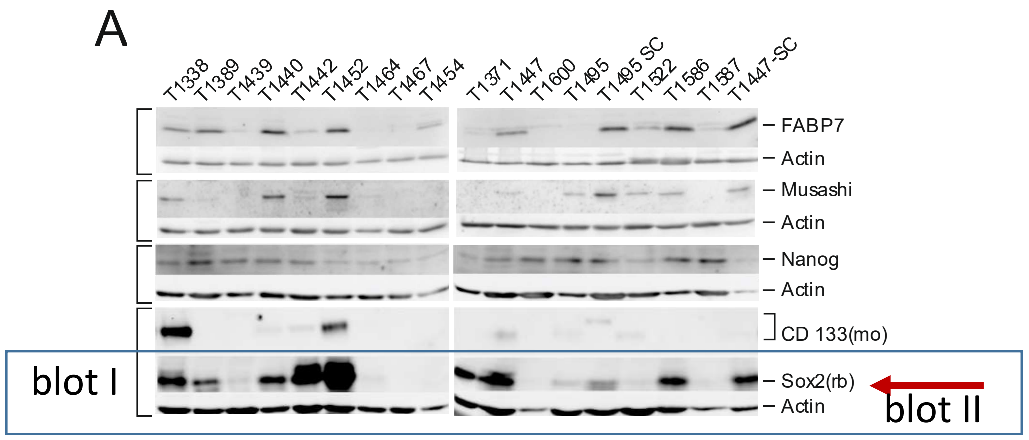
A
T1338
T1389
T1439
T1440
T1442
T1452
T1464
T1467
T1454
T1371
T1447
T1600
T1495
T1495 SC
T1522
T1586
T1587
T1447-SC
FABP7
Actin
Musashi
Actin
Nanog
Actin
CD 133(mo)
Sox2(rb)
Actin
blot I
blot II

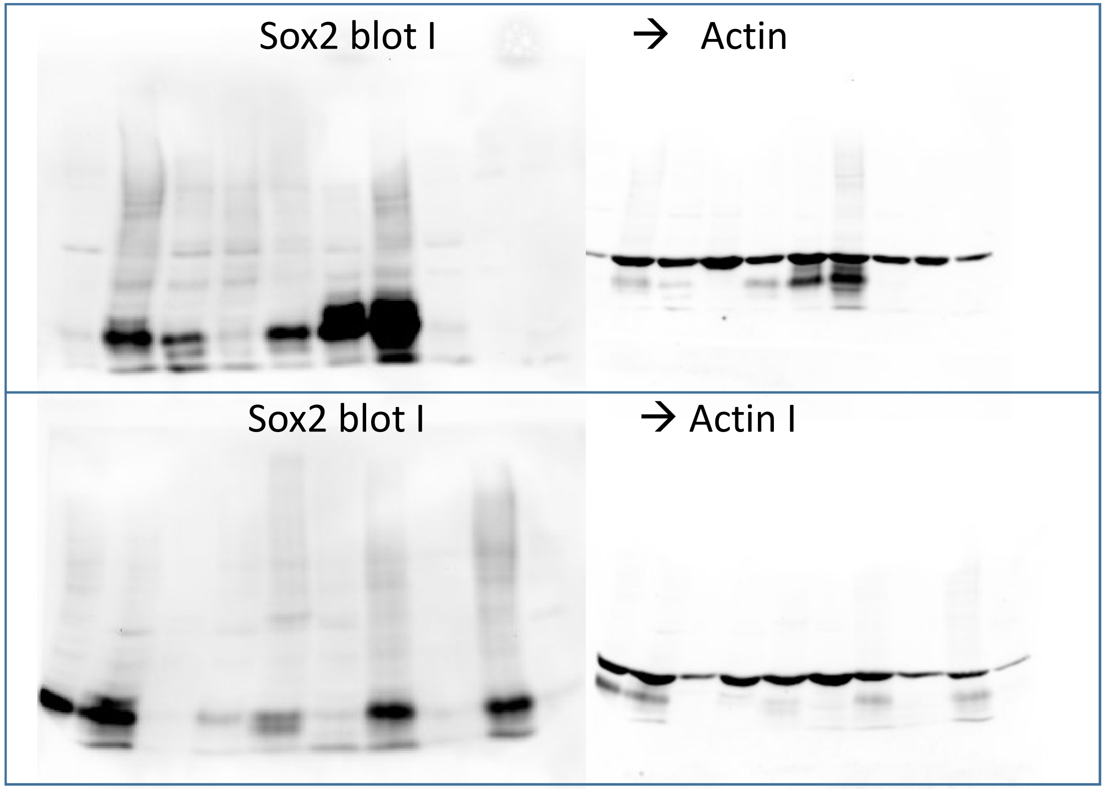
Sox2 blot I
→ Actin
Sox2 blot I
→ Actin I

Fig. 2B → p53

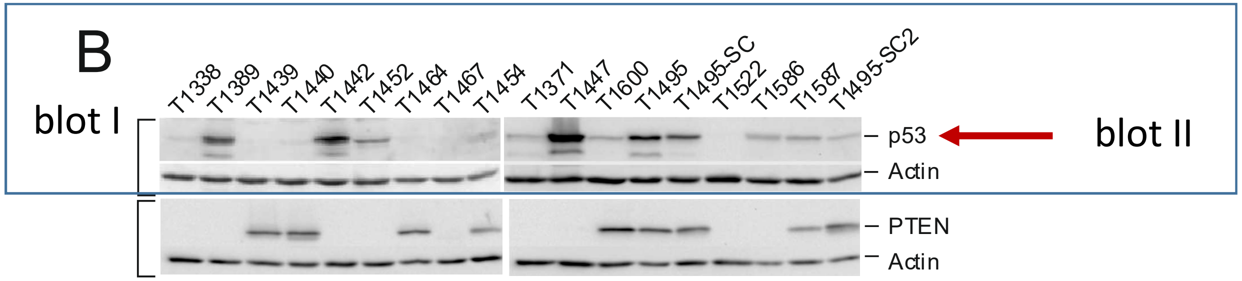
B
blot I
T1338
T1389
T1439
T1440
T1442
T1452
T1464
T1467
T1454
T1371
T1447
T1600
T1495
T1495-SC
T1522
T1586
T1587
T1495-SC2
p53
Actin
PTEN
Actin
blot II

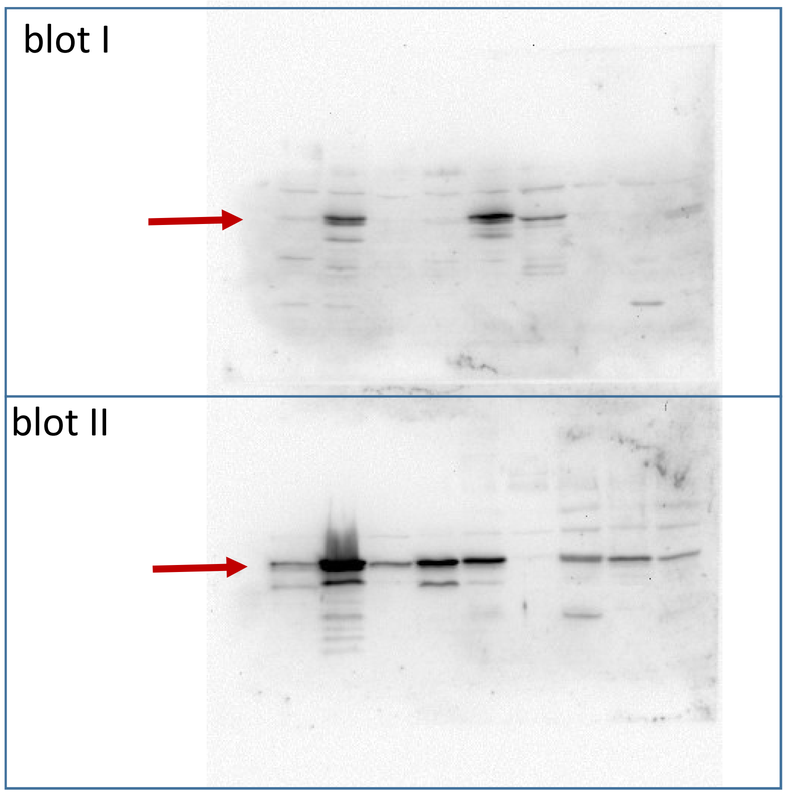
blot I
blot II

Fig. 2B → PTEN

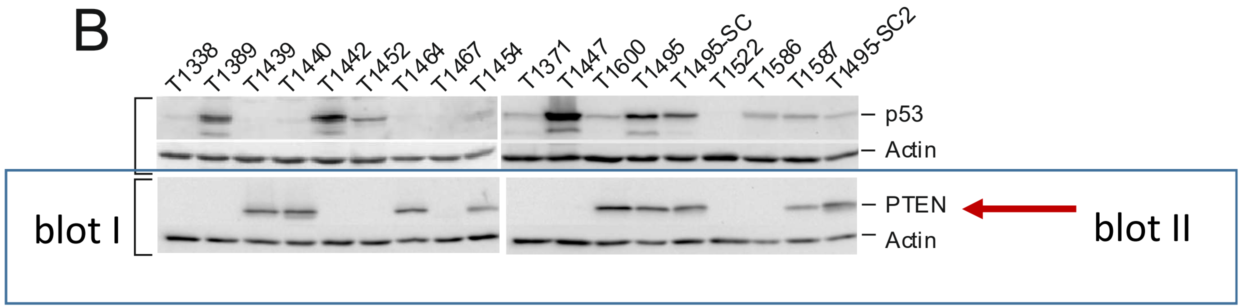
B
T1338
T1389
T1439
T1440
T1442
T1452
T1464
T1467
T1454
T1371
T1447
T1600
T1495
T1495-SC
T1522
T1586
T1587
T1495-SC2
p53
Actin
PTEN
Actin
blot I
blot II

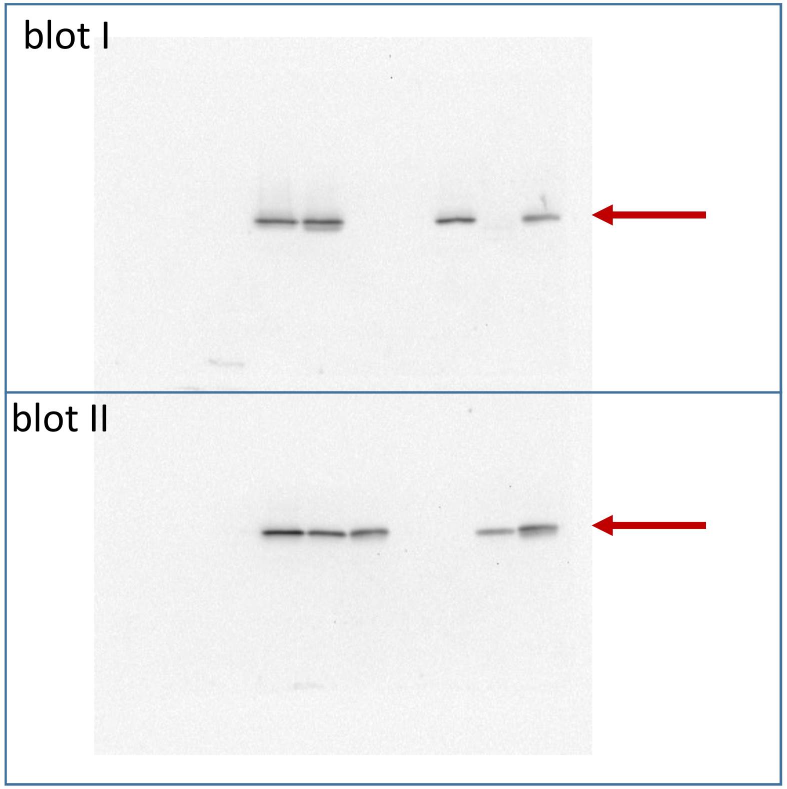
blot I
blot II

Fig. 2C → CDK6

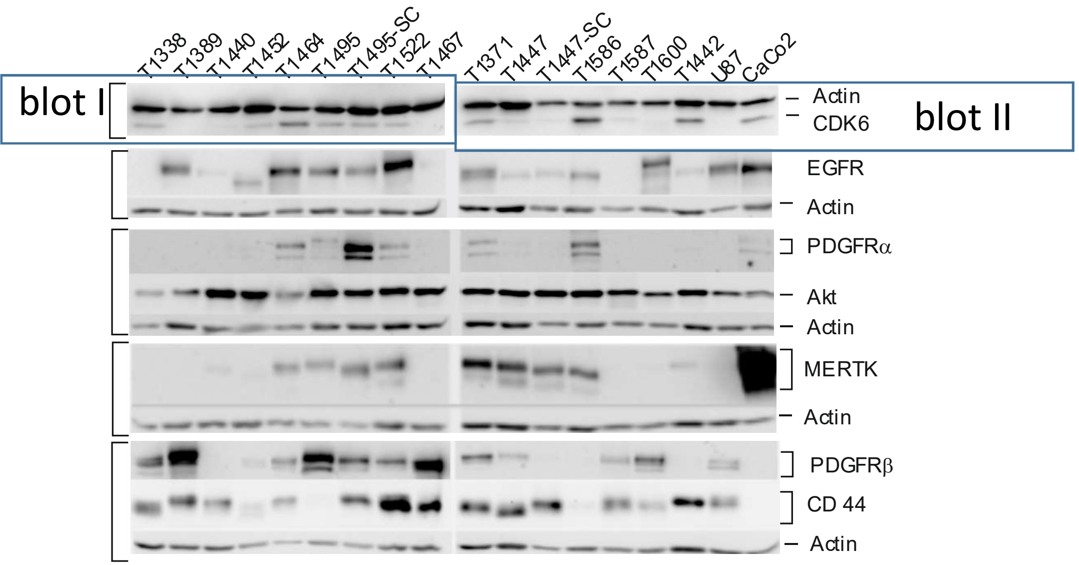
T1338
T1389
T1440
T1452
T1464
T1495
T1495-SC
T1522
T1467
T1371
T1447
T1447-SC
T1586
T1587
T1600
T1442
U87
CaCo2
blot I
blot II
Actin
CDK6
EGFR
Actin
PDGFRα
Akt
Actin
MERTK
Actin
PDGFRβ
CD 44
Actin

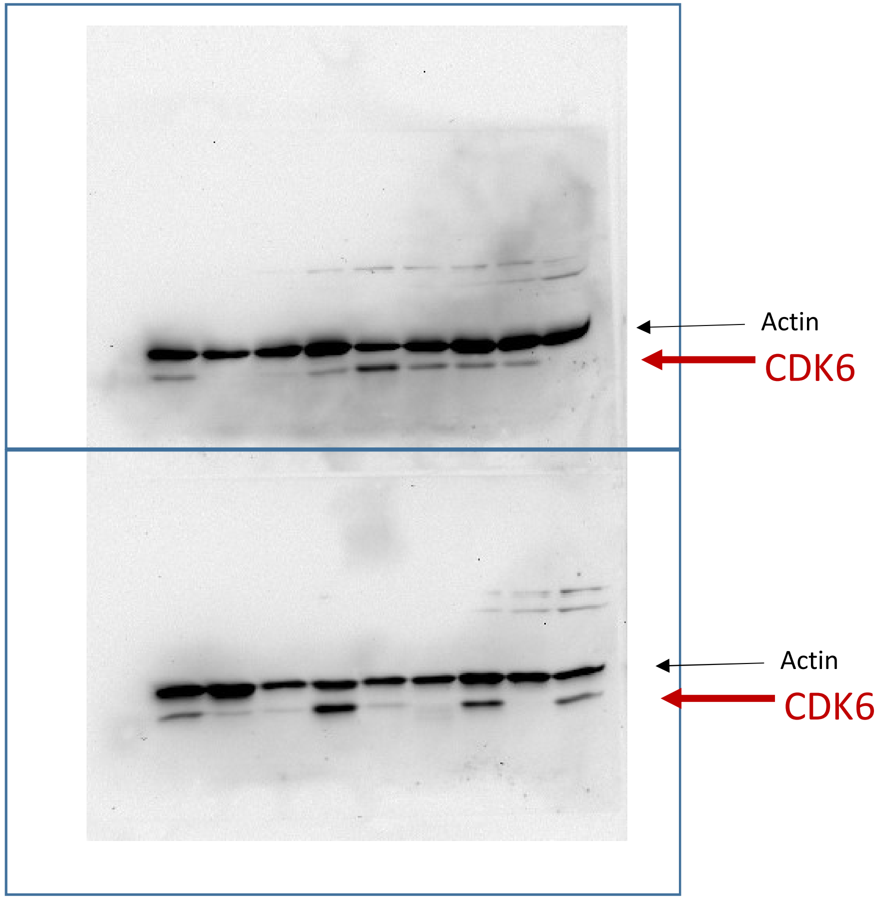
Actin
CDK6
Actin
CDK6

Fig. 2C → EGFR

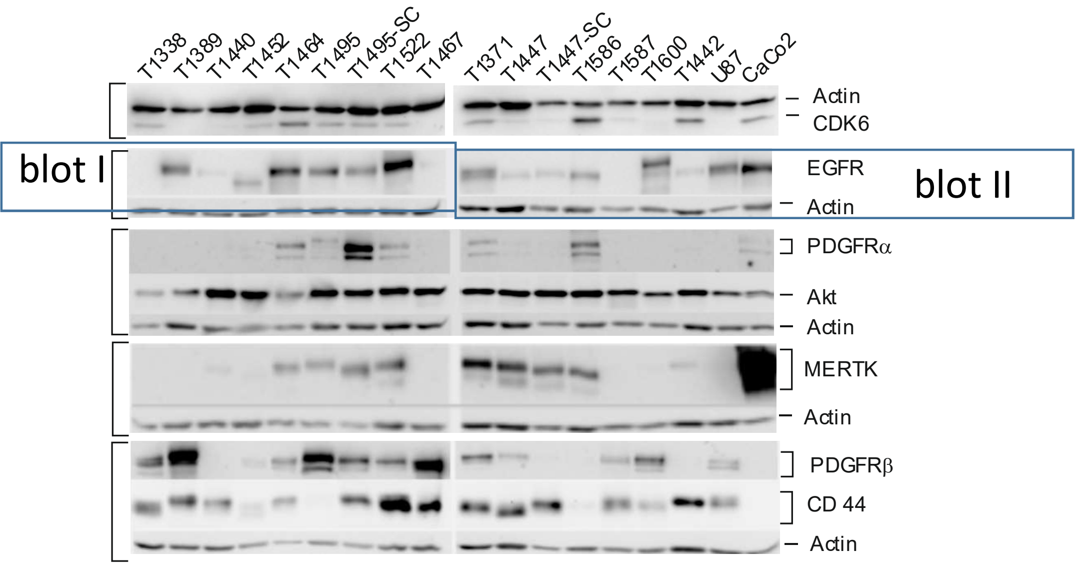

T1338
T1389
T1440
T1452
T1464
T1495
T1495-SC
T1522
T1467
T1371
T1447
T1447-SC
T1586
T1587
T1600
T1442
U87
CaCo2
Actin
CDK6
blot I
EGFR
Actin
blot II
PDGFRα
Akt
Actin
MERTK
Actin
PDGFRβ
CD 44
Actin

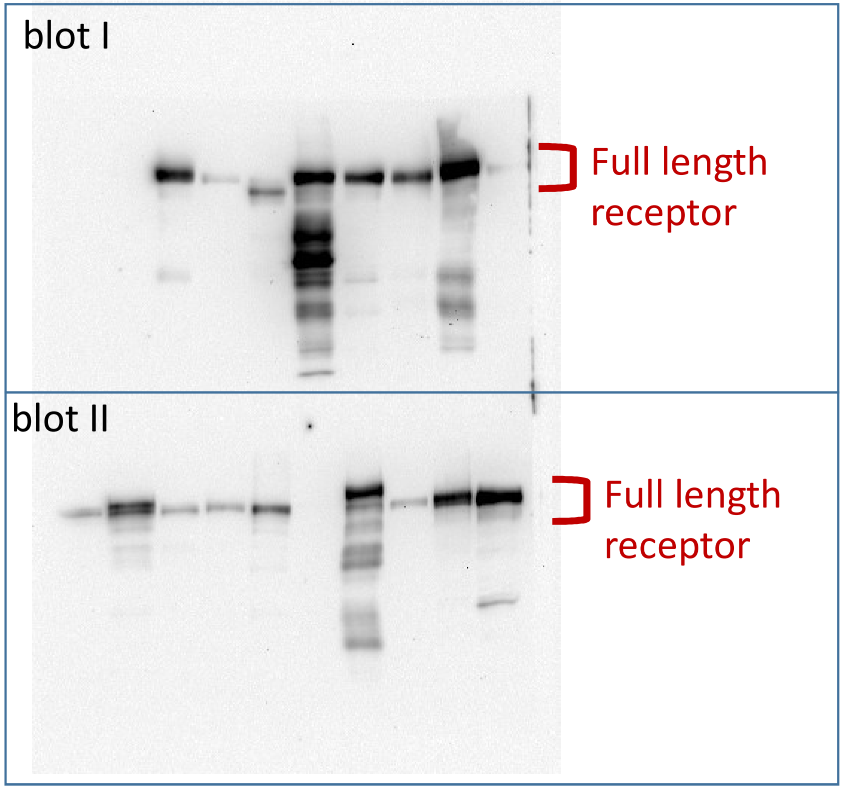

blot I
Full length receptor
blot II
Full length receptor

Fig. 2C → PDGFRα

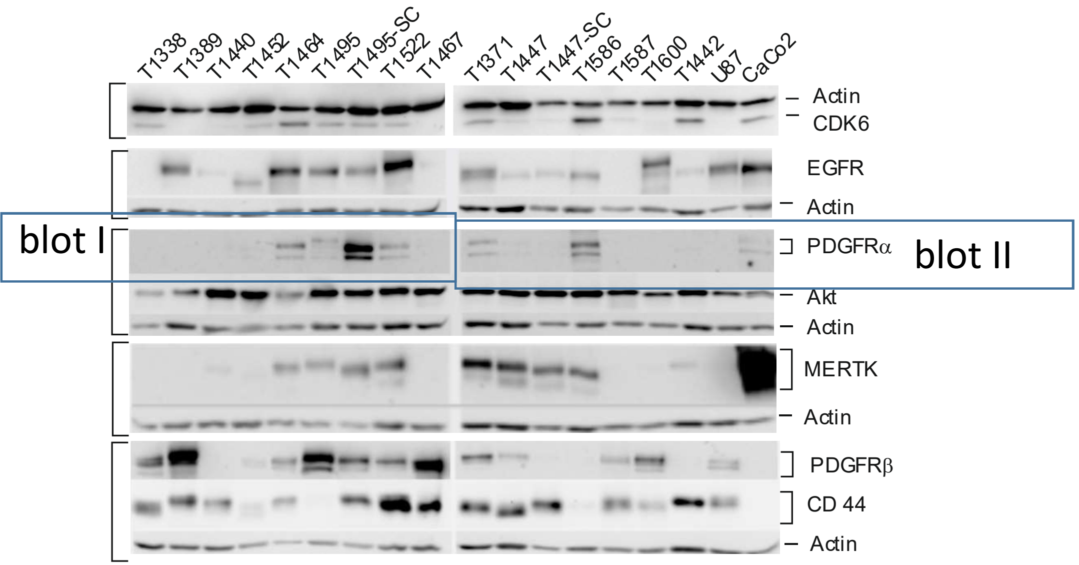

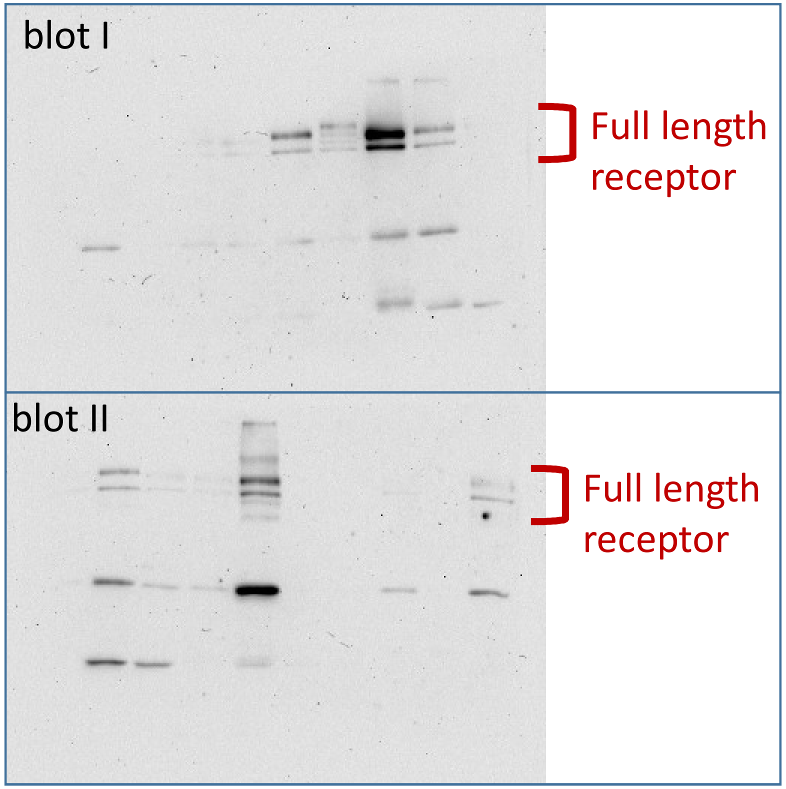

Fig. 2C → AKT

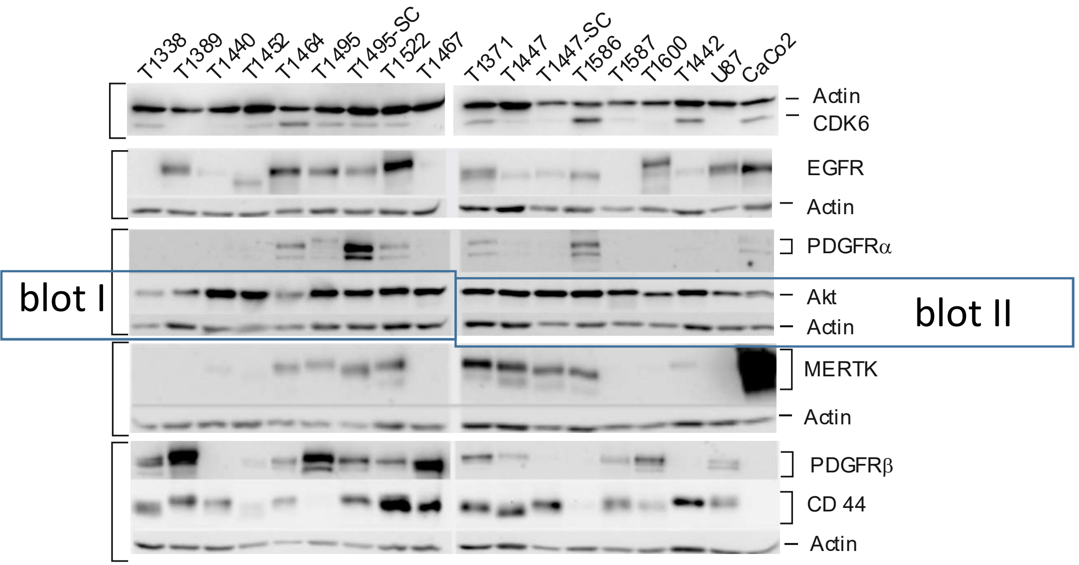
T1338
T1389
T1440
T1452
T1464
T1495
T1495-SC
T1522
T1467
T1371
T1447
T1447-SC
T1586
T1587
T1600
T1442
U87
CaCo2
Actin
CDK6
EGFR
Actin
PDGFRα
Akt
Actin
MERTK
Actin
PDGFRβ
CD 44
Actin
blot I
blot II

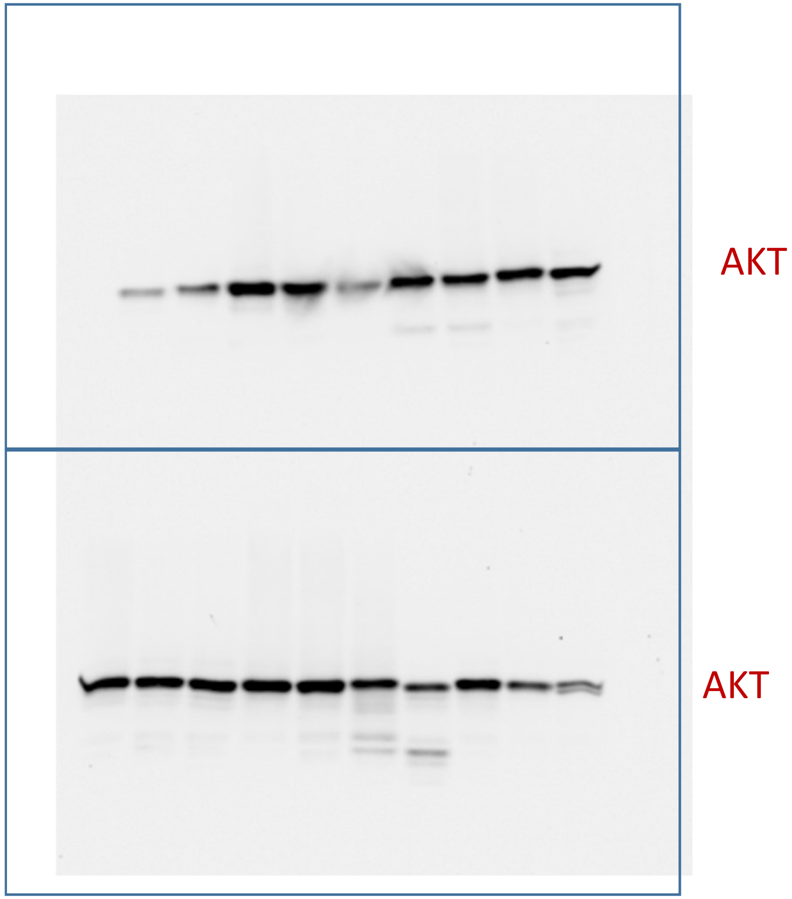
AKT
AKT

Fig. 2C → MERTK

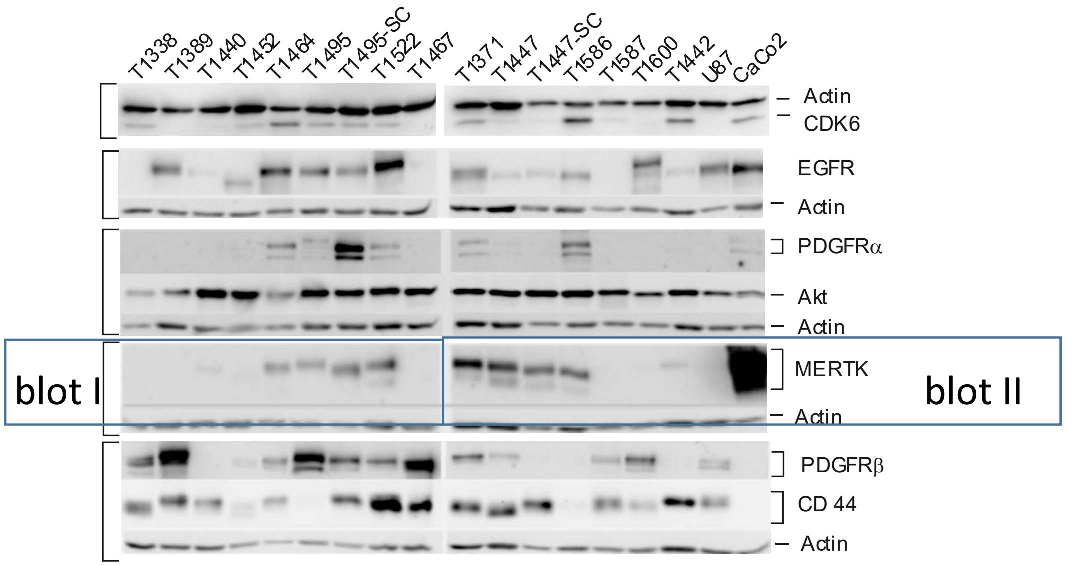
T1338
T1389
T1440
T1452
T1464
T1495
T1495-SC
T1522
T1467
T1371
T1447
T1447-SC
T1586
T1587
T1600
T1442
U87
CaCo2
Actin
CDK6
EGFR
Actin
PDGFRα
Akt
Actin
MERTK
Actin
PDGFRβ
CD 44
Actin
blot I
blot II

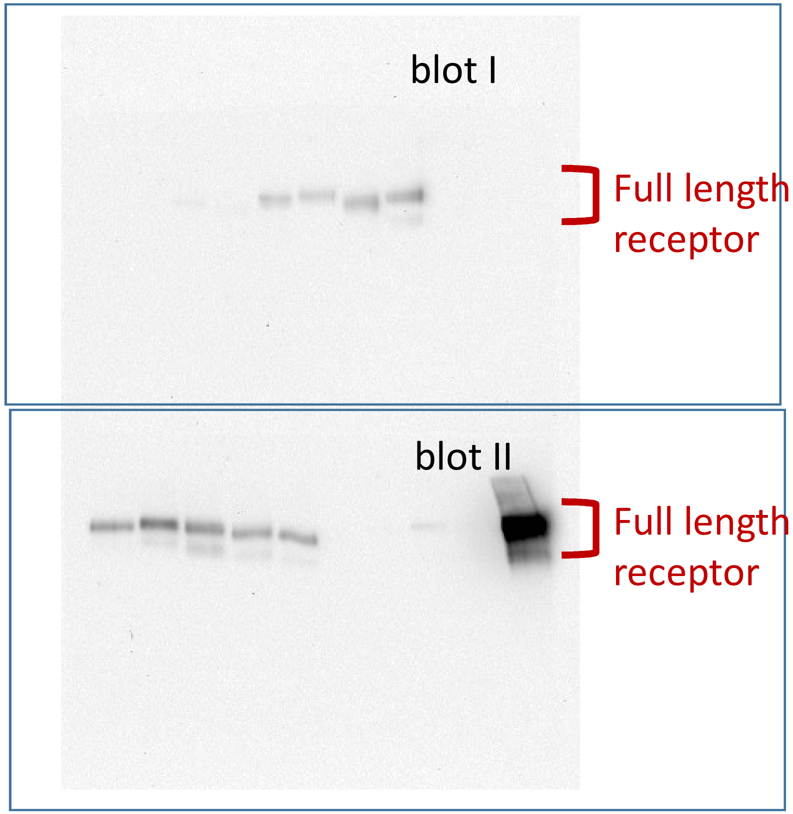
blot I
Full length receptor
blot II
Full length receptor

Fig. 2C → PDGFRβ

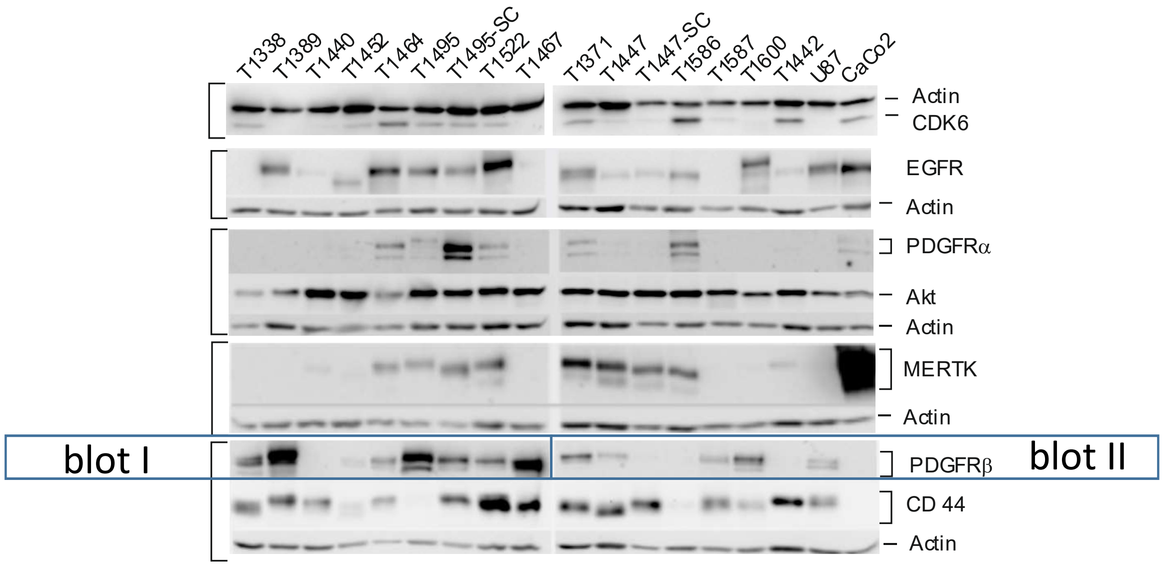

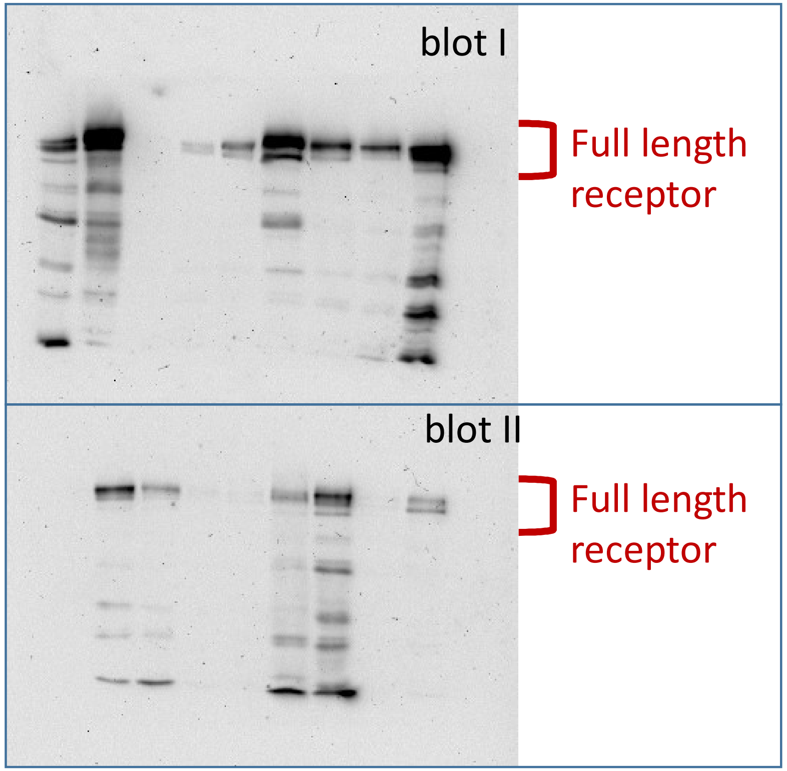

Fig. 2C → CD44

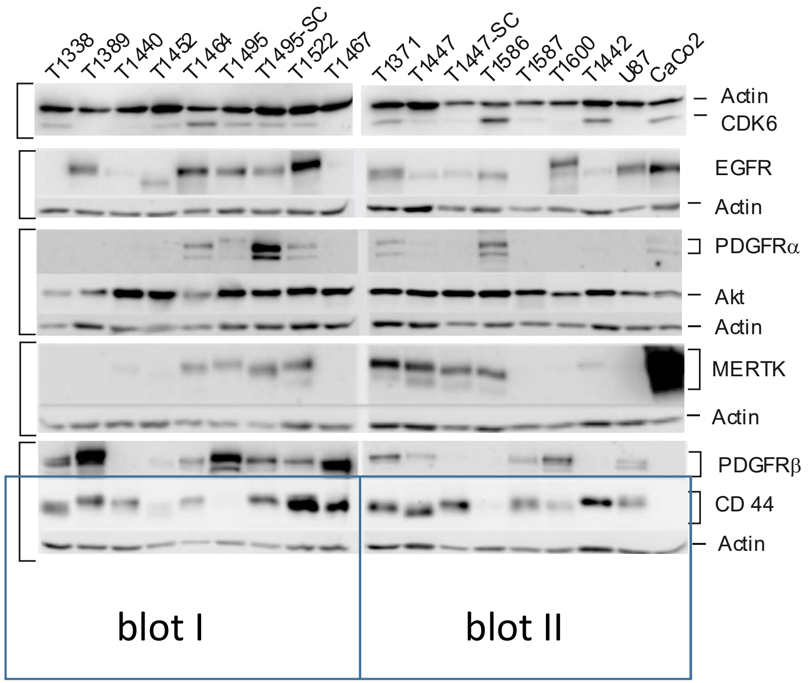
T1338
T1389
T1440
T1452
T1464
T1495
T1495-SC
T1522
T1467
T1371
T1447
T1447-SC
T1586
T1587
T1600
T1442
U87
CaCo2
Actin
CDK6
EGFR
Actin
PDGFRα
Akt
Actin
MERTK
Actin
PDGFRβ
CD 44
Actin
blot I
blot II

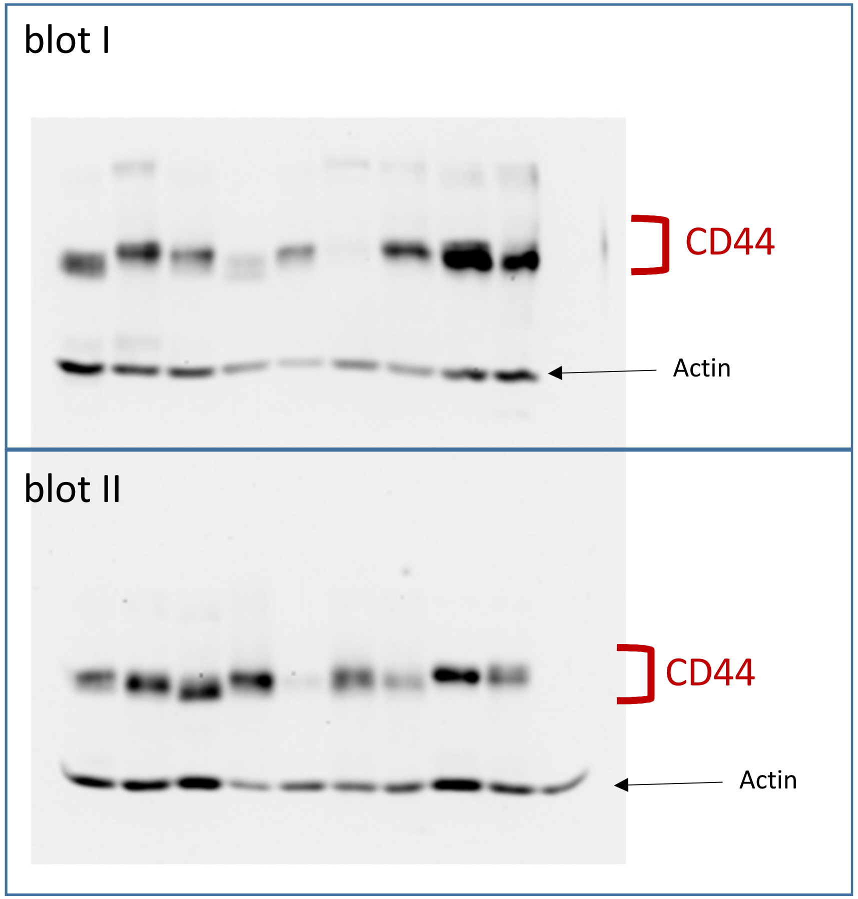
blot I
CD44
Actin
blot II
CD44
Actin

Fig. 7A → Neurofilaments (NF)

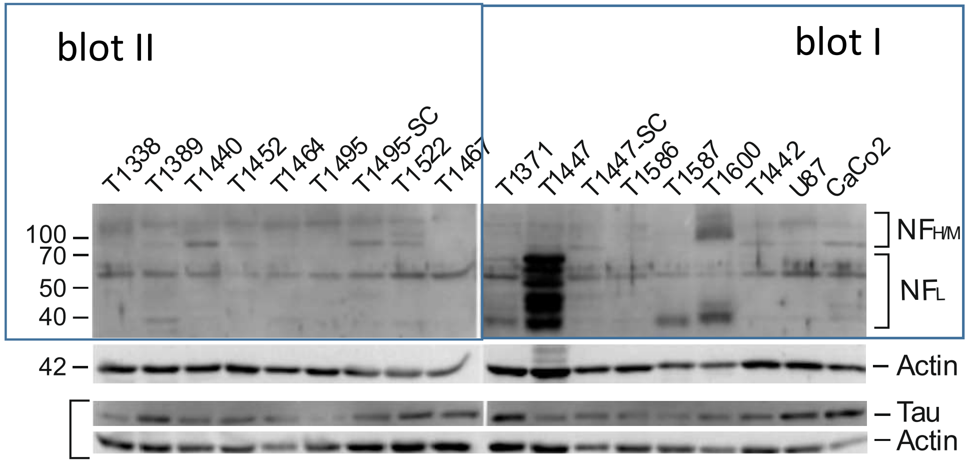

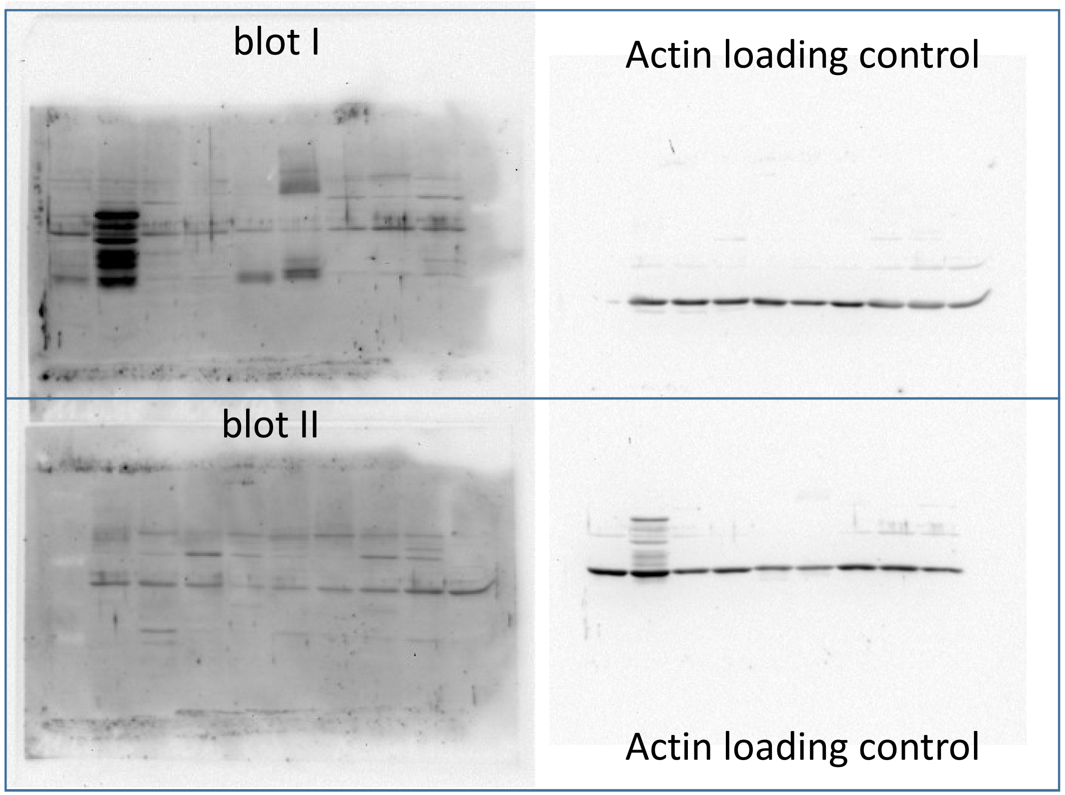

Fig. 7A → Tau (78.9 kDa)

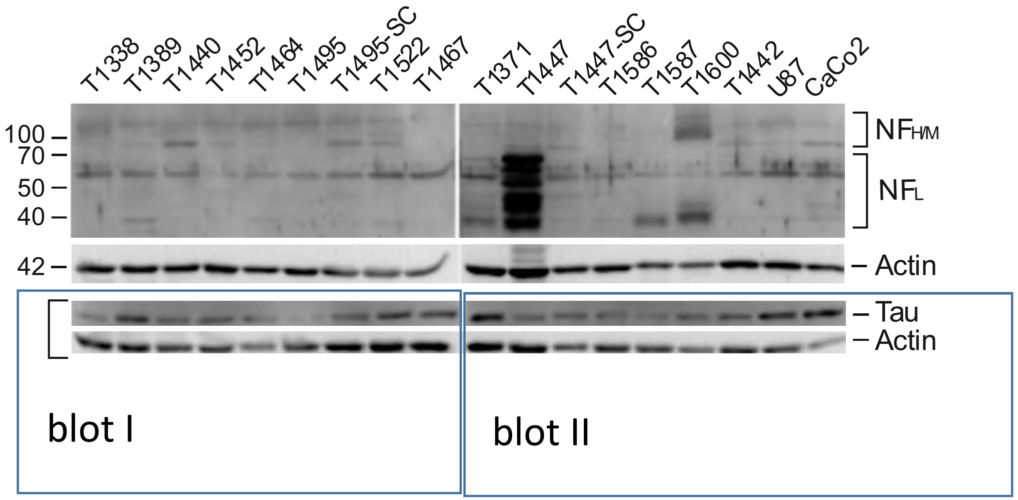

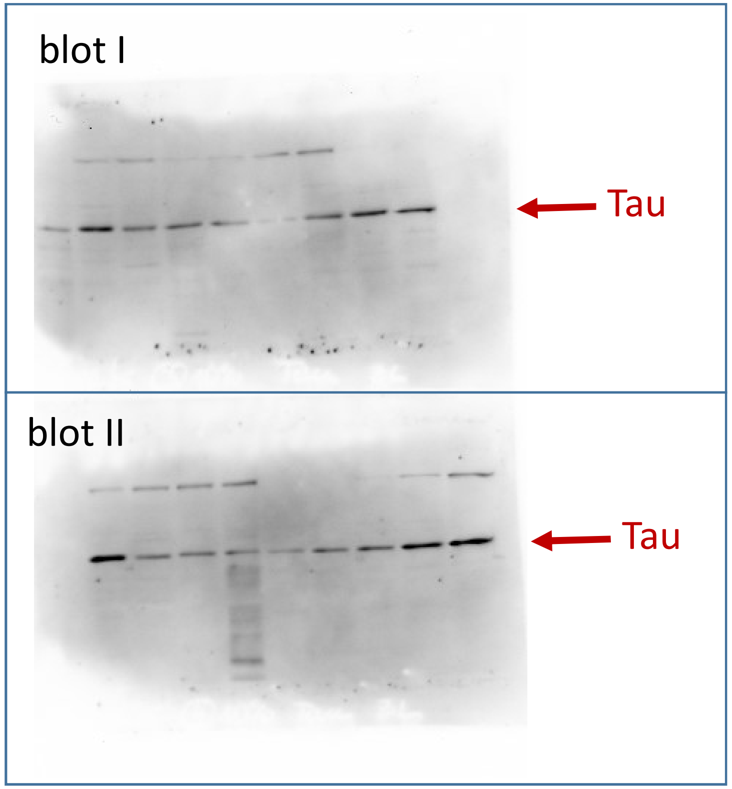

Fig. 7B → DARPP32

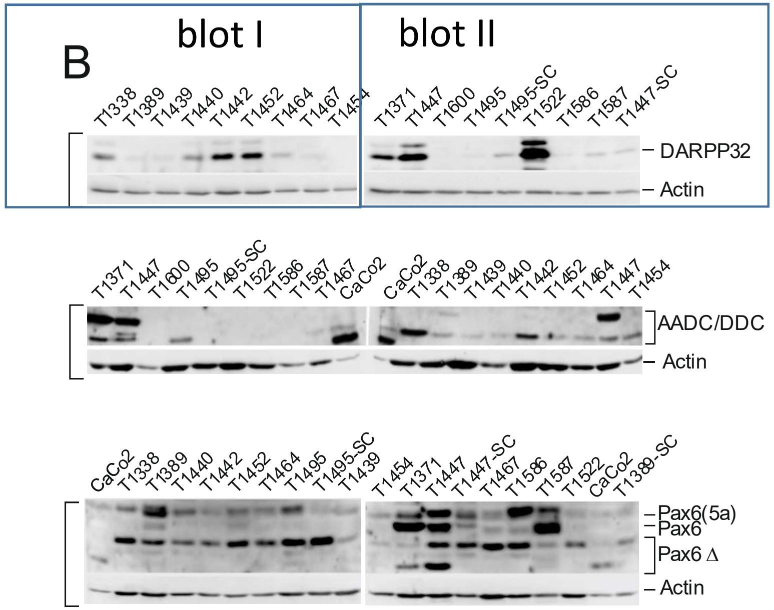

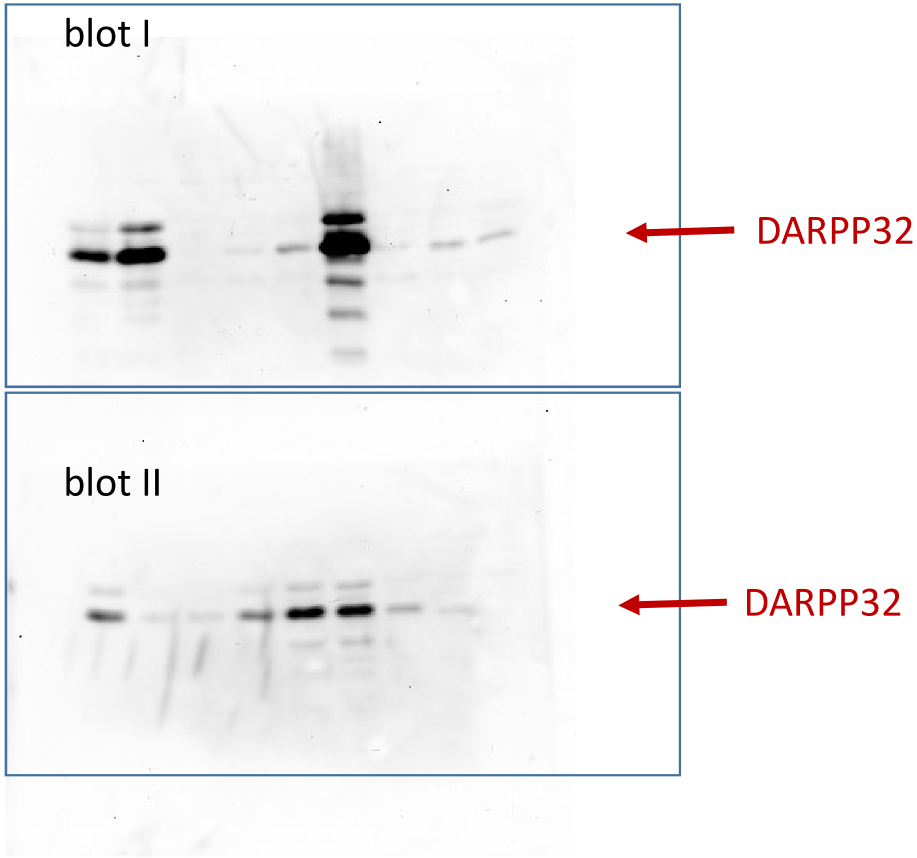

Fig. 7B → AADC/DDC

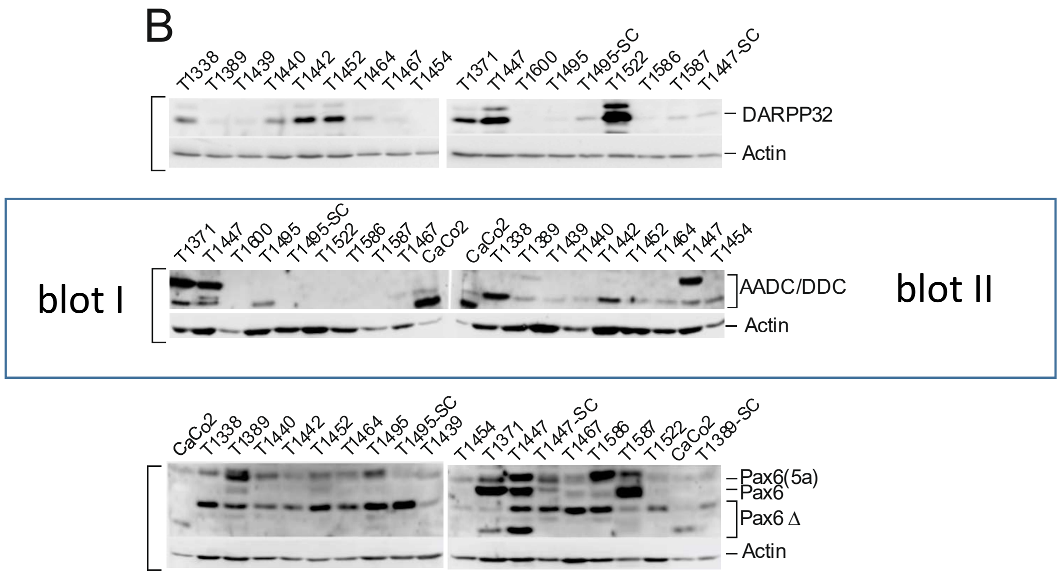

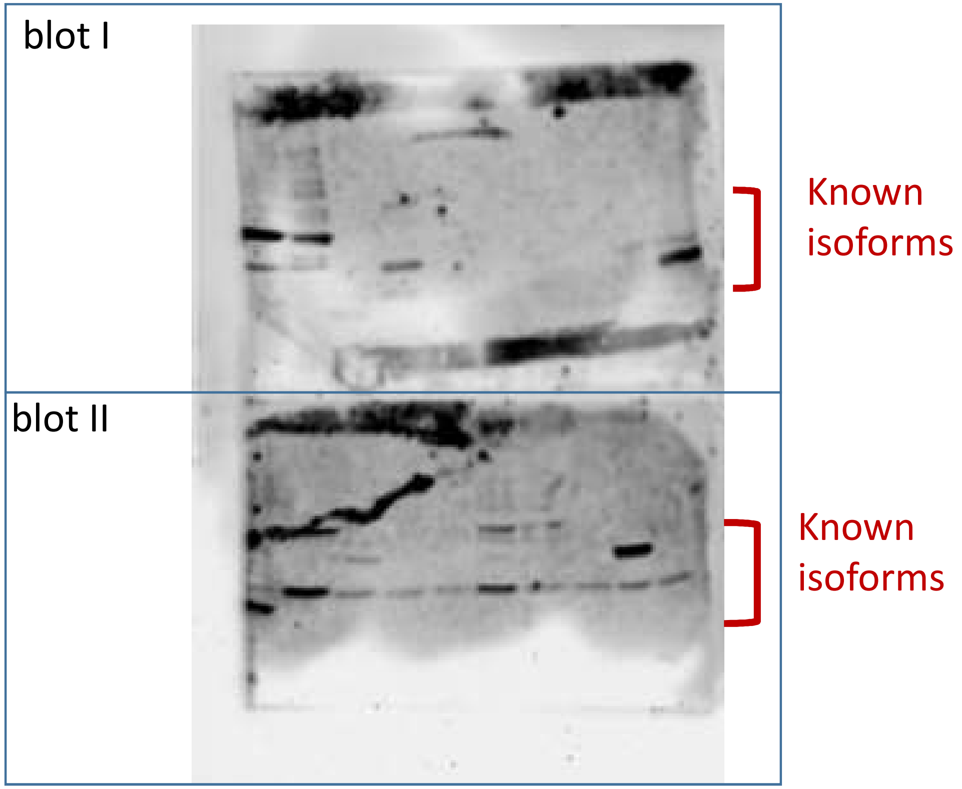

Fig. 7B → Pax6

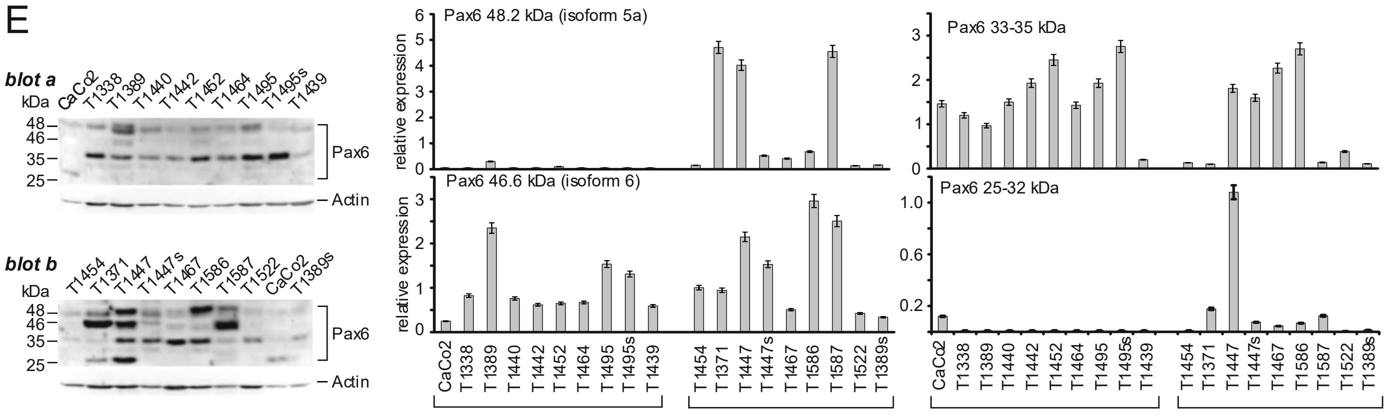
E
blot a
kDa
CaCo2 T1338 T1389 T1440 T1442 T1452 T1464 T1495 T1495s T1439
48
46
35
25
Pax6
Actin
blot b
T1454 T1371 T1447 T1447s T1467 T1586 T1587 T1522 CaCo2 T1389s
Pax6 48.2 kDa (isoform 5a)
Pax6 33-35 kDa
Pax6 46.6 kDa (isoform 6)
Pax6 25-32 kDa
relative expression

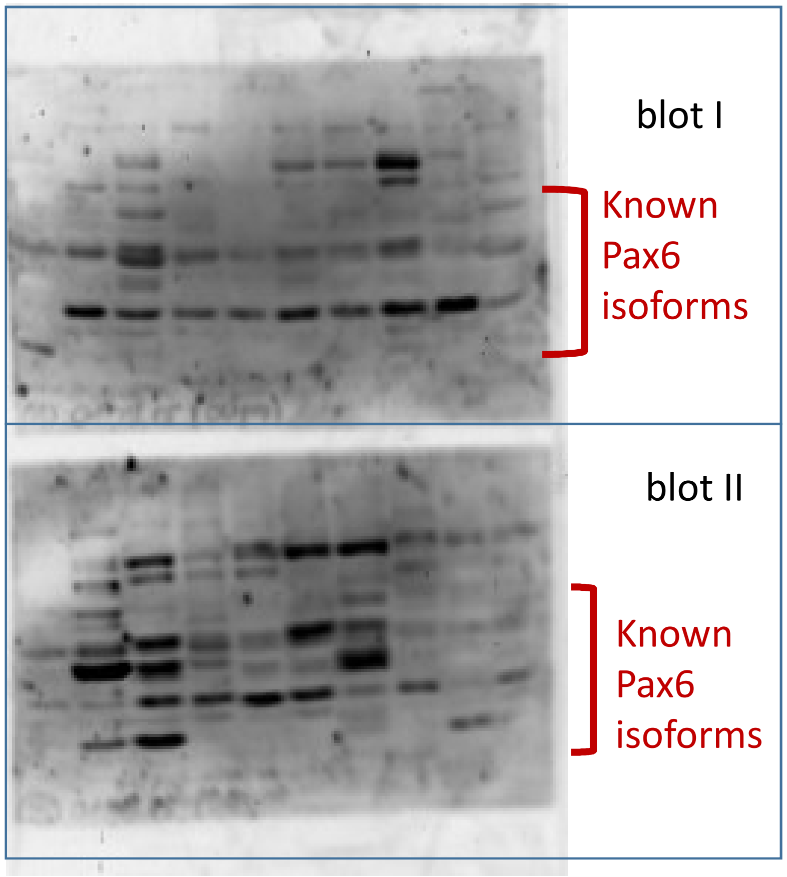
blot I
Known Pax6 isoforms
blot II
Known Pax6 isoforms

Fig. 7C → ABCG2

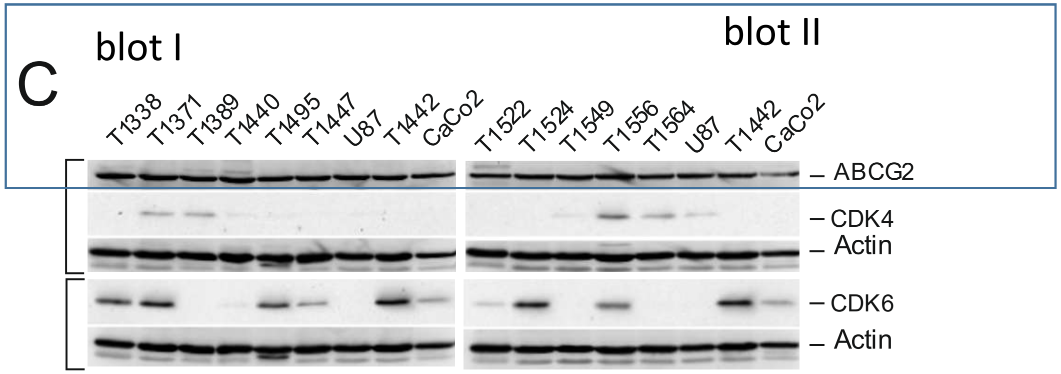
C
blot I
blot II
T1338
T1371
T1389
T1440
T1495
T1447
U87
T1442
CaCo2
T1522
T1524
T1549
T1556
T1564
U87
T1442
CaCo2
ABCG2
CDK4
Actin
CDK6
Actin

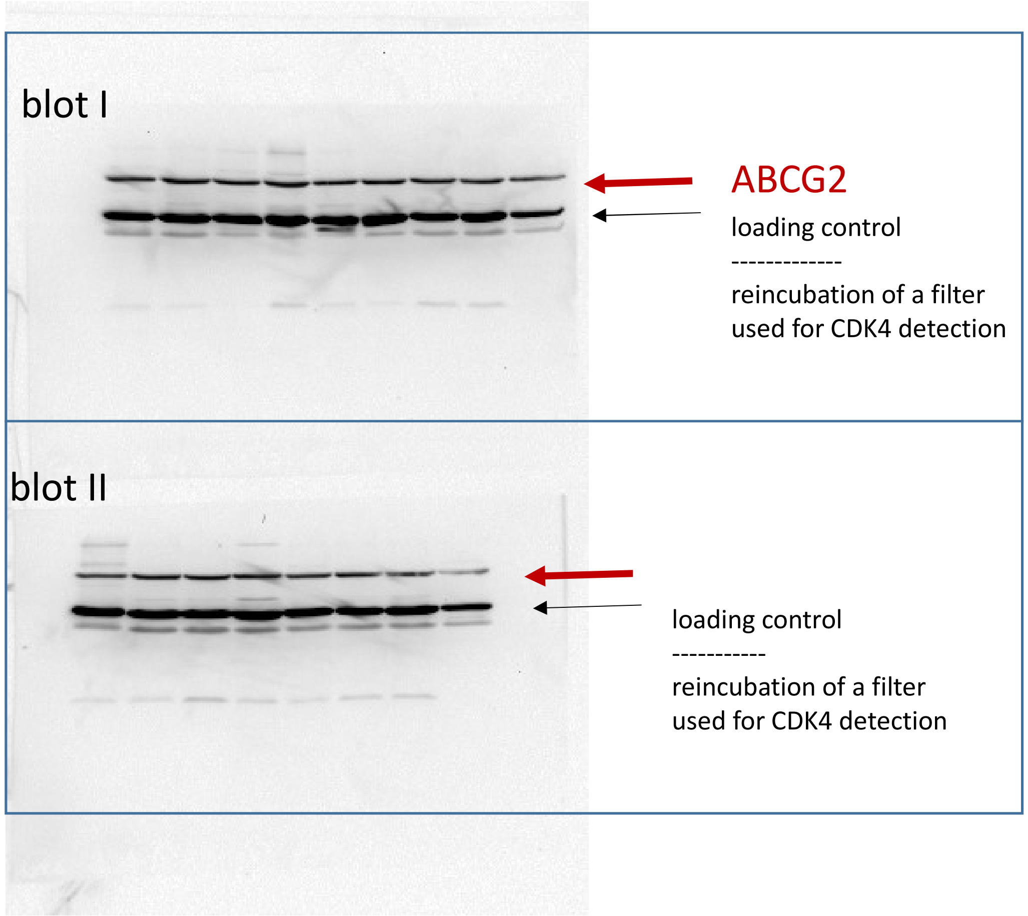
blot I
ABCG2
loading control
----
reincubation of a filter
used for CDK4 detection
blot II
loading control
----
reincubation of a filter
used for CDK4 detection

Fig. 7C → CDK4

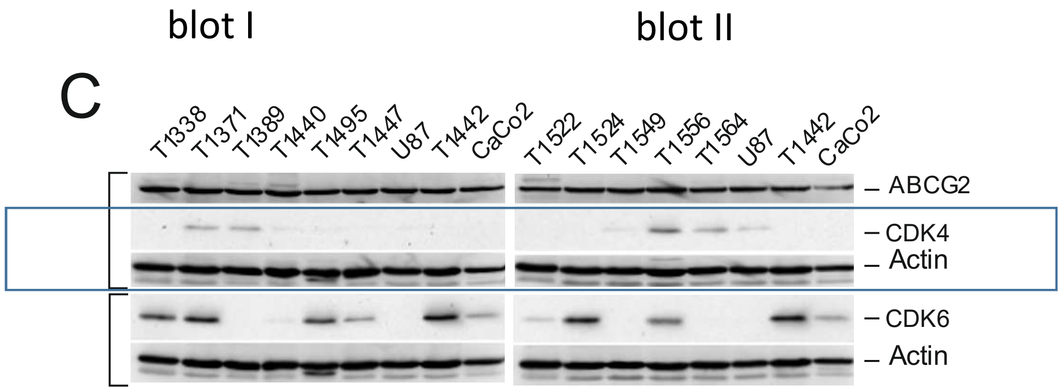
blot I
blot II
C
T1338
T1371
T1389
T1440
T1495
T1447
U87
T1442
CaCo2
T1522
T1524
T1549
T1556
T1564
U87
T1442
CaCo2
ABCG2
CDK4
Actin
CDK6
Actin

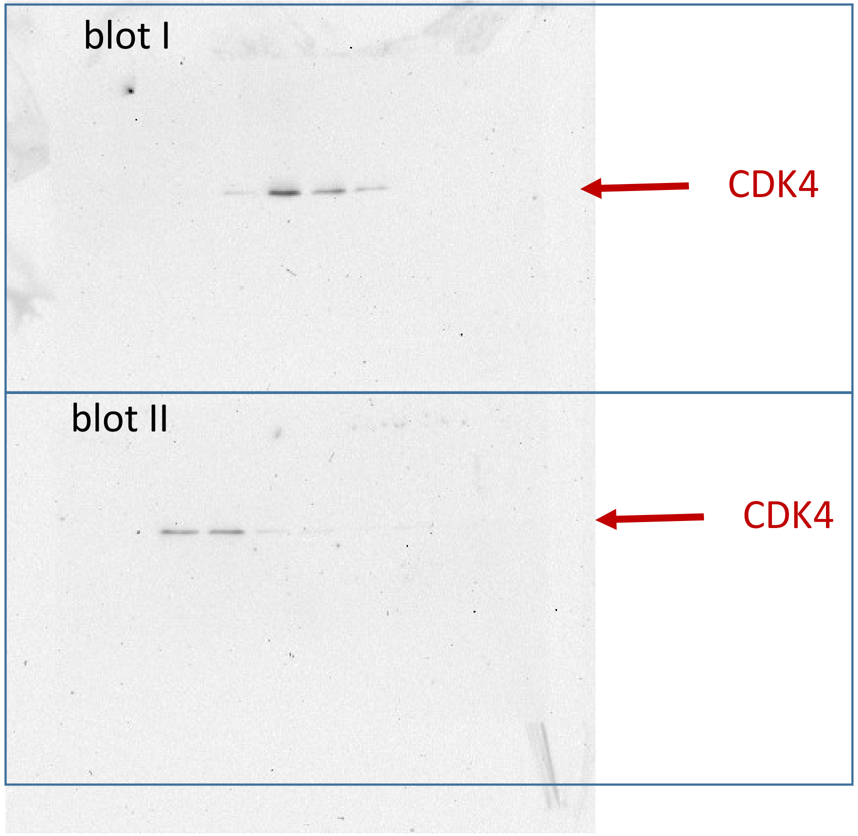
blot I
CDK4
blot II
CDK4

Fig. 7C → CDK6

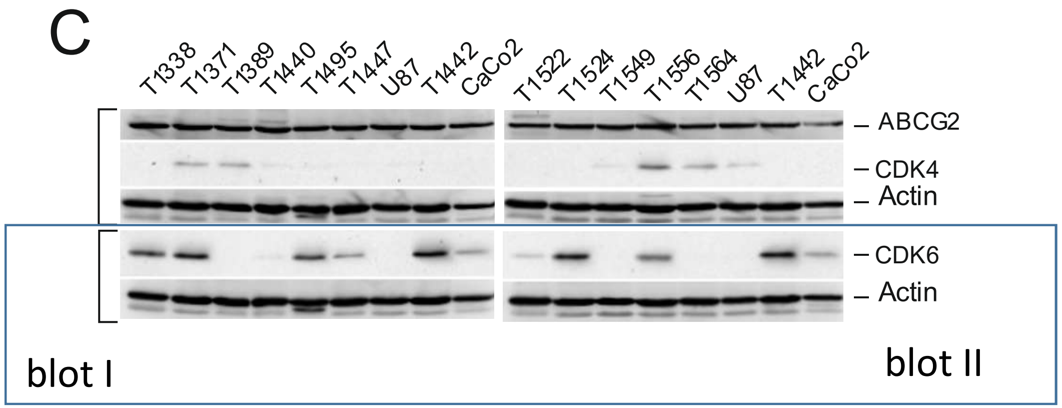
C
T1338
T1371
T1389
T1440
T1495
T1447
U87
T1442
CaCo2
T1522
T1524
T1549
T1556
T1564
U87
T1442
CaCo2
ABCG2
CDK4
Actin
CDK6
Actin
blot I
blot II

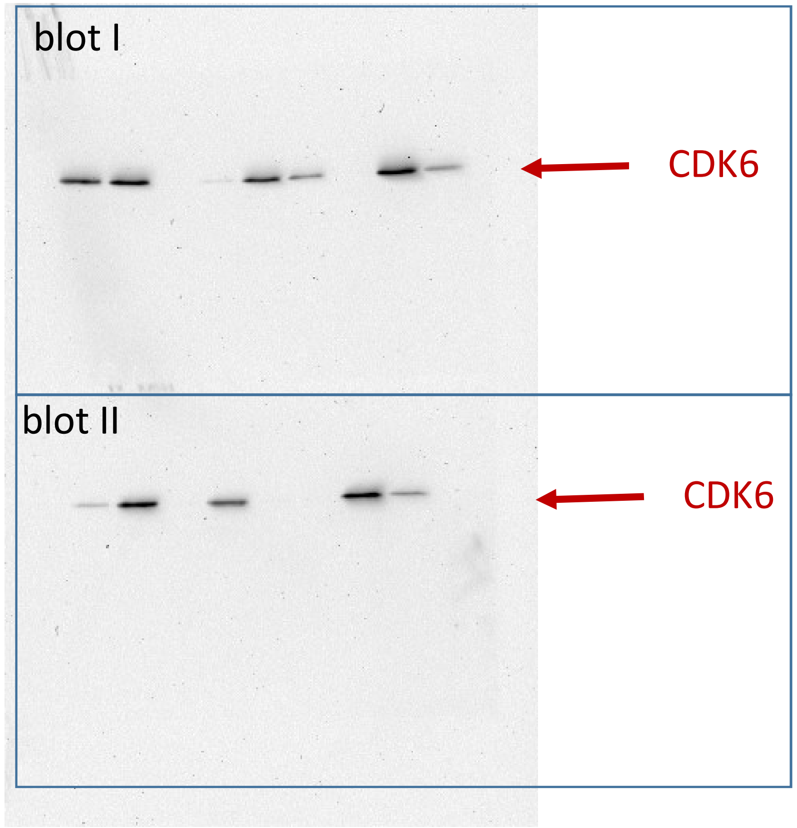
blot I
CDK6
blot II
CDK6

suppl. Figure 3

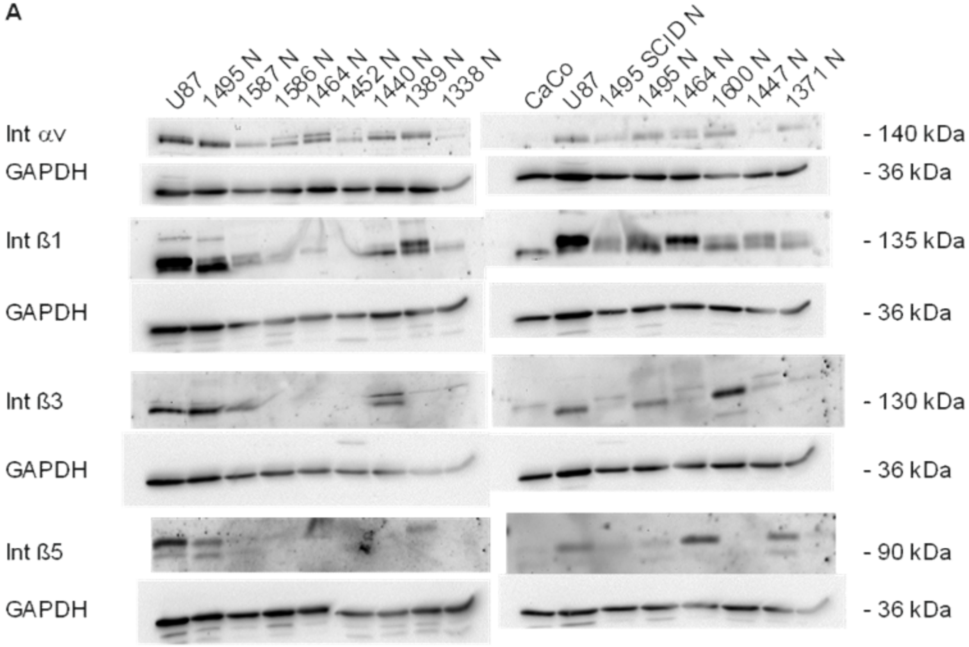

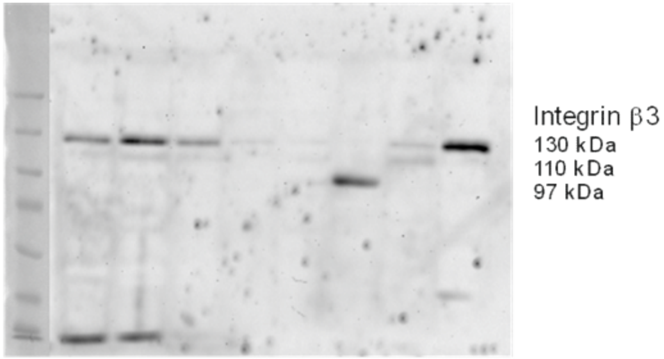

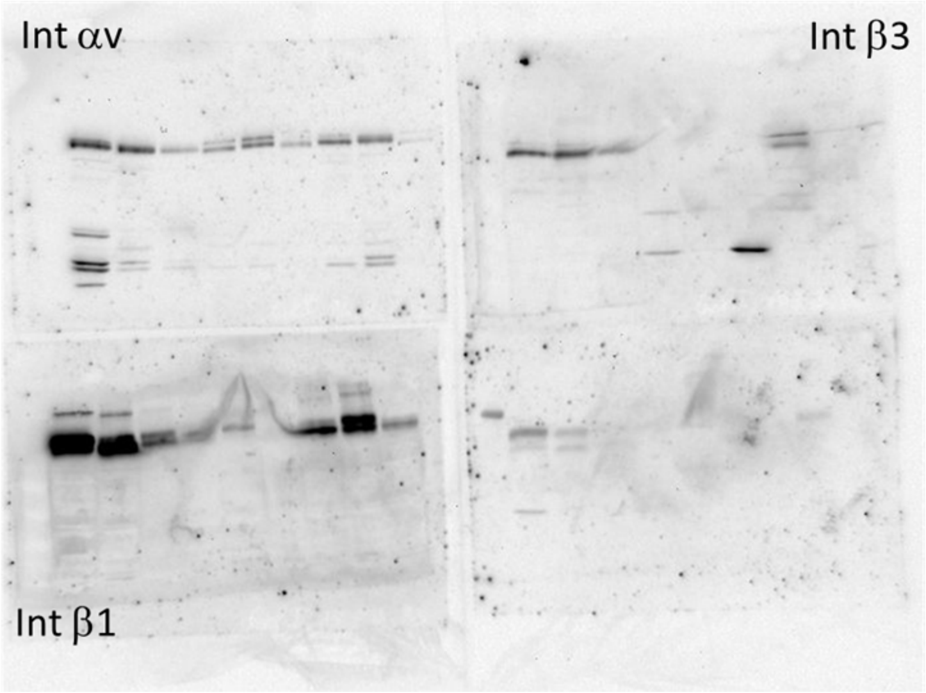

suppl. Fig. 5 → CD133

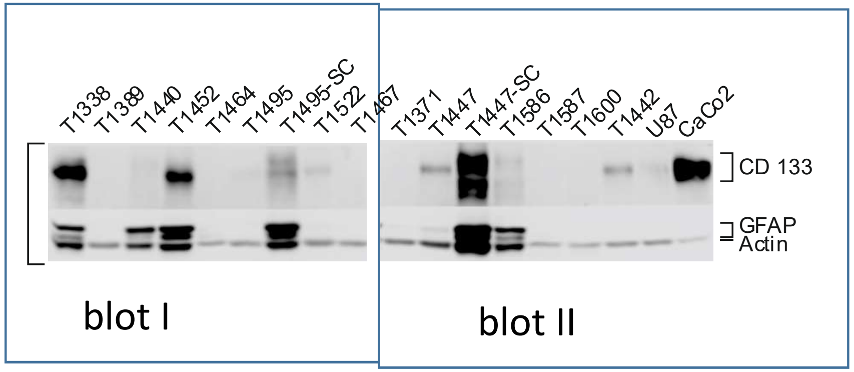

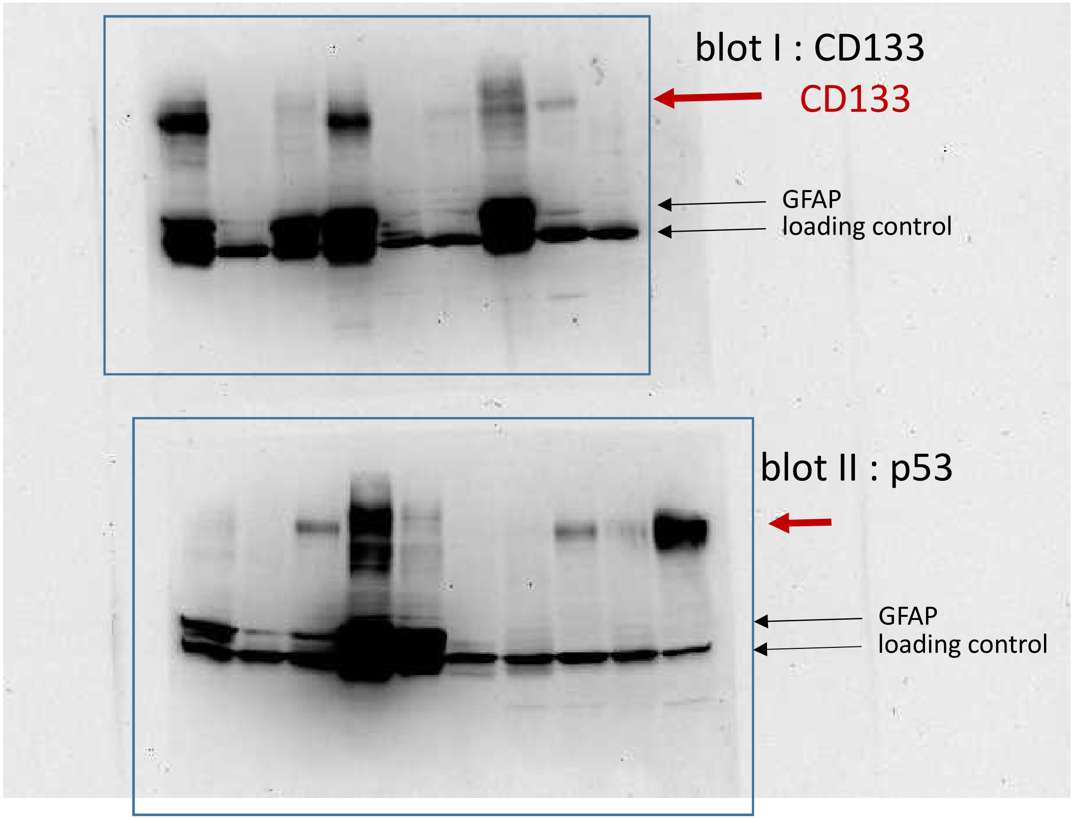

suppl. Fig. 5 → GFAP on CD133 blot

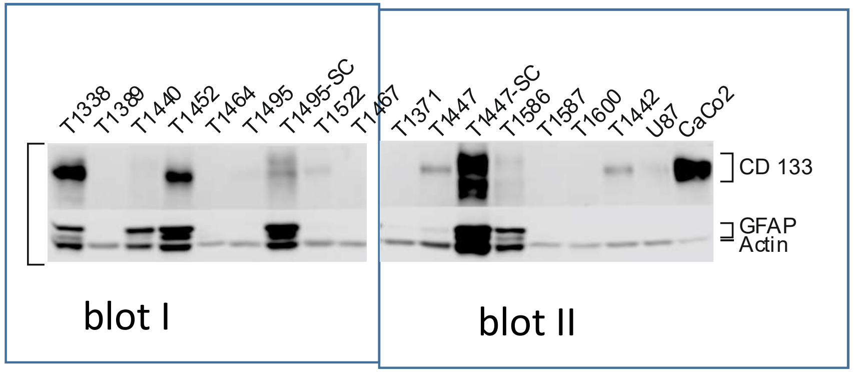

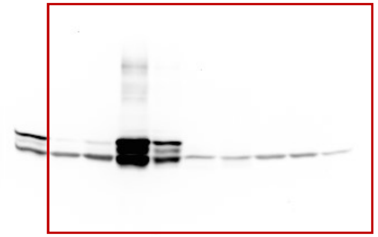

Shorter exposure GFAP (full length: 50 kDa)

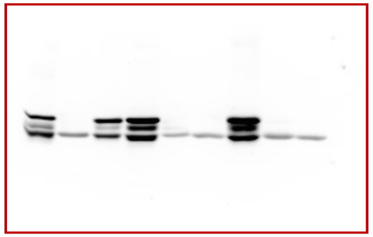

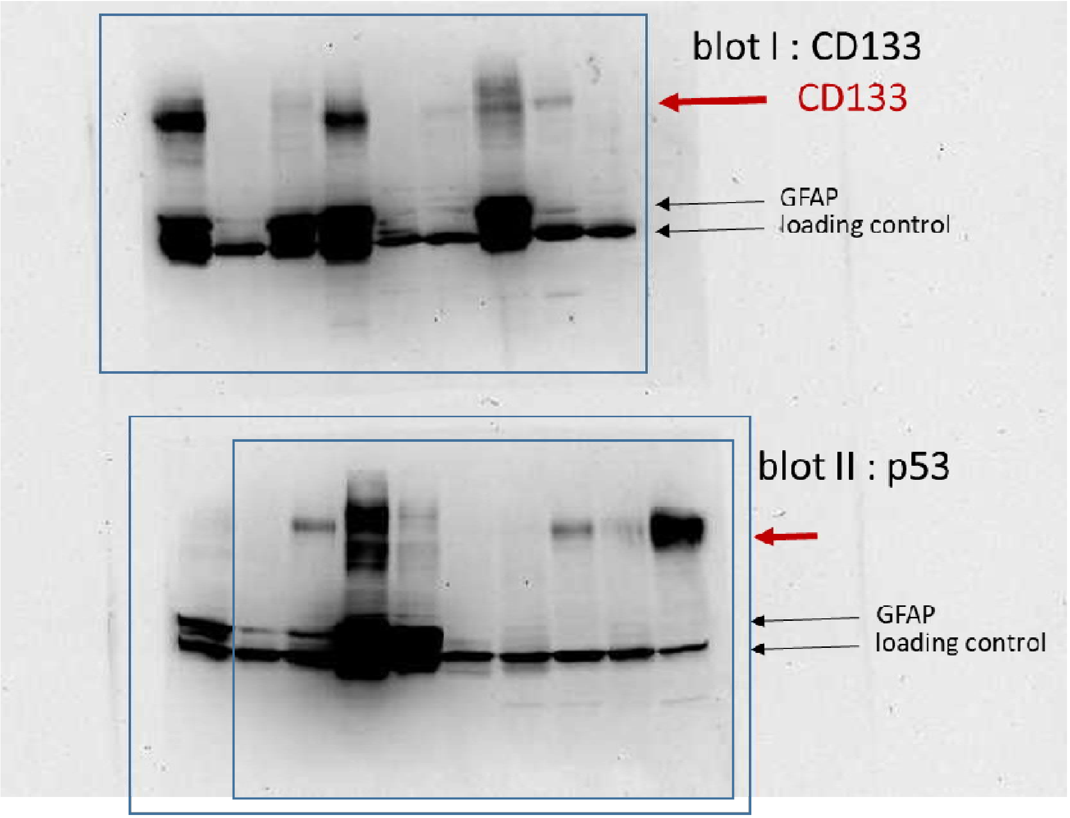

suppl. Fig. 5 → part IDH 1

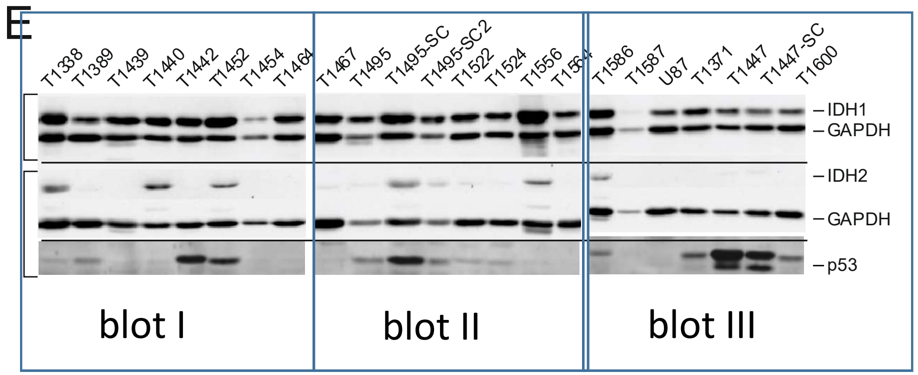

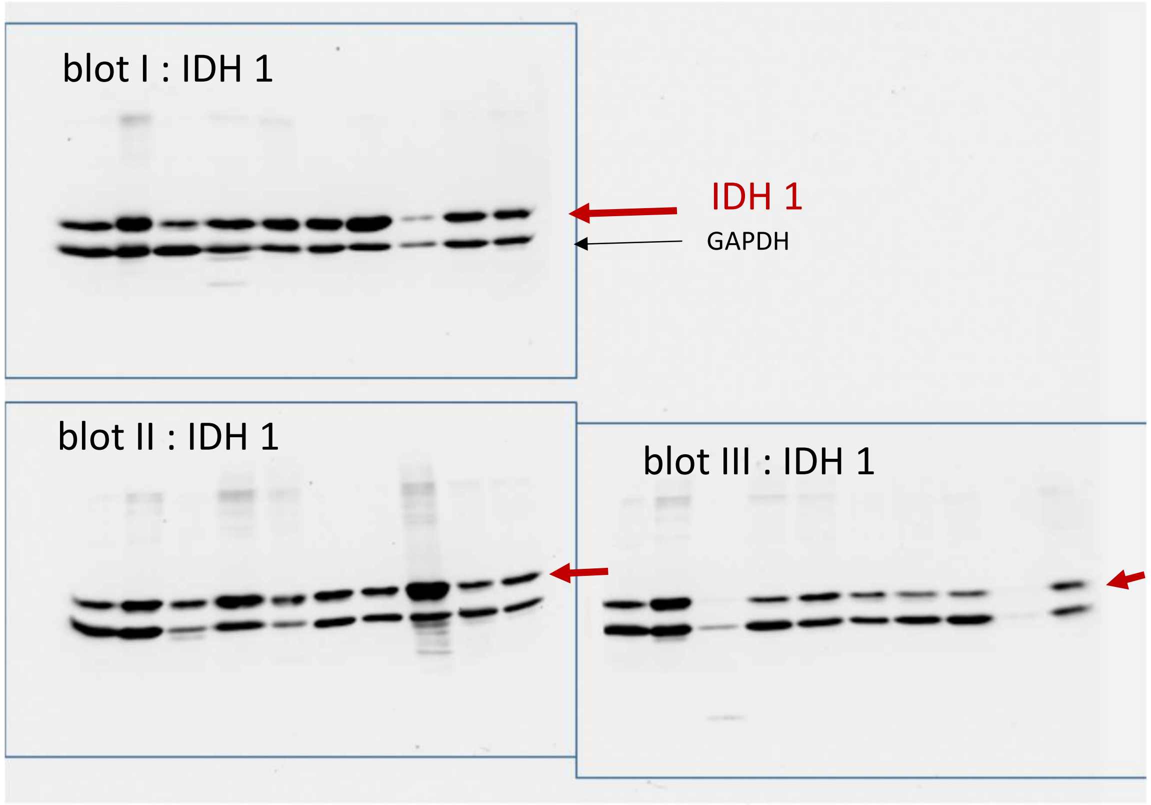

suppl. Fig. 5 → part IDH 2

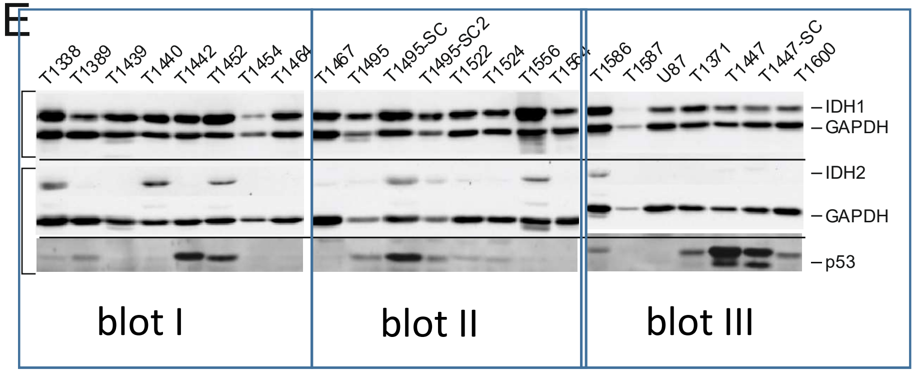

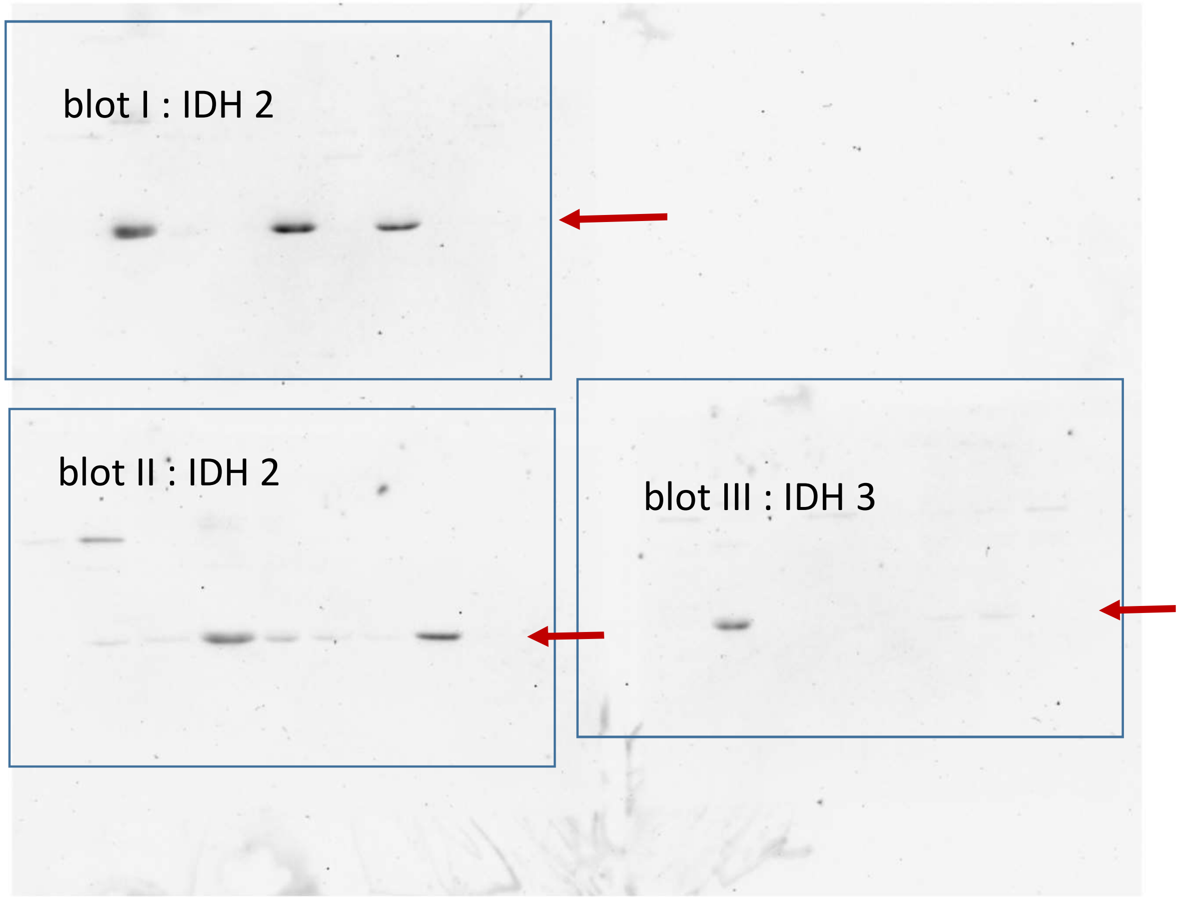

suppl. Fig. 5 → part p53

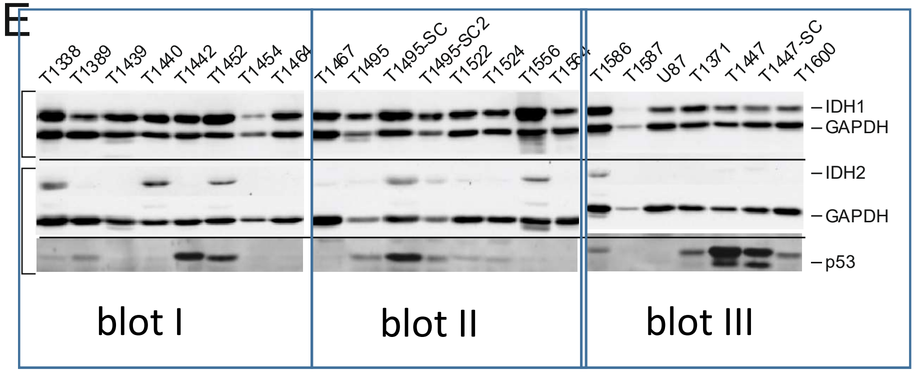

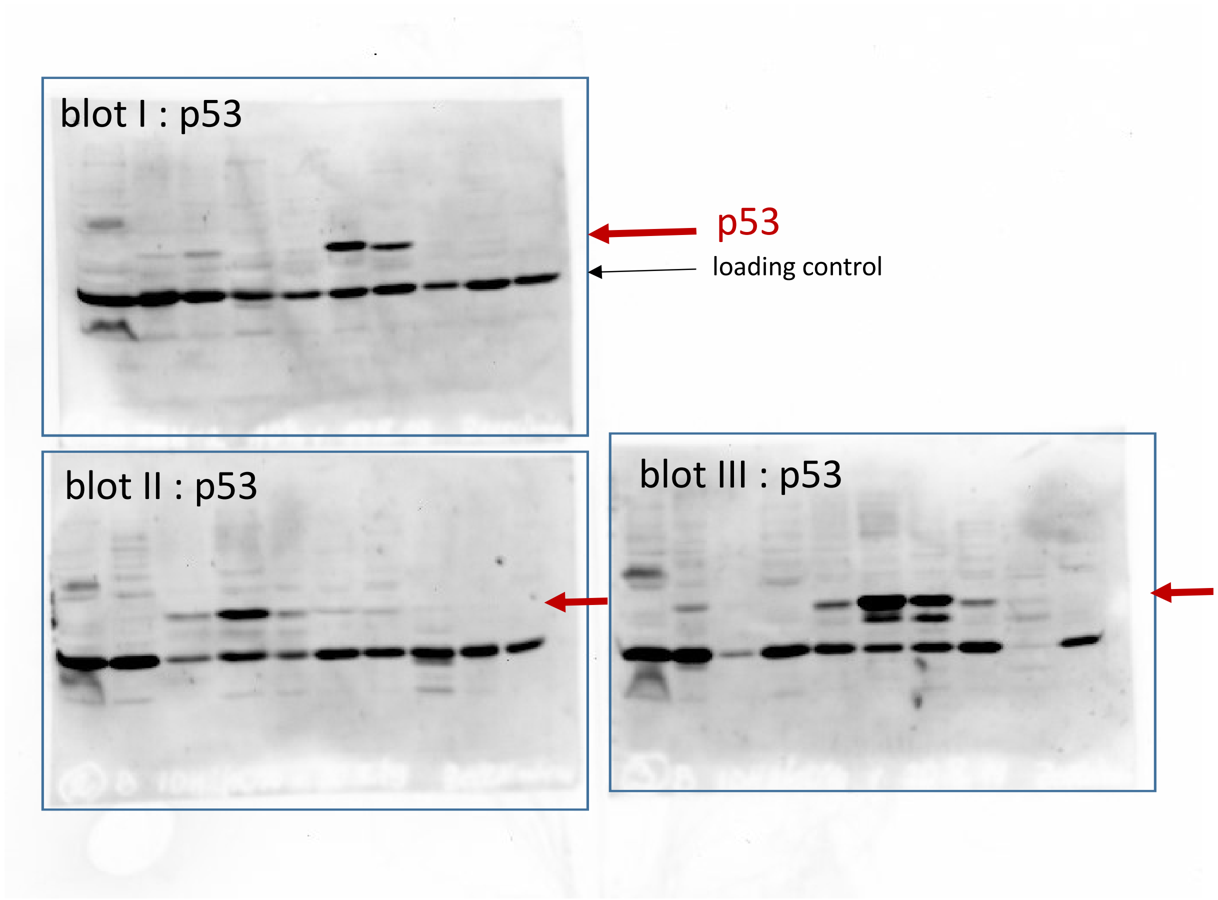

suppl. Fig. 9A

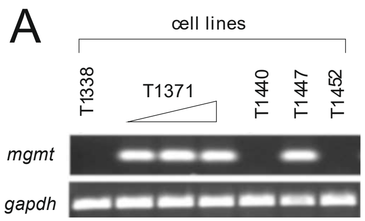

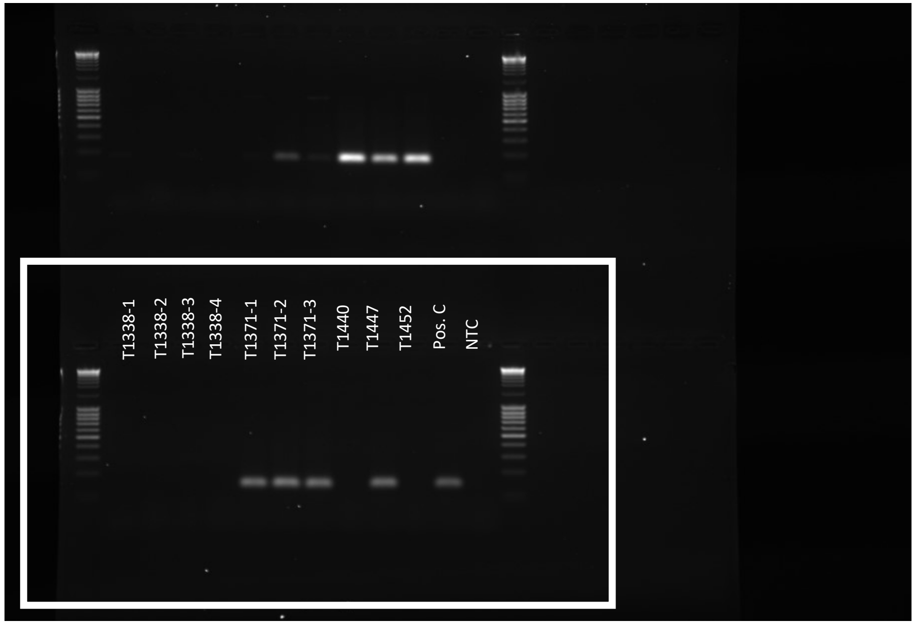

Fig. 9A: expression of *mgmt* mRNA (RT-PCR analysis)
- pos. C: positive control
- NTC: non-template control

suppl. Fig. 9B

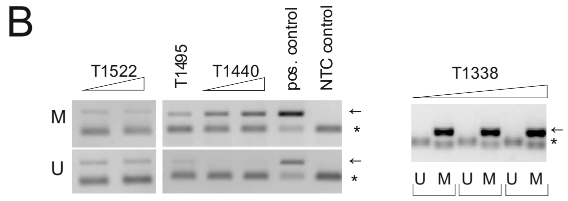

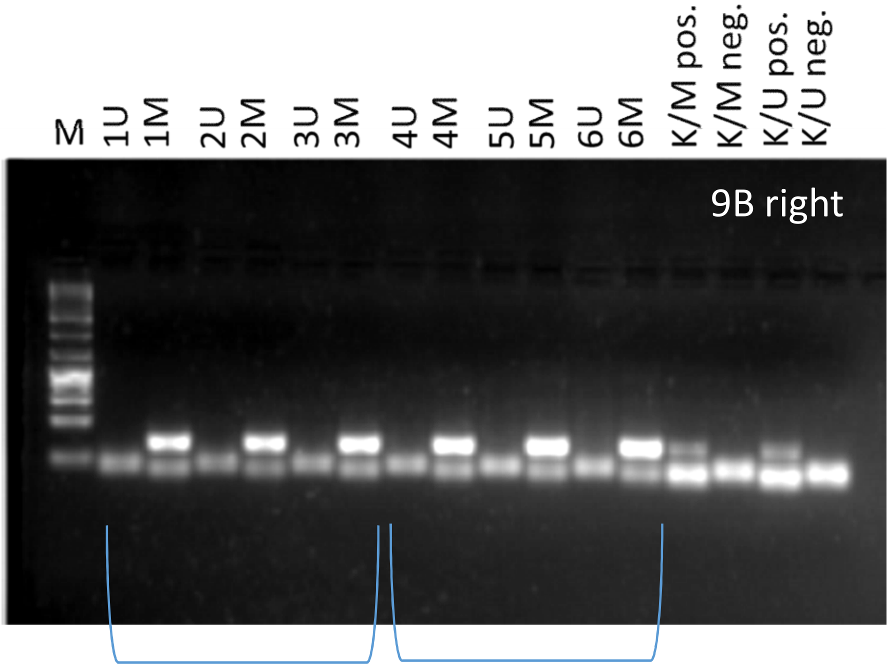

T1338 increasing passages

T1440 increasing passages

K/M pos: pos control M primer
K/M neg: non-template control M primer

K/U pos: pos control U primer
K/U neg: non-template control U primer

9B left

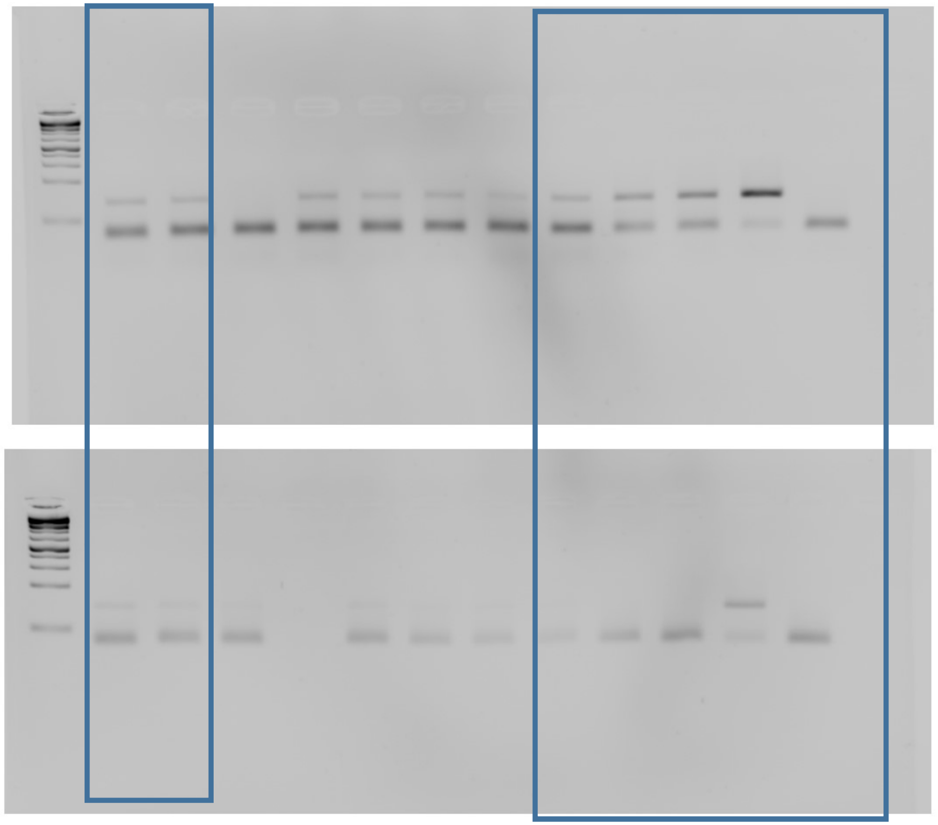

suppl. Fig. 9C → MSP data

C gel1

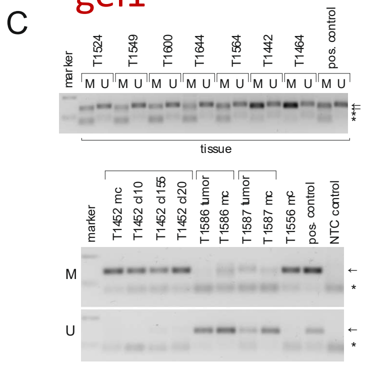

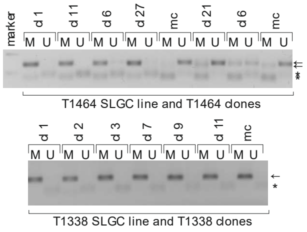

gel2

gel3

methylation status	cell lines
pure m (methylated)	T1338, T1440, T1556, U87
pure u (unmethylated)	T1371, CaCo2

gel1

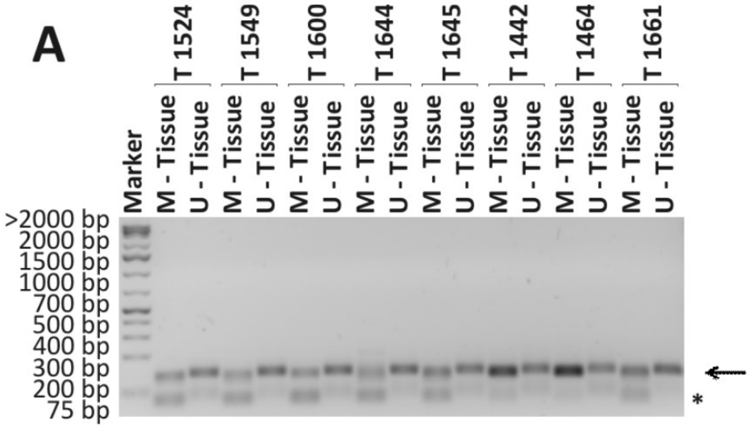

gel3

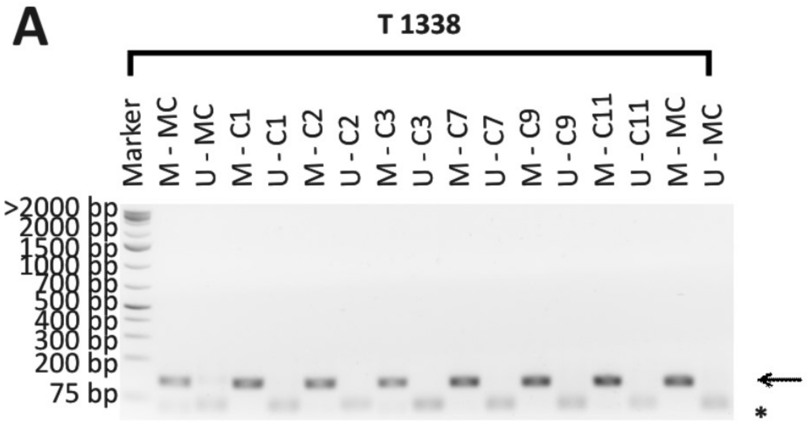

gel2

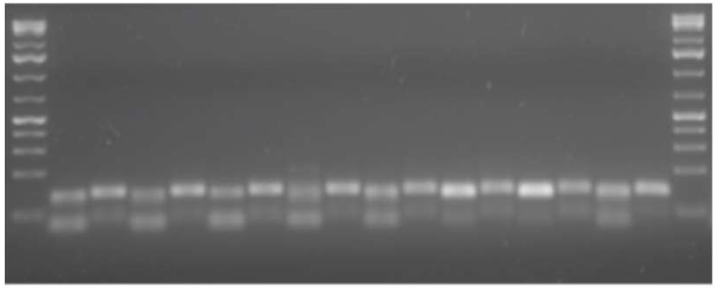

suppl. Fig. 9C → MSP data

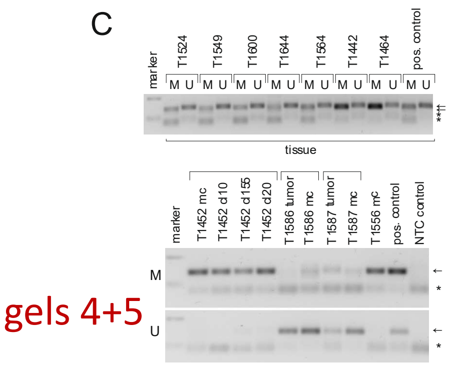

gels 4+5

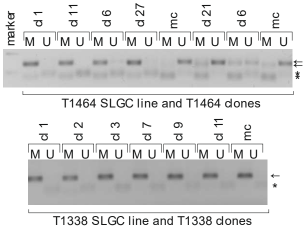

methylation status	cell lines
pure m (methylated)	T1338, T1440, T1556, U87
pure u (unmethylated)	T1371, CaCo2

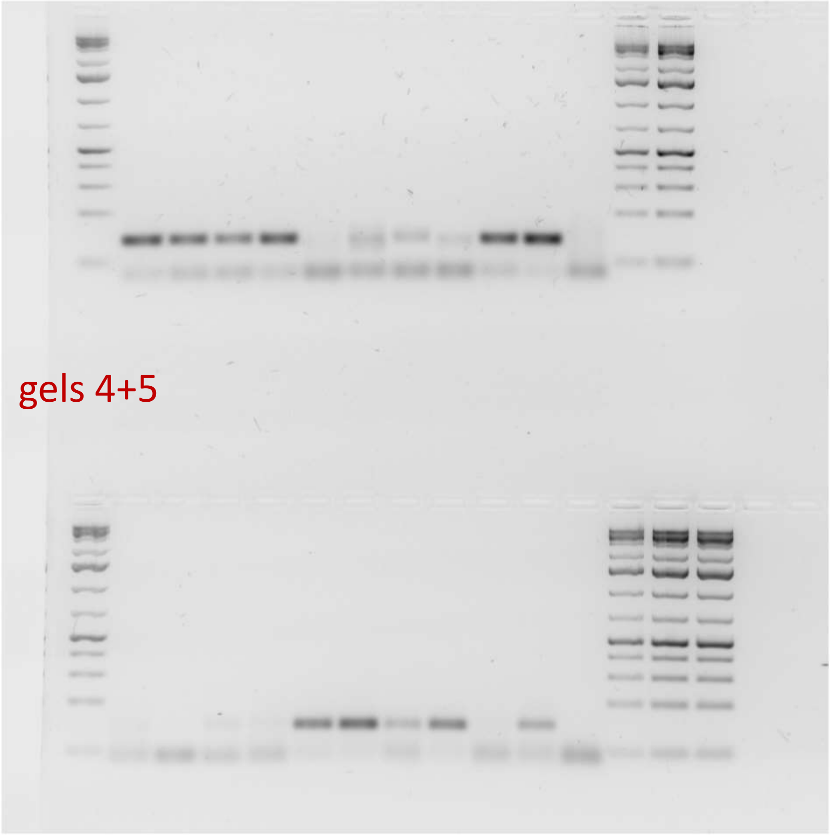

gels 4+5

suppl. Fig. 9E → MGMT

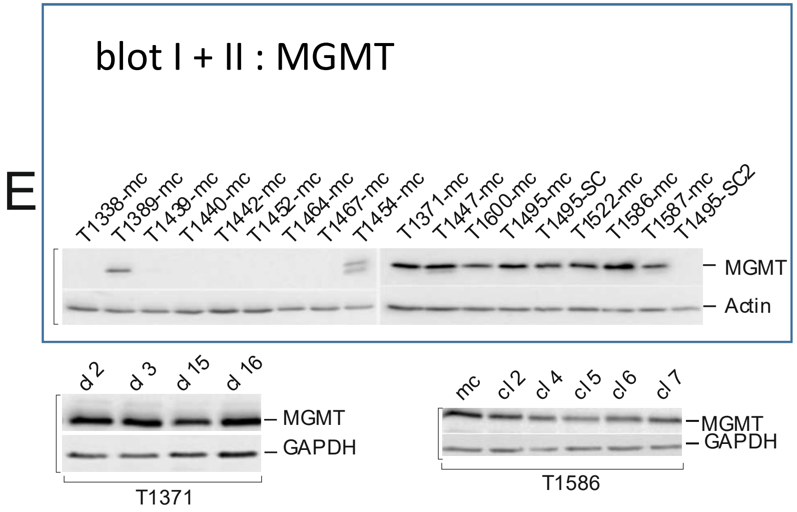

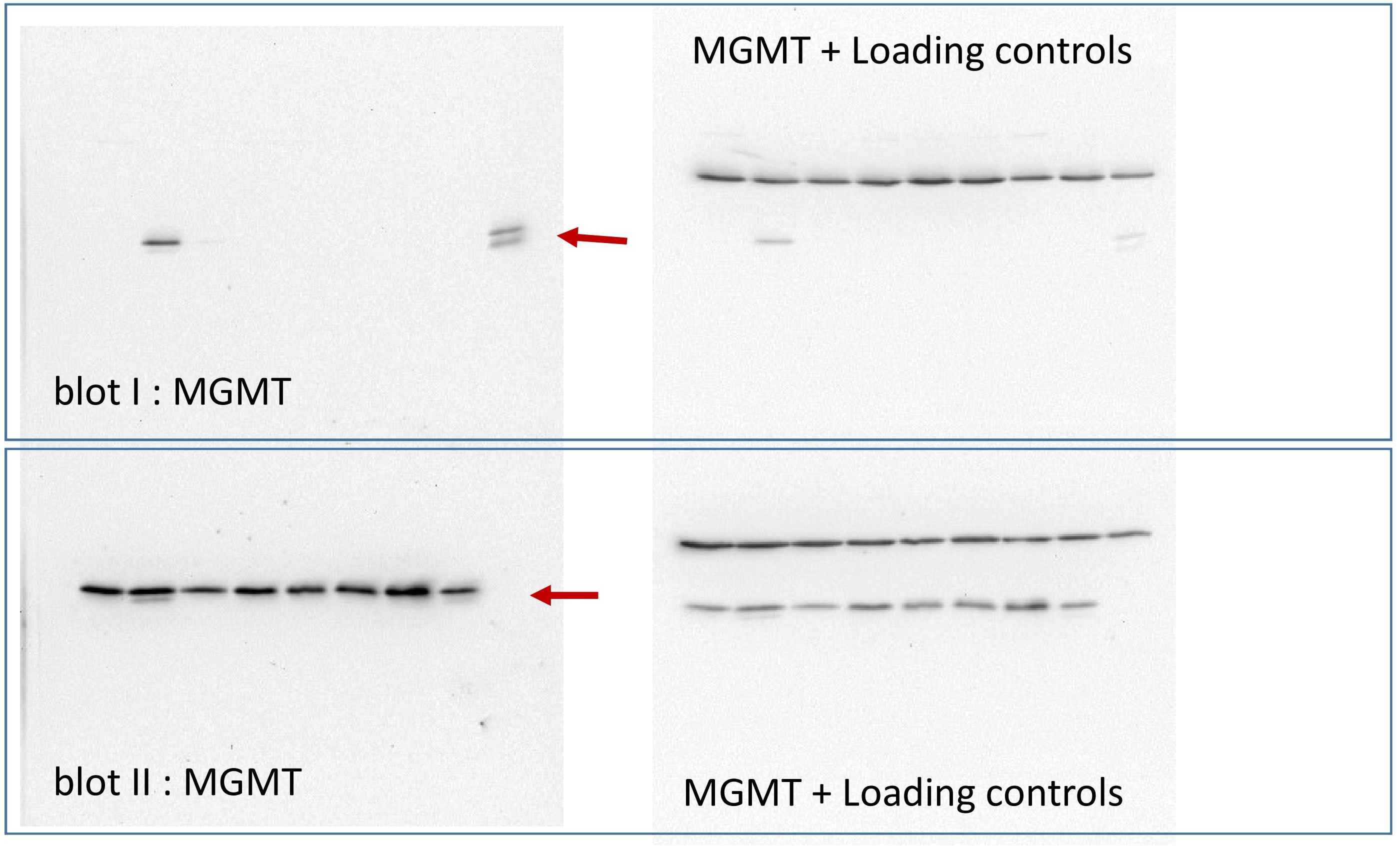

suppl. Fig. 9E → MGMT

E

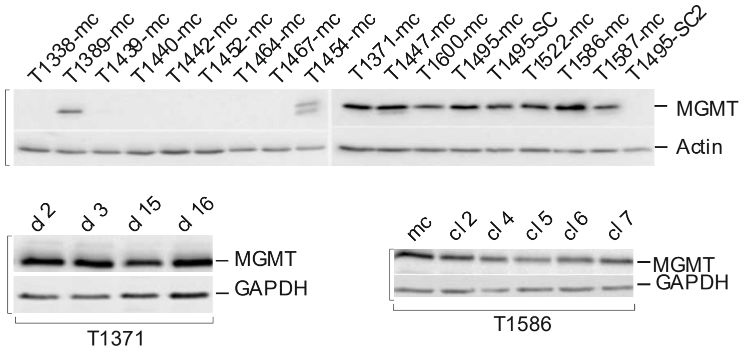

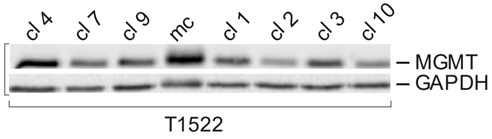

blot III: MGMT

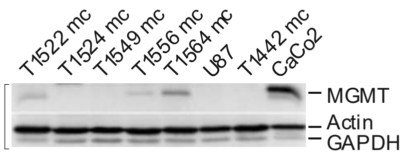

- antibody was generated against a BSA-MGMT fusion protein; therefore, the antibody detects BSA (*) traces derived from the N-medium in WCE;
- Detection of MGMT (20 kDa) was on a blot used for CDK4 (34 kDa) detection before → signal labelled with **

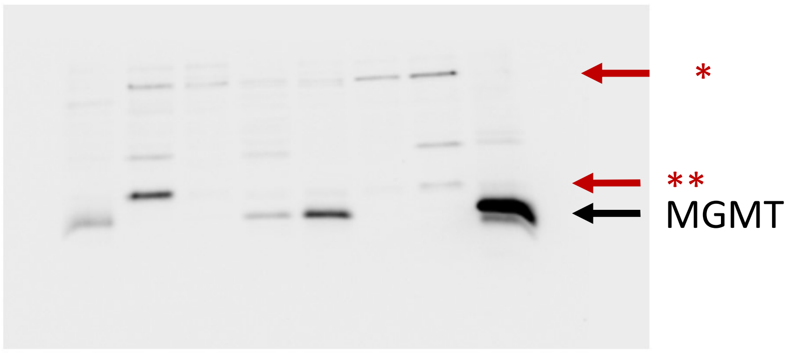

suppl. Fig. 9E

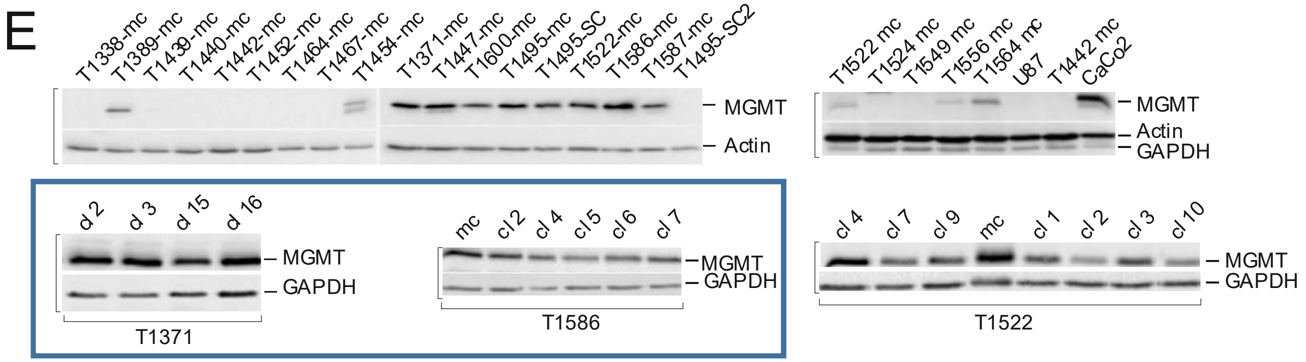

- antibody was generated against a BSA-MGMT fusion protein; therefore, the antibody detects BSA traces derived from the N-medium in WCE;

- Blots were incubated with an antibody aginst p21CIP1 before of the MGMT detection

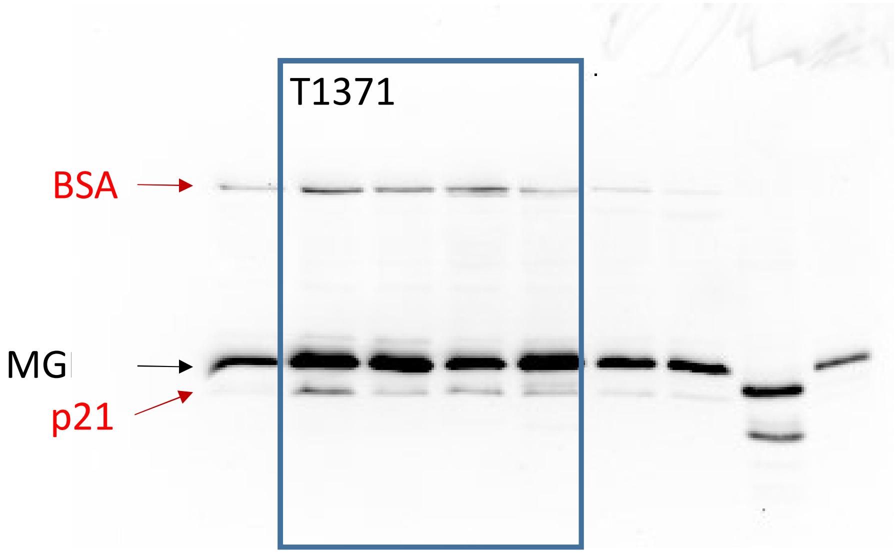

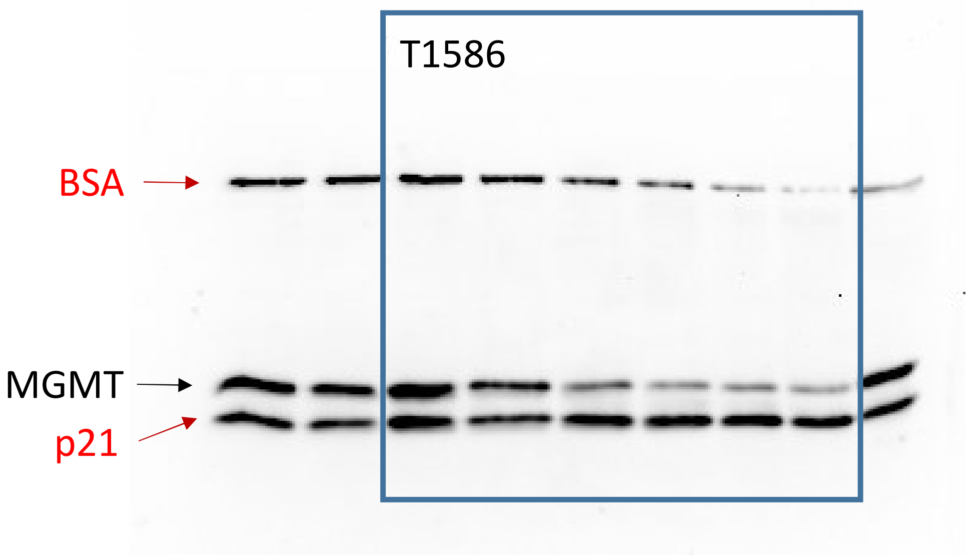

suppl. Fig. 9E

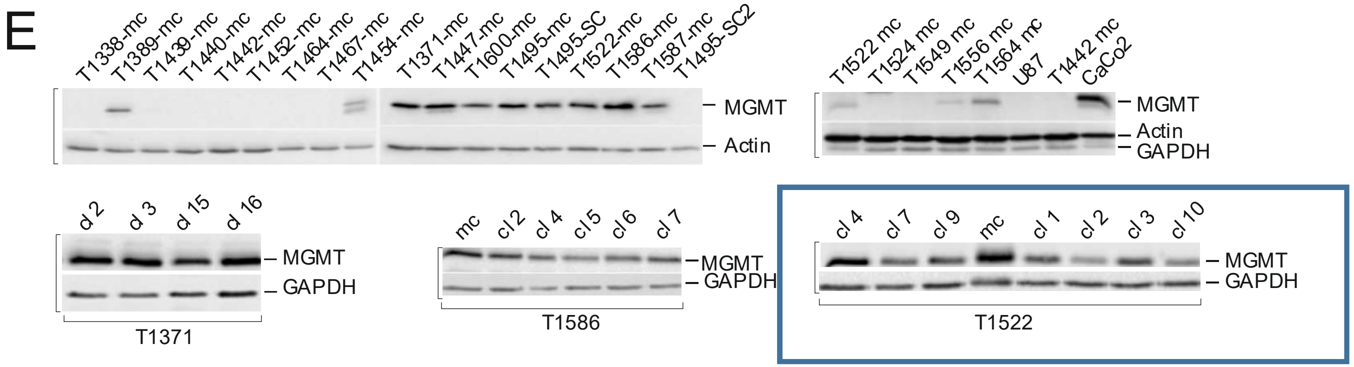

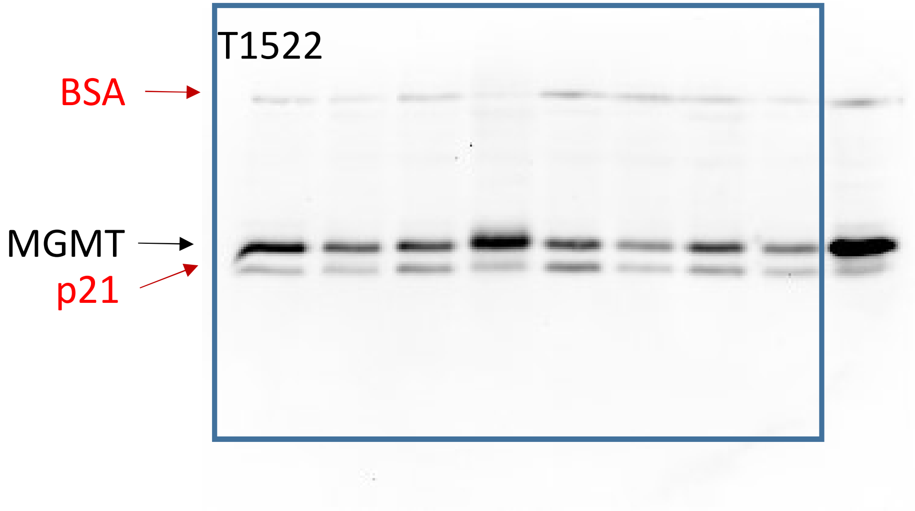

- antibody was generated against a BSA-MGMT fusion protein; therefore, the antibody detects BSA traces derived from the N-medium in WCE;
- Blots were incubated with an antibody aginst p21CIP1 before the MGMT detection
