# Supplementary material for: Molecular signature of stem-like glioma cells (SLGCs) from human glioblastoma and gliosarcoma
Source: PLoS One. 2024 Feb 2;19(2):e0291368. doi: 10.1371/journal.pone.0291368 (PMC10836714; doi:10.1371/journal.pone.0291368)

Supplementary data

Zechel et al.

Mouse model: processing of brains and stains :

- In order to reveal and characterize orthotopic tumors, hematoxylin and eosin (HE) staining was performed according to standard protocols.
- For this, mouse brains were cut in two halves at the puncture site, each half spanning both hemispheres. This was possible since the site of inoculation was visible as a small scar-like structure on the right hemisphere.
- Starting from the two cutting surfaces, we sliced the mouse brains with a microtome. Up to 20 slices (4.5 µm) were taken from each side. Every fourth slice underwent HE staining; mounting was done with Entellan.
- Microscopy was executed with the BZ8000 and the BZ9000 software. Microphotographs were analyzed for the presence of tumors.
- In cases in which tumors were observed, we determined the cross-section dimension from the photos. Due to the irregular shape of most tumors and their invasiveness, it appeared inappropriate to provide exact measures. - In this context, it has to be noted, that tumors may undergo some shrinkage during the fixation and/or staining procedures.
- Moreover, tumors may appear as structures with sharp boundaries in HE staining, though they are invasively growing. Therefore, immunohistochemistry (IHC) was performed to test for invasiveness. IHC was carried out with a rabbit antibody against Sox2 and a mouse antibody specific for the human protein stem121.
- IHC was also applied to reveal SLGCs in the background of more differentiated tumor cells.
- In the beginning we used three distinct concentrations of SLGC cells (50,000, 100,000, and 200,000). Since (i) we did not see any correlation between cell numbers and tumor size and (ii) only small tumors were generated with several SLGC lines, we decided to continue with 200,000 cells.
- Please note: only data for tumors with known p53 status are shown in the figures and are listed in the tables.

Examples for immunohistochemistry: Orthotopic tumors; staining with HE or an antibody against Sox2

T1464 - cl 18 – Sox2+ clusters and bulk cells and necrotic tumor center | T1495 – invasive region | T1495 – necrotic region

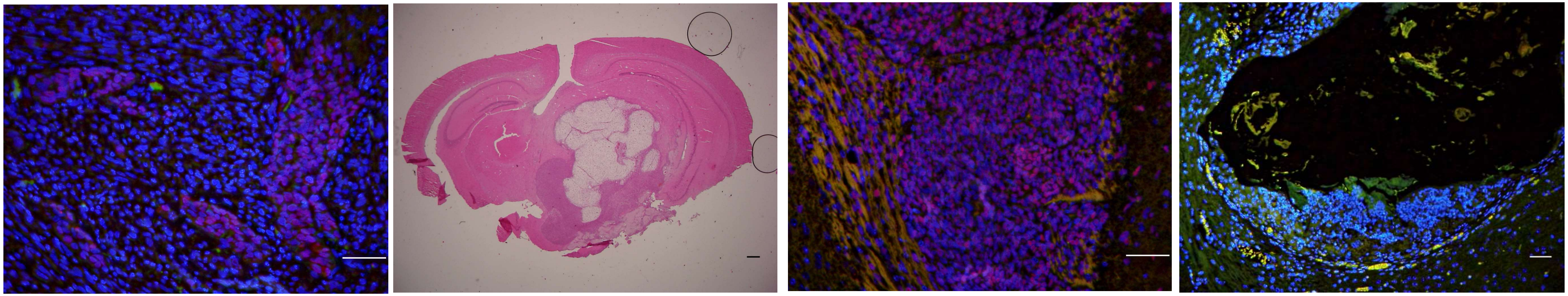

T1464 – Sox2+ clusters and bulk cells | T1440 – clone – sharp border | T1440 – clone – sharp border

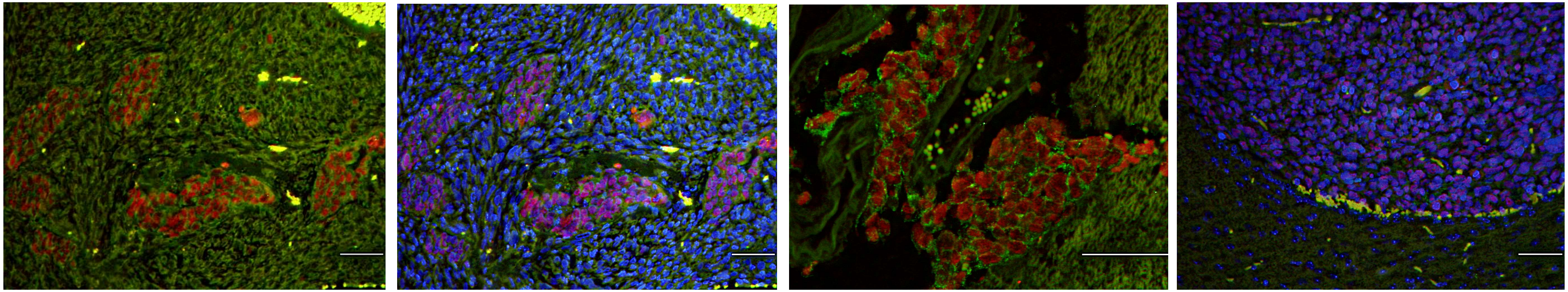

bar: 50 µm; z-stacks – Note: DAPI channel was omitted from some photos

Examples for immunohistochemistry: Orthotopic tumors; staining with Sox2 antibody – green: human specific marker

T1447

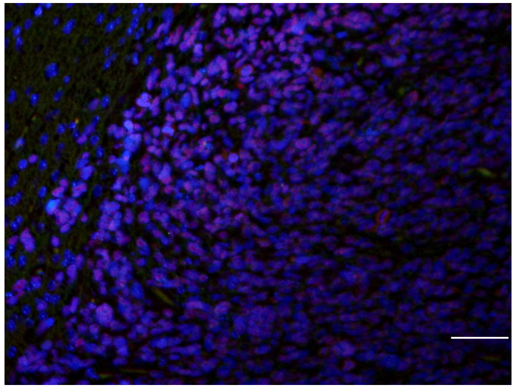

T1447

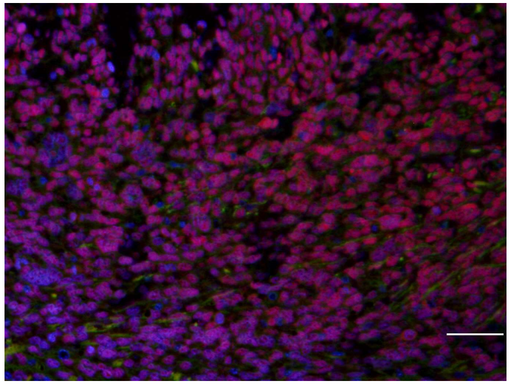

T1338 – cl7

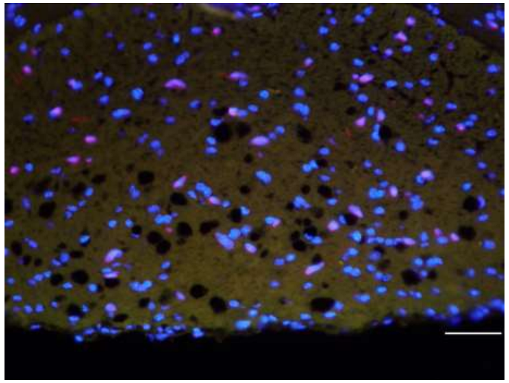

T1452 cl10 – sharp border

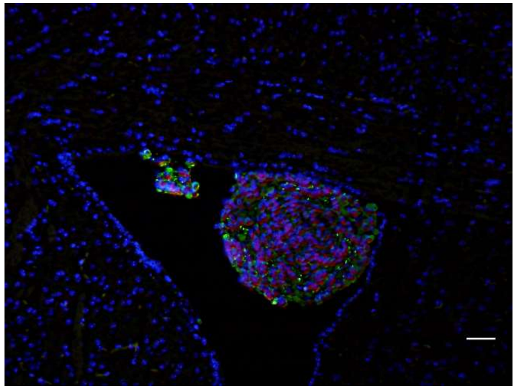

T1447 cl4 – invasive region

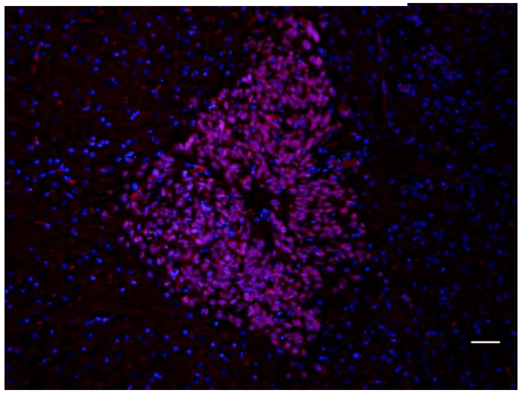

T1447 cl4 – invasive region

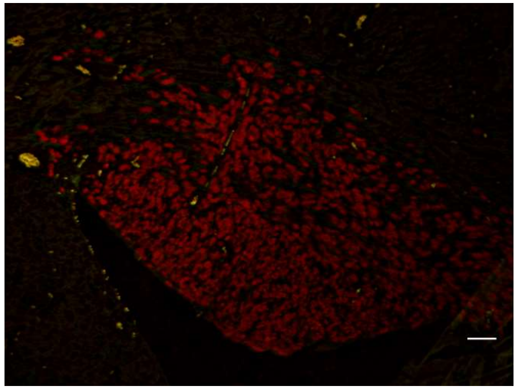

T1442 – invasive region

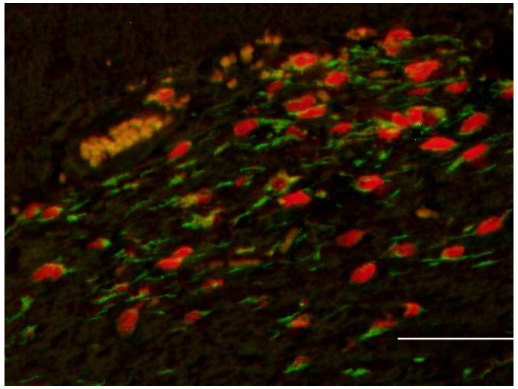

T1371 – invasive region

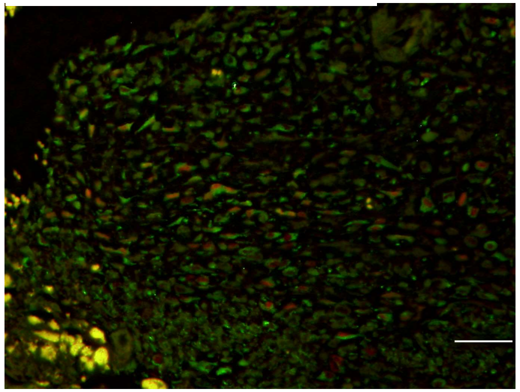

bar: 50 µm; z-stacks – Note: DAPI channel was omitted from some photos

SLGC line	P53 status	No	Type I-, II- or III-cells °	Tumor appearance	Size of tumor after HE stain
T1338	WT	6 #	Type I, II & III cells	Single Sox2++ human cells are present in IHC	No tumor
		8 §	Type I, II & III cells	Single Sox2++ human cells are present in IHC	No tumor
T1338 cl 1	WT	4 §	> 90% type I cells	Single Sox2++ human cells are present in IHC	No tumor
T1338 cl 7	WT	4 §	> 90% type I cells	Single Sox2++ human cells are present in IHC	No tumor
T1338 cl 6	WT/mut	4 §	> 90% type I cells	Single Sox2++ human cells are present in IHC	No tumor
T1440	WT	11	Type I, II & III cells	variably size, spreading into left hemisphere	500 – 2500 µm
T1440 clones	WT	12	> 90% type I cells	sharply bounded, small size	100 – 500 µm
T1464	WT	6	Type I, II & III cells	Sharp border, large necrotic center, spreading	> 1000 µm
T1464 clones	WT	12	Type I, II & III cells	Sharp border, large necrotic center, spreading	> 1000 µm
T1586	WT	6	> 98% type I cells	Small tumors, sharply bounded	100 – 500 µm
T1587	WT	6	> 90% type I cells	Small tumors, sharply bounded	100 – 500 µm
T1467	WT	9	Type I, II & III cells	Highly variably size, spreading into left hemisphere	500 – 5000 µm
T1439	WT	5	floating single cells; could not be stained by ICC; IHC was not done		No tumors
T1522	WT	5	> 90% type I cells	sharply bounded, small size	100 – 500 µm

No, number of mice; #, indicates that two animals were xenotransplanted with 50,000, 100,000, and 200,000 cells each. All other experiments were carried out with 200,000 cells. § animals were sacrificed after 6 months.
°, presence of type I-, II- and III- cells at stage of inoculation;
type I cell: Sox2 high/CD133-; type II cell: Sox+/CD133+; type III cell: Sox2 +/CD133- according to Chen et al., 2010.

SLGC line	P53 status	No	Type I-, II- or III-cells	Tumor appearance	Size of tumor after HE stain
T1389	GOF – R175H	10	Type I, II & III cells	Invasive, spreading into left hemisphere	500 – 1500 µm (invasive)
T1371	GOF – R175H	6 #	Type I, II & III cells	Invasive, spreading into left hemisphere	>> 1000 µm (invasive)
T1371	GOF – R175H	5	Type I, II & III cells	Invasive, spreading into left hemisphere	>> 1000 µm (invasive)
T1371 cl16	GOF – R175H	5	> 60% type I cells	intermediate size, still invasive	> 500 µm (invasive)
T1442	missense	16	Type I, II & III cells	Highly variable tumor size	100 µm → throughout brain
T1447	GOF – 248W	15	Type I, II & III cells	very large, highly invasive, spreading into left hemisphere	5000 → throughout brain
T1447 cl4	GOF – 248W	6	> 60% type I cells	Smaller than for T1447, but still invasive	500 - 1000 µm
T1495	GOF – R273H	6	Type I, II & III cells	Invasive, spreading into left hemisphere; necrotic center	5000 → throughout brain
T1495-SCID	GOF – R273H	6	Type I, II & III cells	Invasive, spreading into left hemisphere; necrotic center	5000 → throughout brain
T1452	Splice mutant	4	Type I, II & III cells	Highly variable tumor size	500 – 5000 µm
T1452 cl 10	Splice mutant	4	> 70% type I cells	Small tumors, sharply bounded; often entering ventricles	100 – 500 µm

No, number of mice;
#, indicates that two animals were xenotransplanted with 50,000, 100,000, and 200,000 cells each. All other experiments were carried out with 200,000 cells.
°, presence of type I-, II- and III- cells at stage of inoculation;
type I cell: Sox2 high/CD133-; type II cell: Sox+/CD133+; type III cell: Sox2 +/CD133- according to Chen et al., 2010.

Supplementary: Expression of pten **mRNA** (RT-PCR) – PCR products were separated on 1.6% TBE agarose gels. The samples -1 and -2 are biological replicates - - T1439-F was grown in medium containing FCS.

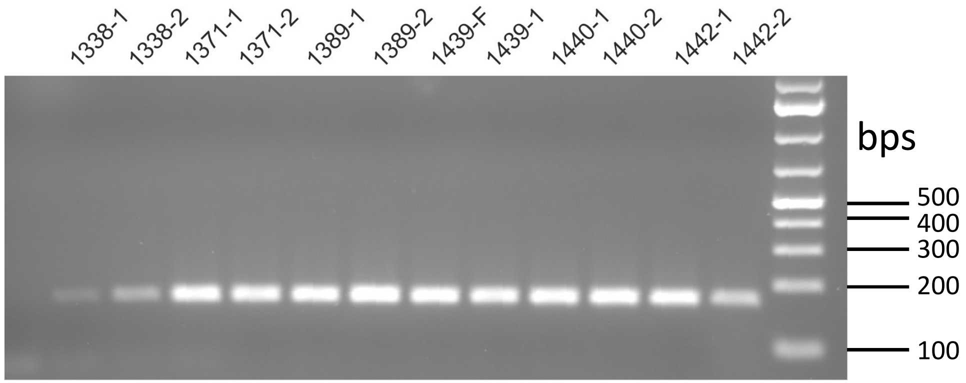

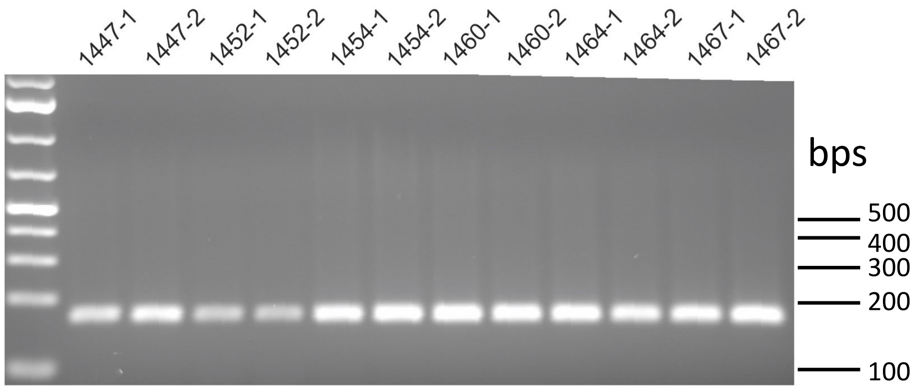

<u>Negative for PTEN protein</u>:

- T1338
- T1371
- T1389
- T1442
- T1447
- T1452
- T1467

<u>Positive for PTEN protein</u>:

- T1439
- T1454
- T1464

<u>Mixed status: </u>T1440 (T1440 clones are +, - or +/- for PTEN protein)

Please note: This analysis encompassed one additional SLGC lines (T1460), which was not further used in present study.

Supplementary: Expression of Dlx2 *(Western blot analysis)* – 10 % SDS-PAGE; transfer using semi-dry technique

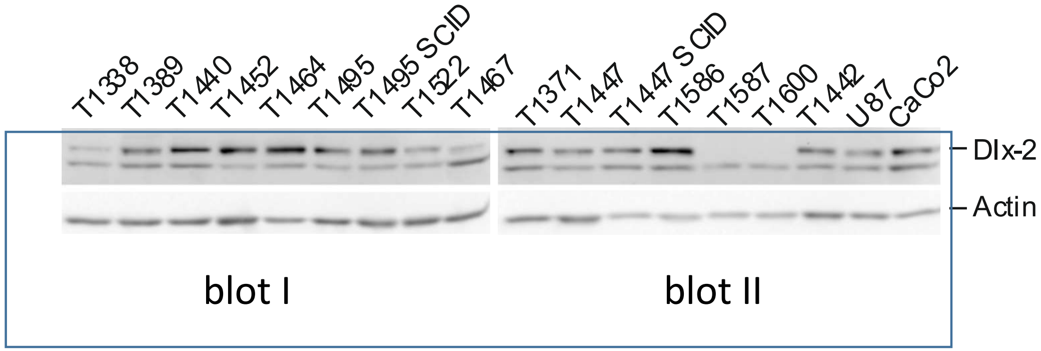

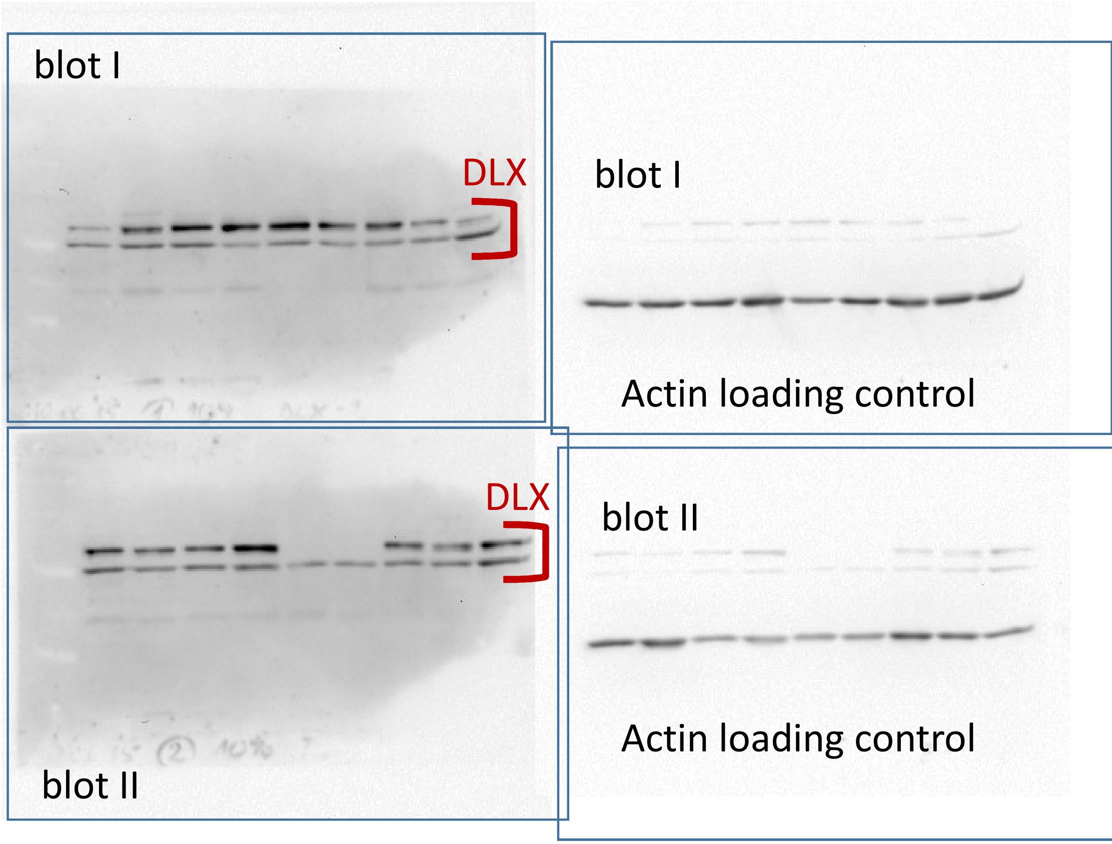

DLX: Distal-Less Homeobox 2

Supplementary: *Expression of Integrins (Western blot analysis)*
– 10 % SDS-PAGE;
transfer using „wet-blot“ technique

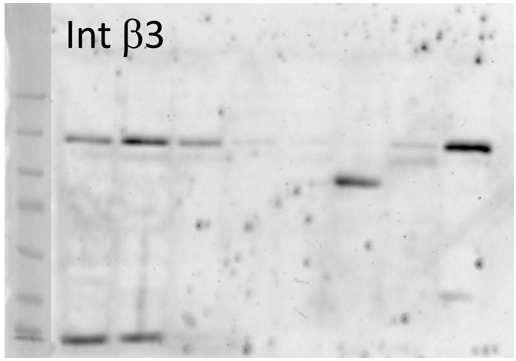

Integrin β3
130 kDa
110 kDa
97 kDa

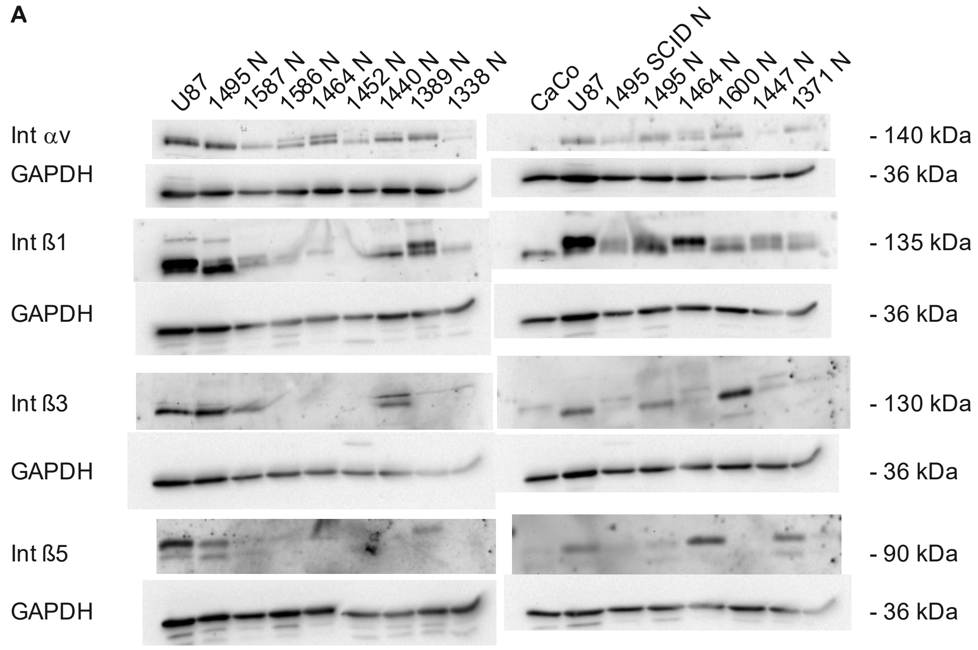

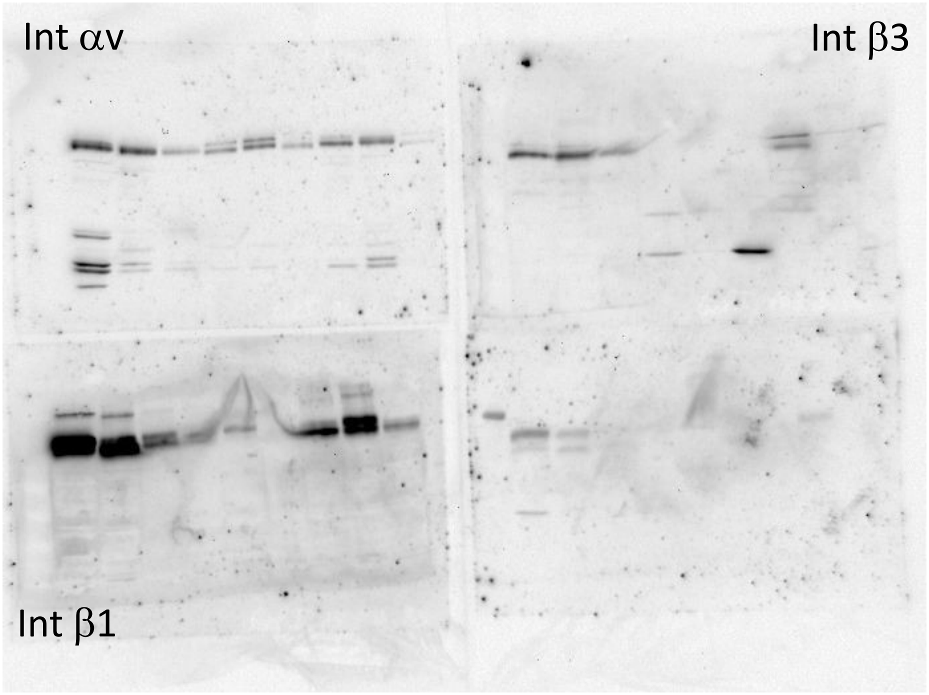

GAPDH: loading control

Western blot: Integrin α5

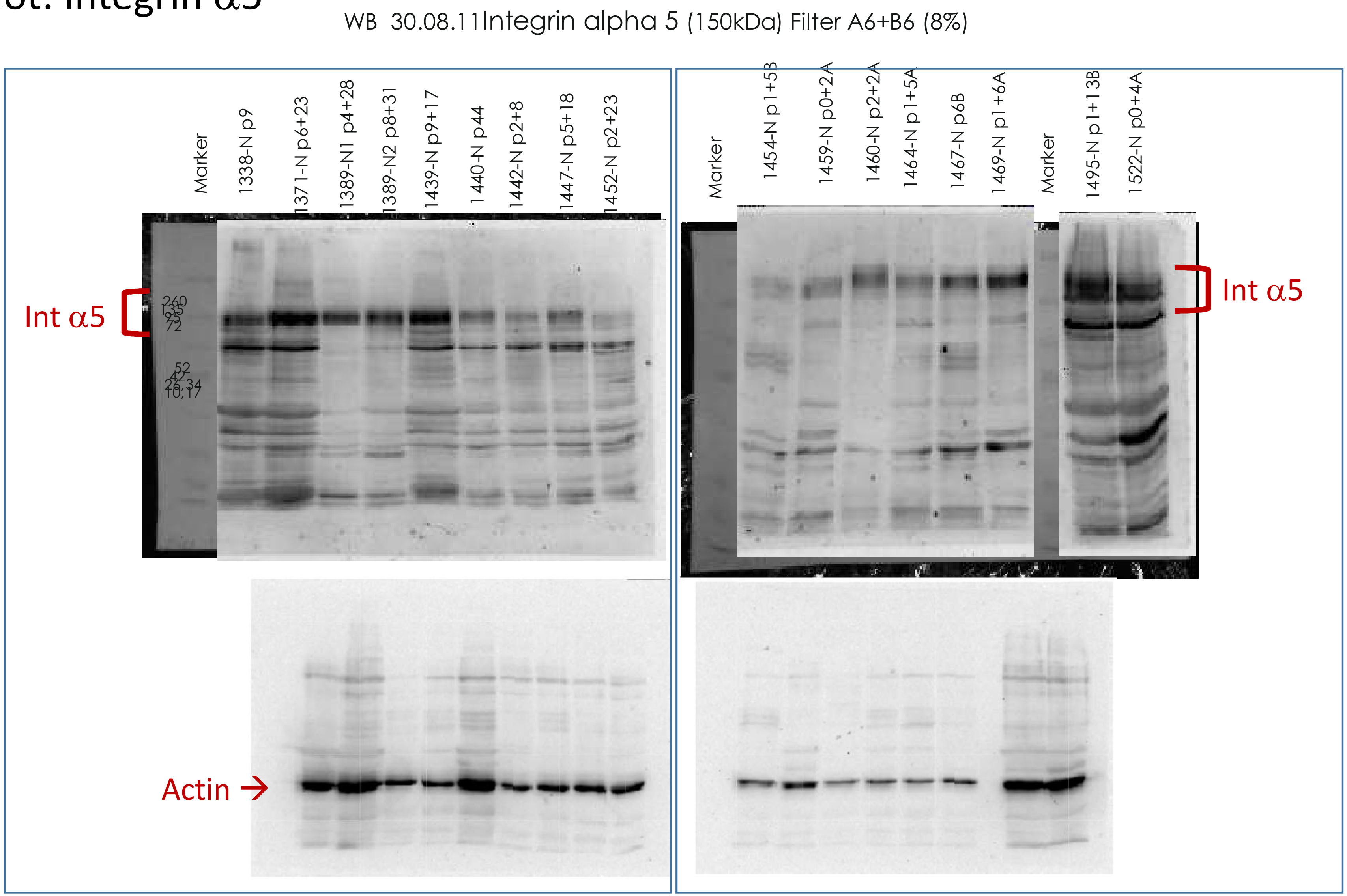

Western blot: Integrin β1

WB 26.08.11Integrin beta 1 (115,135kDa) Filter A2+B2 (10%Gel)

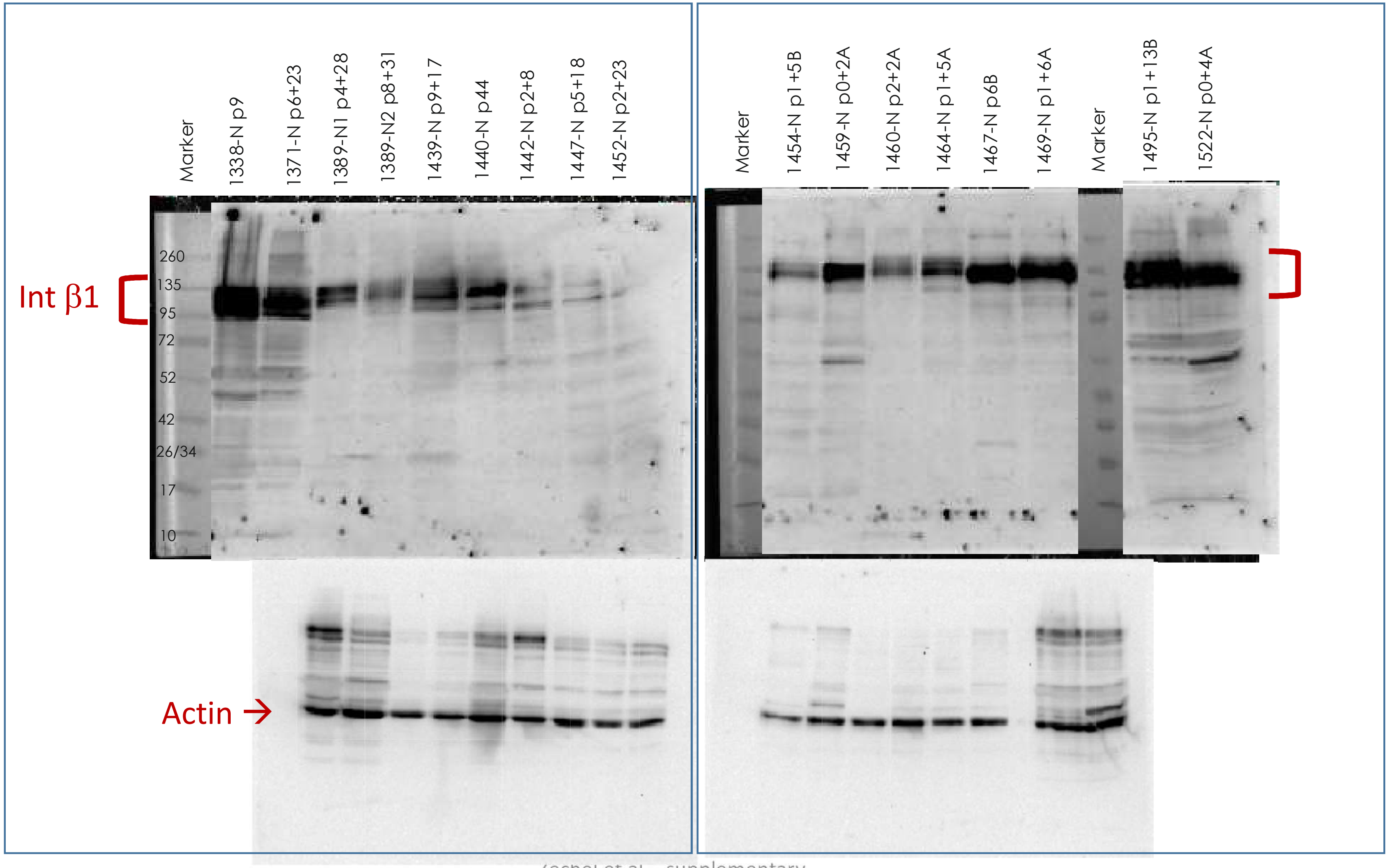

Western blot: Integrin β3

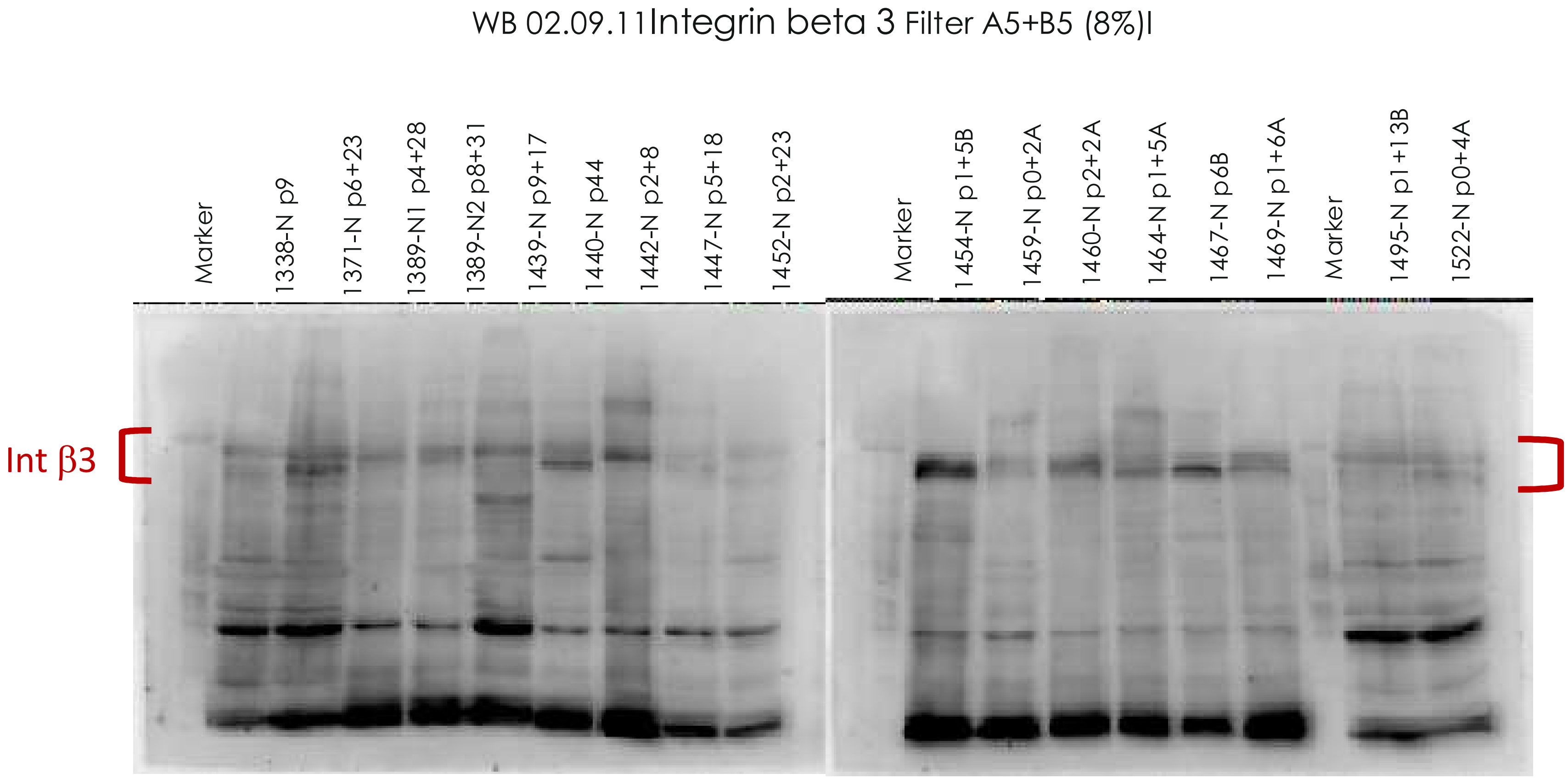

Western blot: N-Cadherin

WB 30.08.11N-Cadherin (140kDa) Filter A5+B5 (8%)

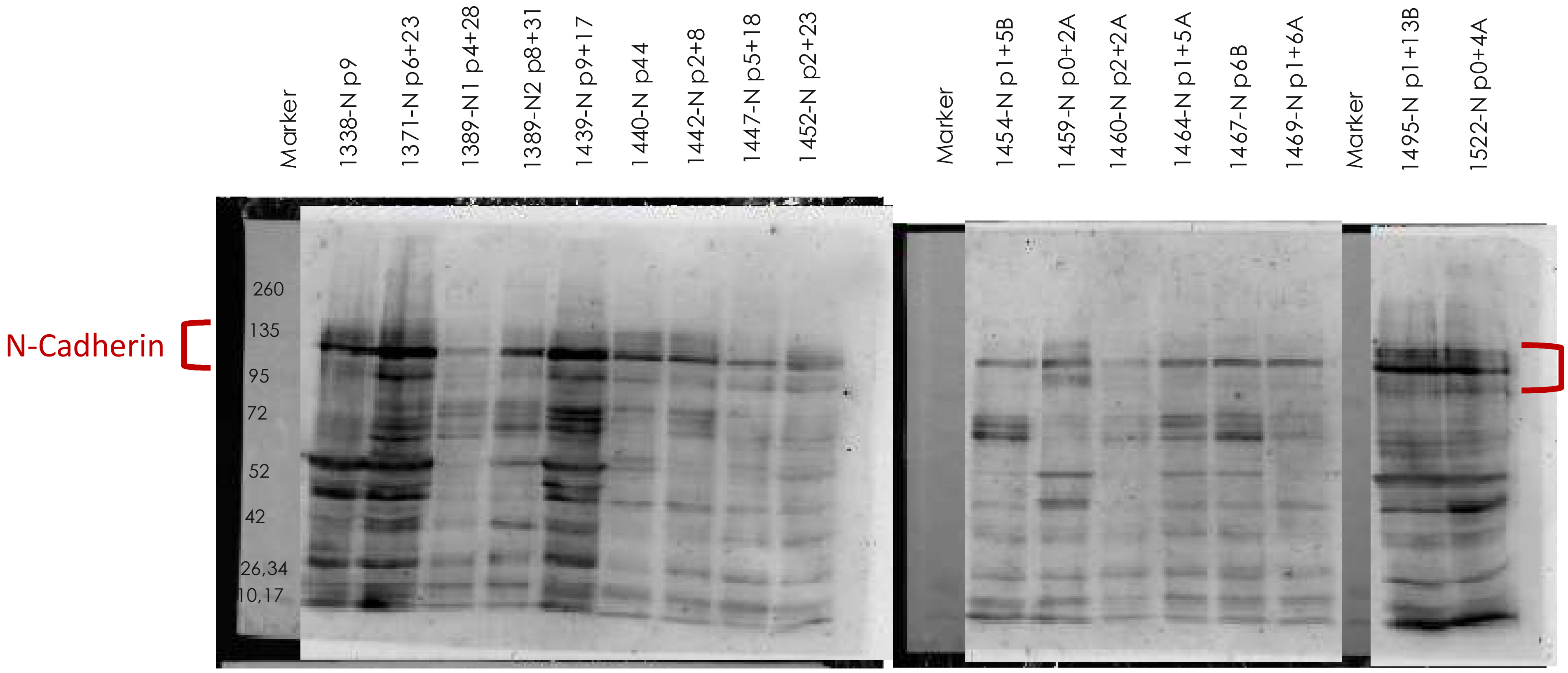

Western blot: E-Cadherin

WB 02.09.11 E-Cadherin Filter A6+B6 (8%)

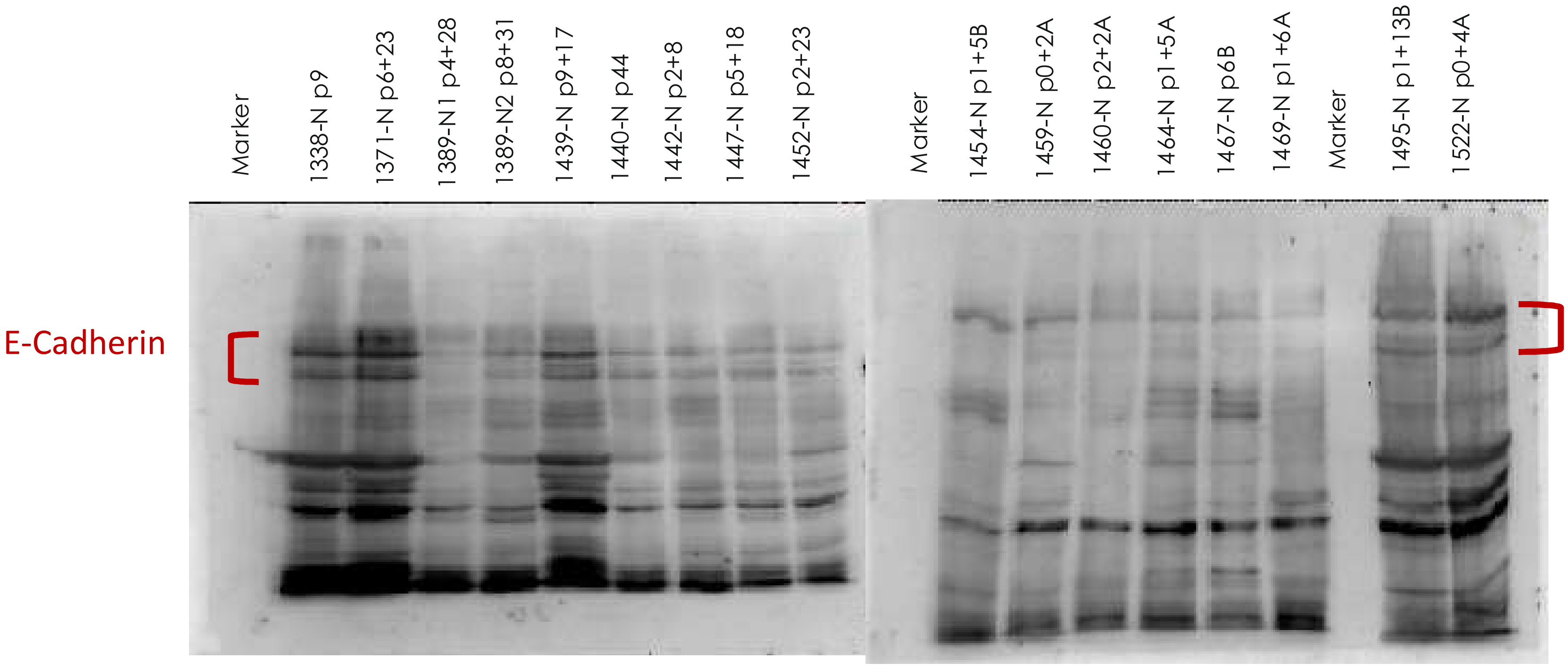

Supplementary: Expression of EGFR

Upper: Expression of egfr mRNA (RT-PCR analysis). – PCR products were separated on 1.6 % TBE agarose gels.

Lower: Western blot analysis – 10 % SDS-PAGE; transfer using semi-dry technique

egfr/EGFR: epidermal growth factor receptor

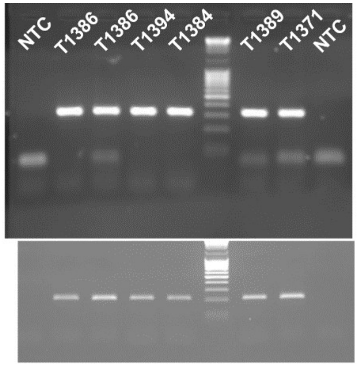

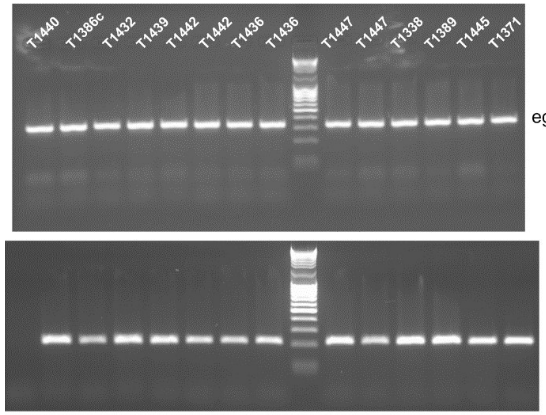

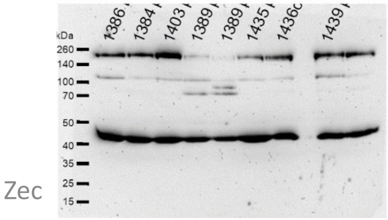

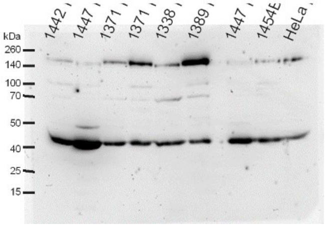

Supplementary: EGFR expression (Western blot analysis) – 8% SDS PAGE. - SLGC lines (mc) and clones derived from the mc cultures by limited dilution assays were analyzed. – Amplification of truncated EGFR was observed for the T1464 mc and several T1464 clones, but disappeared over time in both.

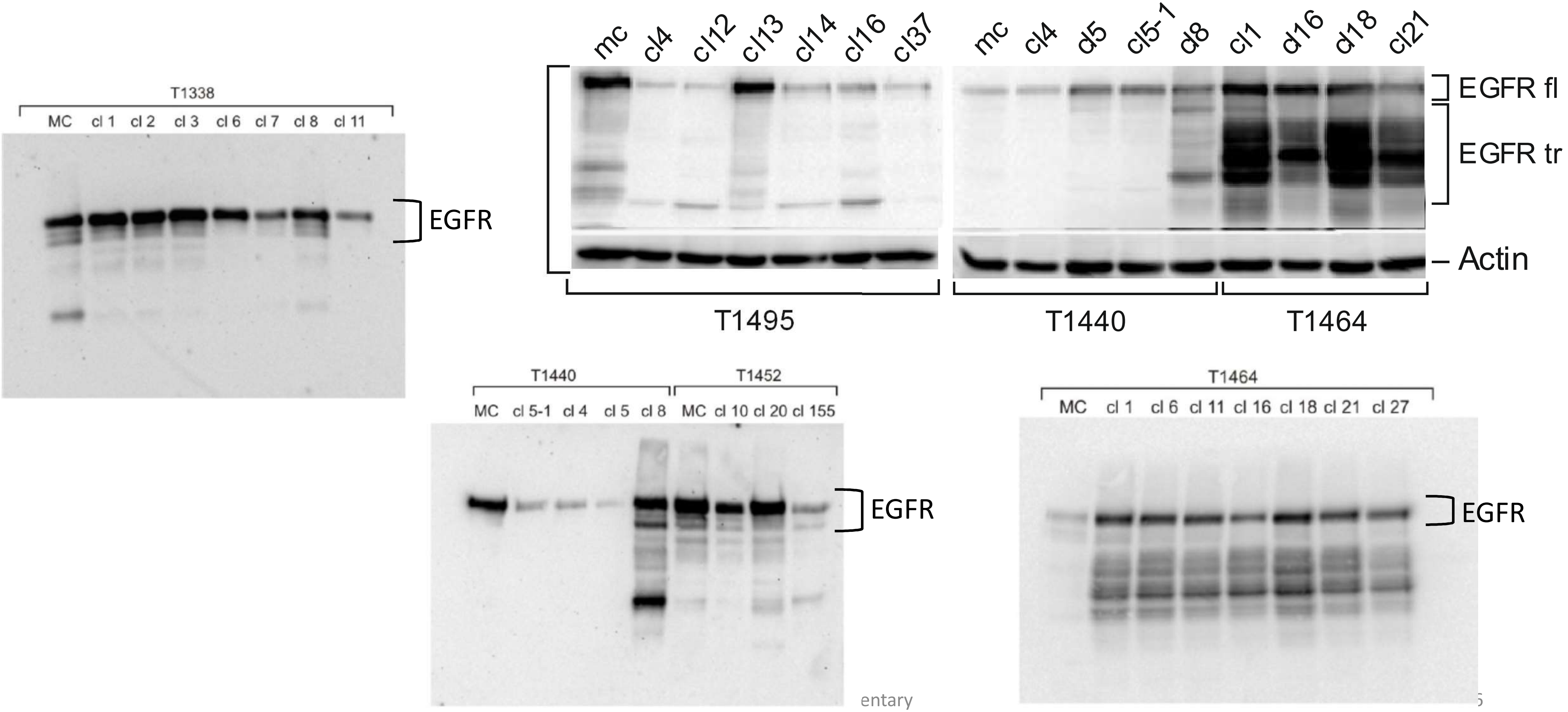

Supplementary: relative number of CD133-positive cells in the cultures indicated and changes over progressing passages (flow cytometry analysis).

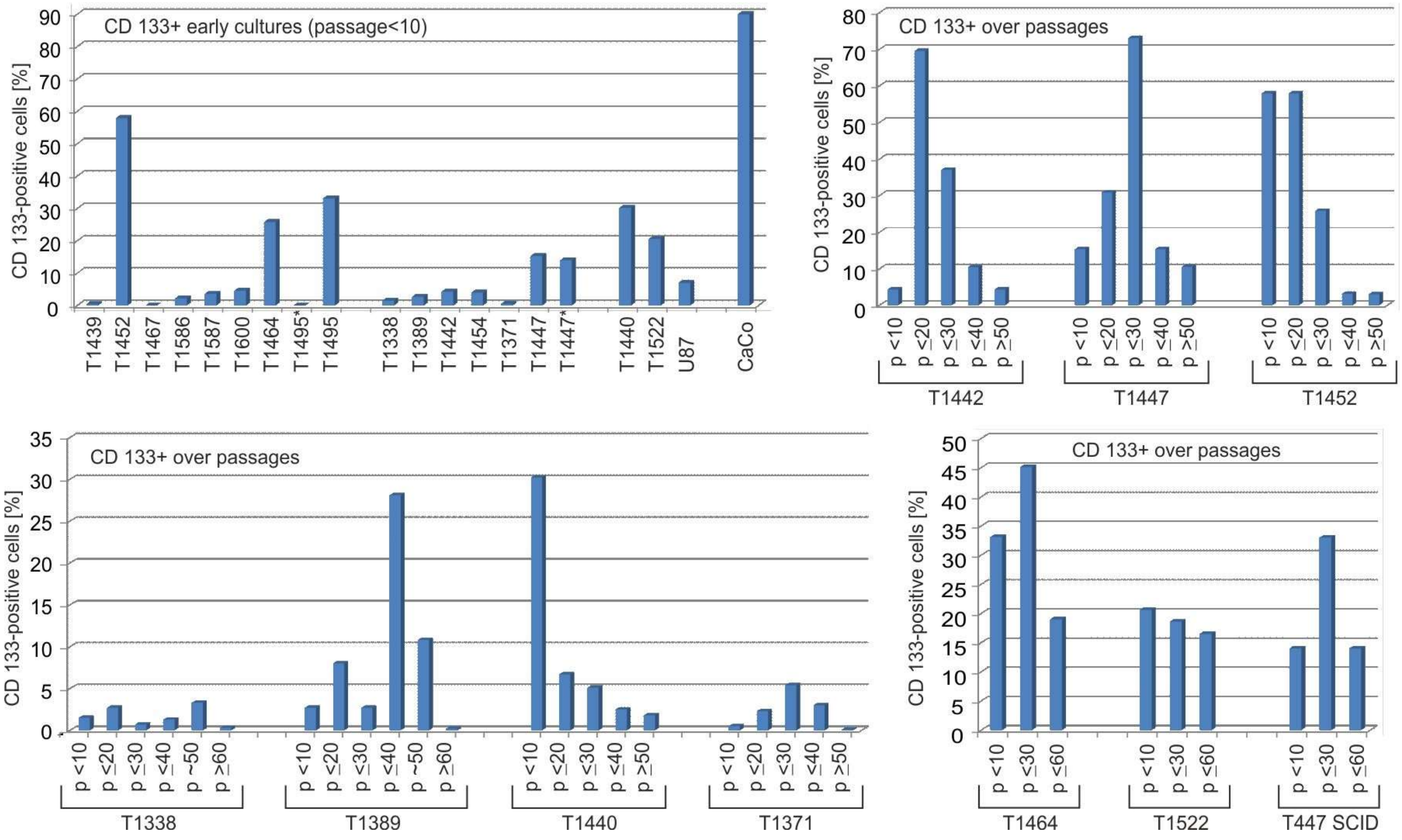

Supplementary: relative number of Integrin α6- or αv-positive cells in the cultures indicated (flow cytometry analysis). Two distinct biological replicates (I and II) were studied; cells were grown in N-medium.
Analyses with the Integrin α6- or αv- antibodies were carried out in parallel.

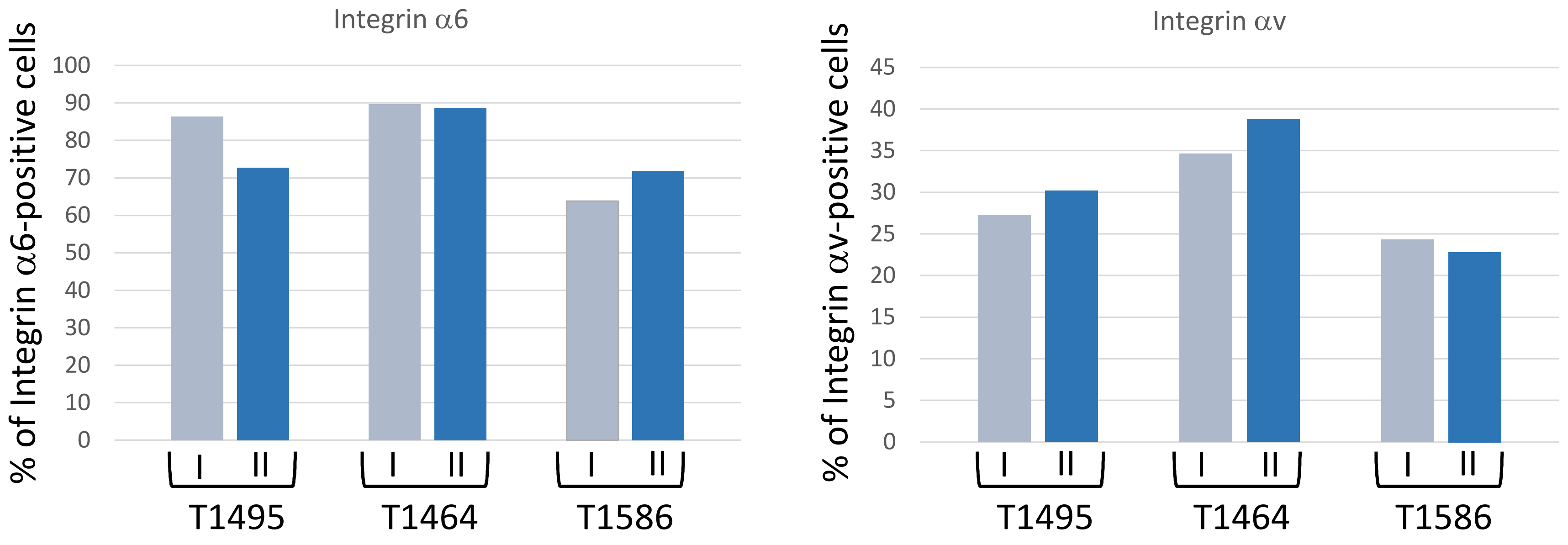

Supplement: S1 Appendix — (PDF) [file pone.0291368.s012.pdf]
